# Identification of *Fusarium oxysporum* f. sp. *cubense* tropical race 4 (*Foc* TR4) responsive miRNAs in banana root

**DOI:** 10.1038/s41598-019-50130-2

**Published:** 2019-09-23

**Authors:** Chunzhen Cheng, Fan Liu, Xueli Sun, Na Tian, Raphael Anue Mensah, Dan Li, Zhongxiong Lai

**Affiliations:** Institute of Horticultural Biotechnology/College of Horticulture, Fujian Agriculture and Forestry University, Fuzhou, 350002 China

**Keywords:** miRNAs, Biotic

## Abstract

The fungus, *Fusarium oxysporum* f. sp. *cubense* (*Foc*), is the causal agent of Fusarium wilt disease, which is the most serious disease affecting the whole banana industry. Although extensive studies have characterized many *Foc*-responsive genes in banana, the molecular mechanisms on microRNA level underlying both banana defense and *Foc* pathogenesis are not yet fully understood. In this study, we aimed to reveal the role of miRNA during banana-*Foc* TR4 interactions. Illumina sequencing was used to reveal the changes in small RNAome profiles in roots of *Foc* TR4-inoculated ‘Tianbaojiao’ banana (*Musa acuminata* cv. Tianbaojiao) in the early stages (i.e. 5 h, 10 h and 25 h post *Foc* TR4 inoculation, respectively). The expression of some differentially expressed (DE) miRNAs and their predicted target genes was studied by using quantitative real time PCR (qRT-PCR). Totally, 254 known miRNAs from 31 miRNA families and 28 novel miRNAs were identified. Differential expression analysis identified 84, 77 and 74 DE miRNAs at the three respective *Foc* TR4 infection time points compared with control healthy banana (CK). GO and KEGG analysis revealed that most of the predicted target genes of DE miRNAs (DET) were implicated in peroxisome, fatty acid metabolism, auxin-activated signaling pathway, sulfur metabolism, lignin metabolism and so on, and many known stress responsive genes were identified to be DETs. Moreover, expected inverse correlations were confirmed between some miRNA and their corresponding target genes by using qRT-PCR analysis. Our study revealed that miRNA play important regulatory roles during the banana-*Foc* TR4 interaction by regulating peroxidase, fatty acid metabolism, auxin signaling, sulfur metabolism, lignin metabolism related genes and many known stress responsive genes.

## Introduction

Fusarium wilt or Panama disease, the most destructive, notorious and still uncontrollable fungal disease of banana caused by soil-borne *Fusarium oxysporum* f. sp. *cubense* (*Foc*), has devastated and continues to threaten banana production worldwide^1,2^. Currently, *Foc* has been classified into 3 races according to their banana hosts, i.e., *Foc* race 1 (*Foc* 1), *Foc* race 2 (*Foc* 2) and *Foc* race 4 (*Foc* 4). *Foc* 4 can be further divided into *Foc* tropical race 4 (*Foc* TR4) and *Foc* subtropical 4 (*Foc* STR4). Among all the *Foc* biotypes, *Foc* TR4 was considered as the most predominant and destructive one for its much wider range of hosts (can invade Cavendish banana and all the cultivars that are sensitive to the other 3 *Foc*s) and distributions (stronger high temperature tolerance) than the others^3^.

The only practical strategy for controlling Panama disease is by using resistant varieties^4^. Although sources of resistance to *Foc* 4 have been found in wild banana species^5^, it has not been possible to introgress this resistance into current commercial cultivars by conventional breeding mainly due to the sterile nature of most polyploid banana cultivars^6^. Genetic transformation in banana offers the opportunity to overcome this obstacle^7,8^. To date, however, limited banana *Foc* resistance related genes have been isolated^9,10^.

RNA Seq and digital gene expression (DGE) analysis, powerful methods for analyzing the plant transcriptome, have been used for investigating the transcriptional changes that occur during the response of banana to *Foc* TR4. For example, Li *et al*.^11^ compared the transcriptome changes of a resistant Cavendish banana mutant ‘Brazilian’ and its susceptible wild-type ‘Nongke No 1’ by using RNA-Seq and digital gene expression analysis, and found that the banana basal defense mechanism participate in the recognition of pathogen-associated molecular pattern (PAMP) and the defense-related transcripts contribute to the *Foc* TR4 resistance of the resistant Cavendish banana mutant. Wang *et al*.^12^ compared the transcriptome differences of ‘Brazilian’ banana at 4 time points post *Foc* TR4 infection, i.e. 0, 2, 4, and 6 days post inoculation (dpi) by using DGE analysis, and found that genes involved in the phenylalanine metabolism, phenylpropanoid biosynthesis and alpha-linolenic acid metabolism pathways showed significant expression changes, which indicated that these genes might be essential for the banana response to *Foc* TR4 infection. Li *et al*.^13^ compared the early stage transcriptome changes of ‘Brazilian’ banana after *Foc* 1 and *Foc* TR4 infection using DGE, and found that the global gene expression changes caused by the two *Foc*s’ infection were very similar and also discovered the significant activation of ethylene biosynthesis and signaling related gene. Bai *et al*.^14^ investigated the gene expression profile changes of the high resistant banana cultivar ‘Yueyoukang 1’ and *Foc* TR4 susceptible ‘Brazilian’ at 0.5, 1, 3, 5 and 10 dpi with *Foc* 4, and faster and stronger defense response and as well as milder HR and senescence reactions were recorded in the high resistant cultivar. Deng^15^ compared the transcriptome changes of the high resistant ‘Kangku No. 5’ and susceptible ‘Tianbaogaojiao’ and found that plant defense related genes such as genes encoding some transcription factors, SA, JA and ethylene signaling related proteins, phenylalanine metabolism and cell wall lignification related proteins were more significantly induced in ‘Kangku No. 5’, indicating that these genes might contribute to its high resistance.

Small RNAs (sRNA) are a distinct class of regulatory non-coding RNAs in plants and animals^16^. The majority of them are short-interfering RNAs (siRNA) and microRNAs (miRNA) involving in gene silencing. Many plant miRNAs exert their critical regulatory roles through interacting specifically with their target mRNAs to suppress their activities^17,18^. Recently, accumulated evidences have suggested that sRNAs are involved in plant-pathogen interactions^19–21^. For banana, however, miRNAs have only been studied in relation to fruit ripening^22^, root salt stress response^23^, variety miRNAome difference^24^ and genome wide miRNA identification^25–28^, no research has been reported on the miRNAs involving in banana-*Foc* interaction. Therefore, in the present study, to reveal the role of miRNAs in banana in response to *Foc* TR4 infection, Illumina sequencing was applied to reveal the changes in sRNAome profiles in roots of *Foc* TR4-inoculated ‘Tianbaojiao’ banana (*Musa acuminata* cv. Tianbaojiao) in the early stage (i.e. 5 h, 10 h and 25 h post *Foc* TR4 inoculation, respectively). The results generated in this study would be very helpful for the understanding of the complex roles of banana miRNAs in response to *Foc* TR4 infection in the early stage and could provide basis for the exploration of the miRNA regulated *Foc* TR4 responsive genes.

## Materials and Methods

### Plant materials and pathogen preparation

The tissue cultured ‘Tianbaojiao’ banana plants used in this study were provided by Institute of Horticultural Biotechnology, Fujian Agriculture and Forestry University, Fuzhou, China. Tissue cultured seedlings were moved to nutrient soil (FAFARD^®^ #1VC, Canada) after four weeks’ rooting and two-day’s hardening. Then, the plants were kept at 28 °C, 60~80% relative humidity, a photoperiod of 12 h (1500 ± 200 lx). After about 6 weeks, plants with five leaves and healthy root system were moved to the modified Hoagland solution for liquid culture for about 1 week.

The GFP-labeled *Foc* TR4 was provided by College of Plant Protection, Fujian Agriculture and Forestry University, Fuzhou, China. After seven-day’ incubation at 28 °C in dark on PDA medium, fresh *Foc* TR4 was washed out by using sterilized water. After filtering by using sterilized six-layer cheese cloth to remove the mycelium, the spore solution concentration was adjusted to 5 × 10^6^ chlamydospores/ml for pathogen inoculation.

### Determining the time-points for harvesting the samples and *Foc* TR4 inoculation

To determine the sample harvesting time-point, the GFP-labeled *Foc* TR4 progression in banana roots was monitored under a confocal microscope (Olympus, FV1200) according to Li *et al*.^11^. At about 5 hours post inoculation (hpi), many chlamydospores were attached to the banana root. At 10 hpi, after water rinsing, chlamydospores could also be observed in root samples and chlamydospores began to germinate and develop into hyphae. At 25 hpi, many fungus began to be detected in the vascular tissues. To analyze the sRNA expression changes caused by *Foc* TR4 infection, the four time points, i.e. 0 hpi, 5 hpi, 10 hpi and 25 hpi, were selected for RNA-Seq analysis of the root sRNAomes.

After the harvesting time determination, the liquid cultured banana seedlings were divided into four groups: group I (R25), group II (R10) and group III (R5H) was respectively inoculated with GFP-labeled *Foc* TR4 25 h, 10 h and 5 h before root harvesting, group IV banana plants that were not inoculated with the *Foc* TR4 and their roots were served as control (RCK). Three seedlings were used for each treatment and all the root samples were harvested at the same time and washed with sterilized water to remove the attached chlamydospores and hyphae. Then, root samples were precooled in liquid nitrogen and stored at −80 °C freezer for further use.

### SRNA sequencing and sequence processing

Total RNA was extracted by using the QIAGEN RNeasy plant mini kit (QIAGEN, Valencia, CA), and then was treated with RNase free DNase I (Promega, Madison, Wisconsin, USA). RNA degradation and contamination, purity, concentration and integrity was monitored or measured by using 1% agarose gel electrophoresis, NanoPhotometer® spectrophotometer (IMPLEN, CA, USA), Qubit® RNA Assay Kit in Qubit® 2.0 Flurometer (Life Technologies, CA, USA) and the Agilent Bioanalyzer 2100 system (Agilent Technologies, CA, USA) respectively. To reduce the influence of individual background differences, high-quality root RNA of the 3 seedlings from each group were equiweightly mixed and were sent to Novogene Bioinformatics Technology Co. Ltd. for sRNA sequencing. For small RNA library construction, a total amount of 3 μg total RNA was used. By using NEBNext® Multiplex Small RNA Library Prep Set for Illumina® (NEB, USA.), sRNA sequencing libraries were constructed according to the manufacturer’s manual. After library quality assessment, the libraries were sequenced on the Illumina Hiseq2500 platform and 50 bp single-end reads were generated.

### Small RNA annotation and miRNA identification

After removing the low quality reads containing ploy-N, with 5′ adapter contaminants, without 3′ adapter or the insert tag, containing ploy A or T or G or C and low quality reads from raw data, the clean data were obtained and were used for further analysis. The clean small RNA tags were firstly mapped to reference banana genome (http://banana-genome.cirad.fr/content/download-dh-pahang) by using Bowtie^29^ with no mismatch allowed to analyze their expression and distribution. The sRNA tags matched to protein-coding genes, repeat sequences, rRNA, tRNA, snRNA, and snoRNA, small RNA tags were then removed. The left genome mapped unique sRNA were used to search for known miRNAs by blasting against miRBase 21.0 database using modified software mirdeep2. To explore the occurrence of miRNA families, miFam.dat (http://www.mirbase.org/ftp.shtml) was used to look for miRNA families. The remaining unannotated sRNAs were then subjected to novel miRNA prediction using miREvo^30^ and mirdeep2^31^ through exploring the secondary structure, the Dicer cleavage site and the minimum free energy^22^.

### Identification of the *Foc* TR4 responsive miRNAs and target gene prediction

For the identification of the *Foc* responsive miRNA or differentially expressed miRNA (DE miRNA), the read number of each miRNA was firstly normalized to TPM (transcript per million). MiRNA with |log2 (fold change)| >1 and qvalue <0.01were identified as differentially expressed miRNA (DE miRNA). The target genes of each miRNA were predicted according to the method described by Bi *et al*.^22^ using psRobot^32^. GO and KEGG enrichment analysis of the predicted target genes were further performed for the elucidation of the way miRNA participate in the banana response to *Foc* TR4 infection according to Bi *et al*.^22^.

### Quantitative real-time PCR analysis for miRNAs and their target genes

Total RNA of the three seedlings of each treatment was used for the reverse transcription reactions of the miRNAs and their corresponding target genes by respectively using TransScript miRNA First-Strand cDNA Synthesis SuperMix (Trans) and PrimeScript® RT reagent Kit (TaKaRa). Then, qRT-PCR was adopted to confirm the sequencing result of thirteen selected miRNAs. These thirteen selected miRNAs include 2 miRNAs (mac-miR166a and mac-miR395b-3p) identified as DE miRNA in none of the three comparison and eleven miRNAs (mac-nmiR11, mac-nmiR21, mac-miR528-5p, mac-miR171a-3p.2, mac-miR408-3p.3, mac-miR156a.1, mac-miR393a.1, mac-miR397b.2, mac-miR167.1, mac-miR398 and mac-miR5658) identified as DE miRNA in at least one comparison. Poly(A) Tailing Kit (Ambion) was used to extend the 3’-end of all sRNAs, and the following PAP real-time quantitative PCR (PAP qRT-PCR) was performed on Lightcycler® 480II (Roche, Switzerland) using the SYBR® Premix Ex Taq^TM^ II (Perfect Real Time) kit^33^, and *U6* was used as the internal control. PCR conditions were as follows: initial denaturation at 95 °C for 1 min; followed by 45 cycles of 95 °C for 20 s, 54~60 °C for 20 s, and 72 °C for 20 s. For melting curve analysis, the fluorescence intensity data were collected in the range of 65~95 °C with a rate of 0.5 °C per 10 s at the end of the run. Three biological and three technical replicates were used for analyzing each miRNA to minimize quantification errors. Relative expression analysis of these miRNA were calculated by using the 2^−ΔΔCT^ method according to Bi *et al*.^22^. To check the expression pattern of target genes, the expression analysis of the target genes of selected miRNAs were also determined by qRT-PCR using *GAPDH* (*Glyceraldehyde*-*3*-*phosphate dehydrogenase*) and *EIF5A*-*2* (*Eukaryotic initiation factor 5A*-*2*) as the endogenous controls according to Chen *et al*.^34^. Fifteen target genes, including the *zeta-carotene desaturase* (*ZDS*) gene (target of mac-miR166a), the *putative disease resistance protein RPM1* (*RPM1*) gene (target of mac-miR395b-3p), the *jasmonate ZIM-domain protein* (*JAZ*) gene (target of mac-nmiR11), the *protein cup-shaped cotyledon 2* (*CUC2*) gene (target of mac-nmiR21), the *polyphenol oxidase* (*PPO*) gene (target of mac-miR528-5p), the *scarecrow like protein 27* (*SCL27*) gene (target of mac-miR171a-3p.2), the *transcription factor TGA21* (*TGA21*) gene (target of mac-miR408-3p.3), the *putative selenium-binding protein* (*SBP*) and the *superoxide dismutase* [*Cu-Zn*] (*SOD*) gene (targets of mac-miR398), *the putative squamosa promoter-binding-like protein 16* (*SPB16*) gene (target of mac-miR156a.1), *the laccase-25* (*LAC*) gene (target of mac-miR397b.2), the *putative regulatory protein NPR1* (*NPR1*) and the *glutathione S-transferase zeta class* (*GST*) (targets of mac-miR5658), the *transport inhibitor response 1-like protein* (*TIR*) gene (target of mac-miR393a.1), and the *auxin response factor 12* (*ARF12*) gene (target of mac-miR167.1) were simultaneously analyzed for their expression levels using qRT-PCR. Each reaction (20 µl) included 10 µl of SYBR® Premix Ex Taq™ II (Tli RNaseH Plus) (Takara, Japan), respectively 0.8 µl of forward and reverse primer, 1 µl cDNA template and 7.4 µl nuclease-free water.

QRT-PCR conditions of the target genes were as follows: initial denaturation at 95 °C for 1 min; followed by 40 cycles of 95 °C for 20 s, 58 °C for 20 s, and 72 °C for 20 s. For melting curve analysis, the fluorescence intensity data were collected in the range of 65~95 °C with a rate of 0.5 °C per 10 s at the end of the run. All the primers used in this study were listed in Supplemental Data S1. Gene expression levels were calculated based on the normalization factors (NFs) produced by GeNorm according to Zhou *et al*.^35^.

## Results

### Small RNA profile changes revealed by high-throughput sequencing in *Foc* TR4 inoculated banana roots

**Figure 1:**
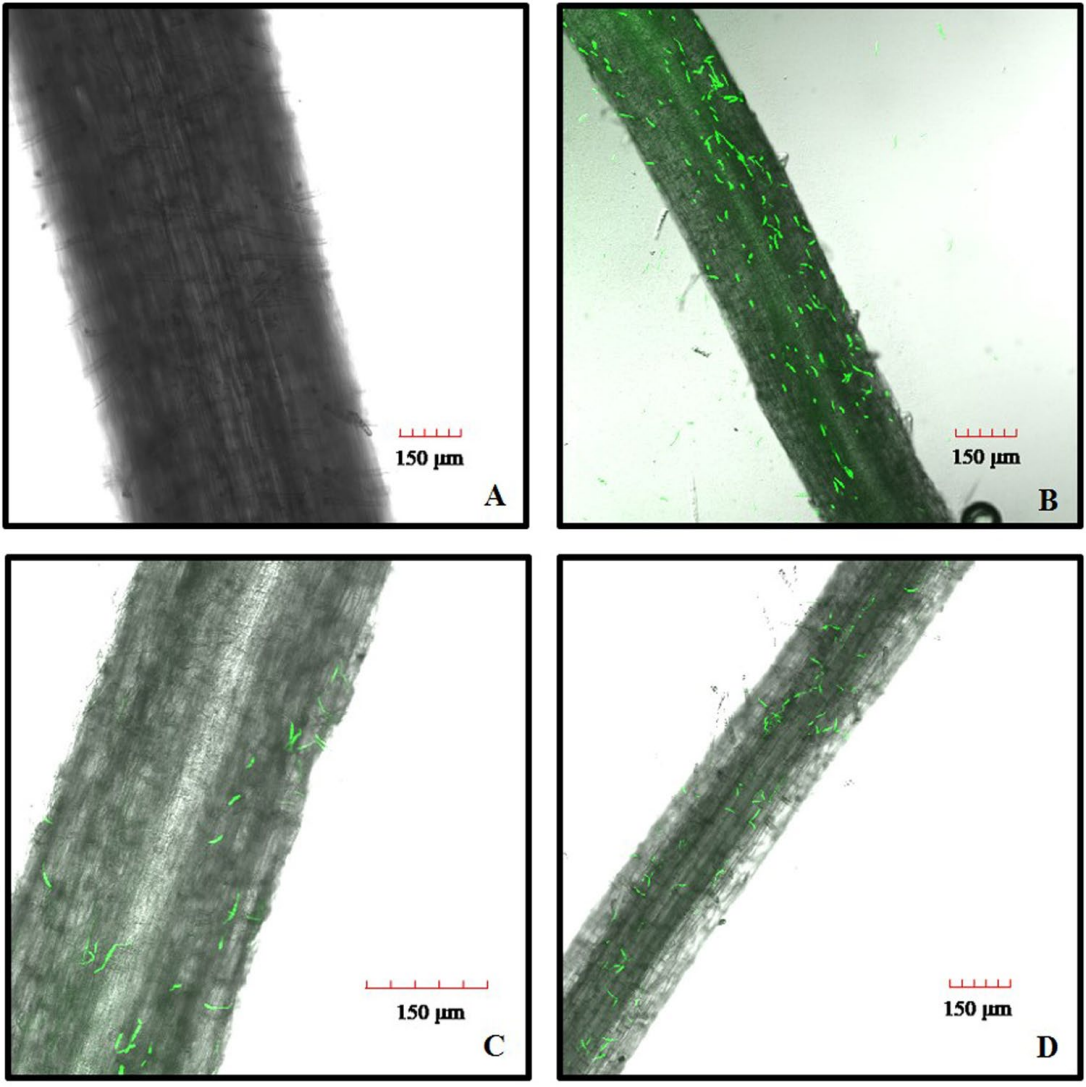
The observation results of GFP-labeled *Foc* TR4 on the lateral roots of ‘Tianbaojiao’ banana for RCK (**A**) R5H (**B**) R10 (**C**) and R25 (**D**). For the control, no GFP was observed. (**A**) Many chlamydospores were attached on the roots ‘Tianbaojiao’ banana at 5 hpi. (**B**) After water rinsing, few germinated chlamydospores were observed on the roots of ‘Tianbaojiao’ banana at 10 hpi (**C**) and more were observed at 25 hpi (**D**). (**A**,**B**,**D**) were taken under 20× and (**C**) was taken under 40× magnification on the Olympus confocal microscope (FV1200).

**Figure 2:**
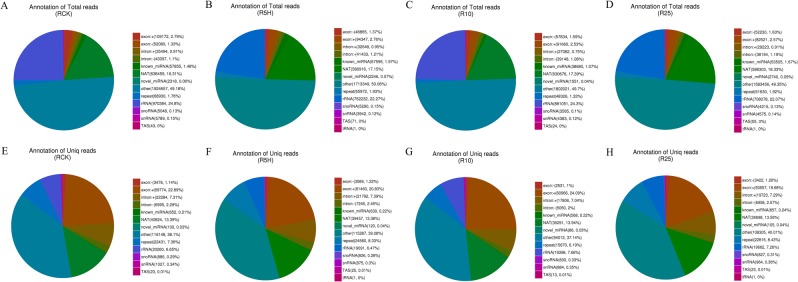
Category results of the genome mapped total sRNA (**A**–**D**) and unique sRNA (**E**–**H**) of the four banana root libraries. (**A**,**E**) The RCK library; (**B**,**F**) the R5H library; (**C**,**G**) the R10 library; (**D**,**H**) the R25 library. RCK, R5H, R10 and R25 represent the root samples treated with *Foc* TR4 for 0 h, 5 h, 10 h and 25 h, respectively.

To study the expression of banana sRNAs in response to *Foc* TR4 infection and to identify miRNAs involved in banana-*Foc* TR4 interaction, we inoculated the liquid cultivated banana seedlings with GFP-labeled *Foc* TR4 spore solution. According to the GFP detection result (Fig. 1), root samples treated by *Foc* TR4 for different time length (0 h, 5 h, 10 h and 25 h) were collected for RNA isolation and then for small RNA profiling. SRNA sequencing on Illumina Hiseq. 2500 produced 13,745,947, 13,694,126, 12,568,504 and 12,027,866 raw reads for RCK, R5H, R10 and R25 groups respectively (Table 1). About 90% of the total reads remained after removing the low quality reads and the unwanted reads below standards. There were 12,558,692, 11,934,390, 11,462,346 and 11,066,024 clean reads for RCK, R5H, R10 and R25 group, respectively. Then, the 18–30 nt small RNA sequences were selected out, and 6,063,504 (RCK), 5,335,626 (R5H), 5,377,388 (R10), 5,864,215 (R25) sRNA sequences were used for further analysis (Table 1). By blasting against the banana genome, 3,913,326 (64.54% of the sRNA in RCK), 3,422,908 (64.15% of the sRNA in R5H), 3,626,001 (67.43% of the sRNA in R10) and 3,208,726 (54.72% of the sRNA in R25) sequences of the 18–30 nt sRNAs were perfectly mapped to the genome (Fig. 2 and Table 2). The number of sRNA sequences identified as putative known miRNAs ranged from 38,680 to 67,596 and the number of sRNA sequences identified as novel miRNAs ranged from 1,551 to 2,740 (Fig. 2 and Table 2).

**Table 1:** Information of the sRNA sequences of the four banana root libraries.

Read Type	RCK	R5H	R10	R25
Total reads	13,745,947 (100.00%)	13,694,126 (100.00%)	12,568,504 (100.00%)	12,027,866 (100.00%)
N% >10%	0 (0.00%)	0 (0.00%)	0 (0.00%)	0 (0.00%)
Low quality	17,650 (0.13%)	16,272 (0.12%)	16,392 (0.13%)	22,712 (0.19%)
5′ adapter contamine	7,114 (0.05%)	8,626 (0.06%)	6,123 (0.05%)	6,737 (0.06%)
3′ adapter null or insert null	1,157,201 (8.42%)	1,730,149 (12.63%)	1,078,868 (8.58%)	926,527 (7.70%)
With ployA/T/G/C	5,290 (0.04%)	4,689 (0.03%)	4,775 (0.04%)	5,866 (0.05%)
Clean reads	12,558,692 (91.36%)	11,934,390 (87.15%)	11,462,346 (91.20%)	11,066,024 (92.00%)
18–30 nt sRNAs	6,063,504	5,335,626	5,377,388	5,864,215
Total bases (G)	0.687	0.685	0.628	0.601

**Table 2:** Category results of the genome mapped sRNAs of the four banana root libraries.

Types	RCK (percent)	R5H (percent)	R10 (percent)	R25 (percent)
Total	3,913,326 (100.00%)	3,422,908 (100.00%)	3,626,001 (100.00%)	3,208,726 (100.00%)
known_miRNA	57,855 (1.48%)	67,596 (1.97%)	38,680 (1.07%)	53,505 (1.67%)
rRNA	970,384 (24.80%)	762,232 (22.27%)	881,051 (24.30%)	708,078 (22.07%)
tRNA	0 (0.00%)	1 (0.00%)	0 (0.00%)	1 (0.00%)
snRNA	5,789 (0.15%)	3,942 (0.12%)	4,383 (0.12%)	4,575 (0.14%)
snoRNA	5,048 (0.13%)	5,290 (0.15%)	3,565 (0.1%)	4,215 (0.13%)
repeat	68,930 (1.76%)	65,972 (1.93%)	48,326 (1.33%)	61,630 (1.92%)
NAT	638,459 (16.31%)	586,916 (17.15%)	630,676 (17.39%)	588,303 (18.33%)
novel_miRNA	2,318 (0.06%)	2,246 (0.07%)	1,551 (0.04%)	2,740 (0.09%)
TAS	43 (0.00%)	71 (0.00%)	24 (0.00%)	55 (0.00%)
exon: +	109,172 (2.79%)	94,347 (2.76%)	91,660 (2.53%)	82,521 (2.57%)
exon: −	52,080 (1.33%)	46,865 (1.37%)	57,834 (1.59%)	52,230 (1.63%)
intron: +	35,494 (0.91%)	32,648 (0.95%)	27,082 (0.75%)	29,223 (0.91%)
intron: −	43,097 (1.10%)	41,433 (1.21%)	39,148 (1.08%)	38,194 (1.19%)
other	1,924,657 (49.18%)	1,713,349 (50.06%)	1,802,021 (49.7%)	1,583,456 (49.35%)

RCK, R5H, R10 and R25 represent the root samples treated with *Foc* TR4 for 0 h, 5 h, 10 h and 25 h, respectively.

**Figure 3:**
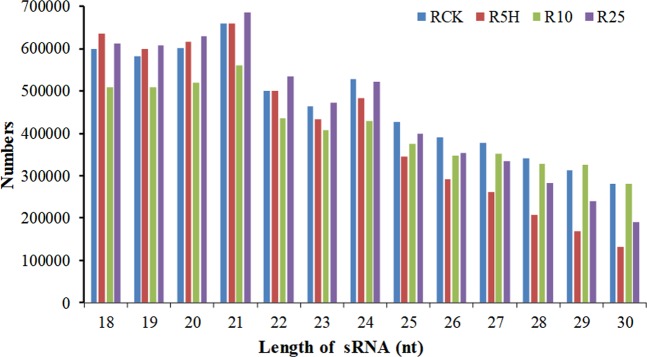
Length distribution result of the sRNAs identified in ‘Tianbaojiao’ banana root. RCK, R5H, R10 and R25 represent the root samples treated with *Foc* TR4 for 0 h, 5 h, 10 h and 25 h, respectively.

The sRNA length distribution results showed that more than 75% of the sRNAs were in the range of 18–25 nt and the 21 nt miRNAs take the largest part, followed by the 20 nt sRNAs (Fig. 3). This result was similar to the sRNAs of the 1-MCP treated banana fruit but differed a lot from the control and ethylene treated banana fruit^22^ (Bi *et al*. 2015), and also differed from the miRNA results from banana leaf  ^27^, suggesting that the sRNA populations are different in different organs of the same plants and in the same organ under different treatments^22^. Our result was also different from many other plants such as Arabidopsis, rice, maize, tomato and citrus, for which the 24 nt sRNA were the most abundant. It can be concluded that the miRNA length distribution differed in different plants^36–39^.

### Known and novel miRNAs identified in banana roots

**Figure 4:**
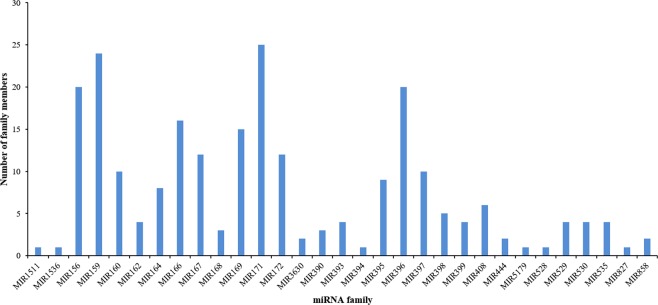
The known miRNA families and their family member numbers identified in ‘Tianbaojiao’ banana root.

For the identification of the known miRNAs, the genome mapped reads were searched against the mature miRNAs from miRBase database 21. Totally, 254 known miRNAs were identified (185, 212, 190 and 213 in RCK, R5H, R10 and R25 group, respectively), which belonged to 31 known miRNA families (Fig. 4 and Supplemental Data S2). MiR171 was the largest represented family containing 25 miRNA members, followed by miR159 family containing 24 miRNA members, and miR156, miR396 family also contained 20 miRNA members. The member numbers of miR160, miR166, miR167, miR169 and miR172 families were all more than 10. There were 9 members in miR395 family and 8 members in miR164. The member number of miR408, miR398, miR162, miR393, miR399, miR529, miR530, miR535, miR168, miR390, miR3630, miR444 and miR858 ranged from 2 to 6. While, other miRNA families had only 1 member.

The sRNA sequences that could be perfectly mapped to the banana genome but did not have a match in the miRBase were further subjected to novel miRNA prediction based on the plant miRNA annotation criteria^17^. In total, 28 novel miRNAs (22, 26, 27 and 23 for RCK, R5H, R10 and R25, respectively) were identified (Supplemental Data S3).

### Identification of the differentially expressed miRNA (DE miRNAs)

After miRNA read number normalization, Pearson correlation analysis was performed to compare the miRNA expression correlation among the four sRNA libraries. Results showed that the R^2^ coefficient among all the four samples was all more than 0.8, indicating that no significant miRNA expression change was caused in the early *Foc* TR4 infection period. Notably, R^2^ coefficient between the *Foc* TR4 treated samples (R5H, R10 and R25) and the control (RCK) decreased with the increase of the *Foc* TR4 treatment time, implying that the influence of the pathogen on banana root miRNA expression became more and more obvious as treatment time went on (Supplemental Data S4).

**Figure 5:**
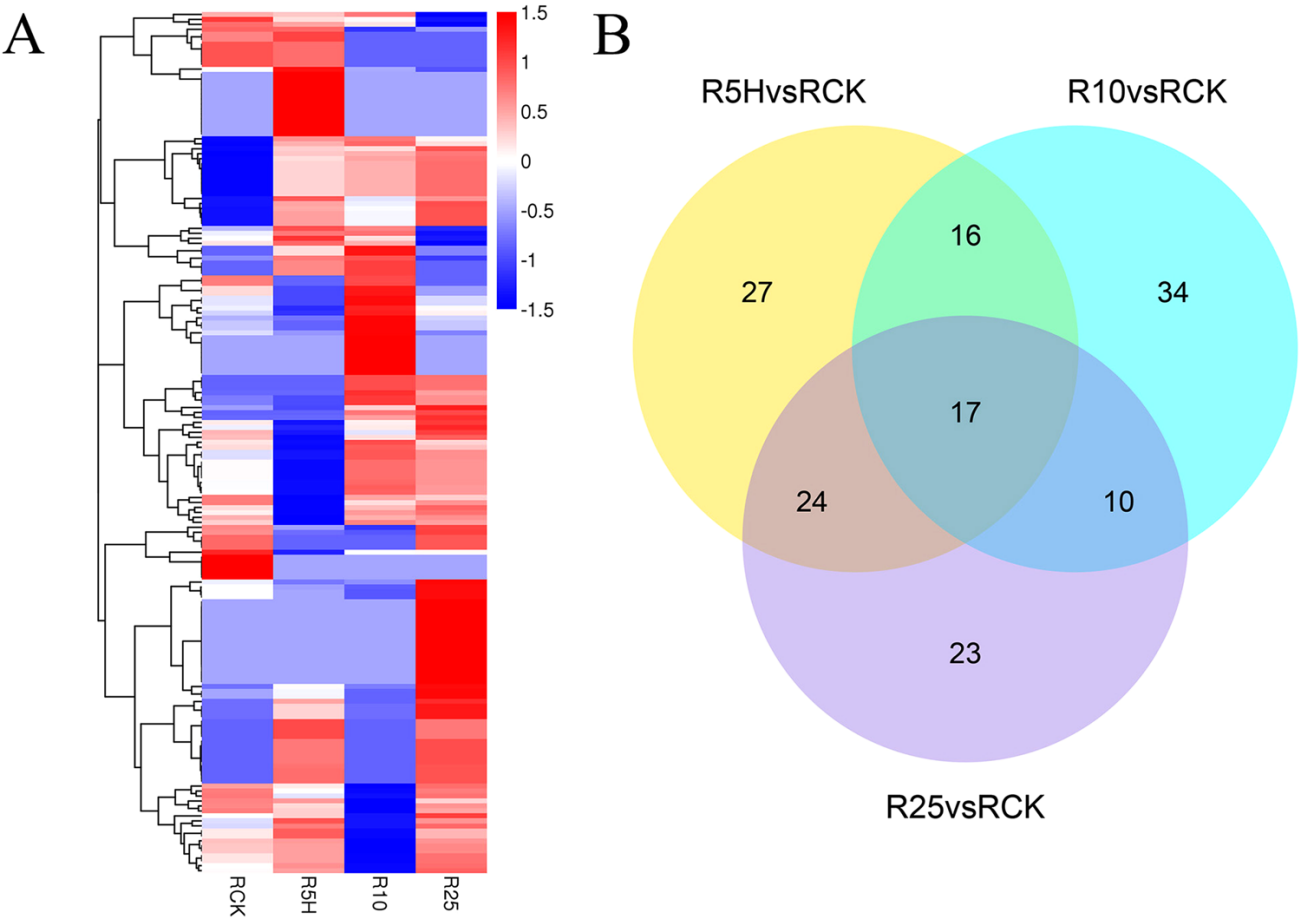
Cluster analysis and Venn diagram of the identified DE miRNAs in ‘Tianbaojiao’ banana roots. RCK, R5H, R10 and R25 represent the root samples treated with *Foc* TR4 for 0 h, 5 h, 10 h and 25 h, respectively. (**A**) Heatmap for the expression of the identified DE miRNAs in the four banana root libraries clustered by log_10_ (TPM + 1) value. The column represents different samples, and rows represent different DE miRNAs. Red color and blue color respectively means high and low expression. (**B**) Venn diagram of the DE miRNAs identified in the comparisons of R5H vs RCK, R10 vs RCK and R25 vs RCK.

Totally, 151 DE miRNAs, including 134 known miRNAs and 17 novel miRNAs, were identified by comparing the *Foc* TR4 treated groups (R5H, R10 and R25) with the control group (RCK) (Fig. 5). The DE miRNA number identified for R5H vs RCK, R10 vs RCK and R25 vs RCK was 84 (60 up-regulated, 24 down-regulated), 77 (42 up-regulated, 35 down-regulated) and 74 (69 up-regulated, 5 down-regulated) respectively. Seventeen DE miRNAs, including mac-miR156a.1, mac-miR167.1, mac-miR397a.1, mac-miR397a.2, mac-miR845, mac-miR164a.1, mac-miR397a.3, mac-miR397b-3p, mac-miR397.1, mac-nmiR13, mac-miR397.2, mac-miR160a-3p, mac-miR160d-3p, mac-miR164e, mac-miR397b.1, mac-miR397b.2 and mac-miR397.4, were identified in the three comparisons (Supplemental Data S5).

### Prediction and enrichment analysis of the DE miRNAs target genes

MiRNAs are functionally divergent, and the way they exert their critical regulatory roles is mainly through interacting specifically with their target mRNAs to suppress their activities^17,18^. So, for the function analysis of miRNA, the clarification of their target gene’s or genes’ function was very necessary. To further clarify the functions of the miRNAs, the putative target genes of these identified 254 known and 28 novel miRNAs were predicted, among which 255 miRNAs (250 known miRNAs and 5 novel miRNAs) were found to have predicted target genes. No target genes was identified for mac-miR167d.1, mac-miR8577, mac-miR1511-3p and mac-miR6478. For novel miRNAs, putative target genes only identified for mac-nmR3, mac-nmR5, mac-nmR8, mac-nmR21 and mac-nmR22.

Among the 151 DE miRNA, 1,012 putative target genes were identified for 137 DE miRNAs. The target gene number varied a lot for different DE miRNAs. The target of mac-miR5658 was found to be the most (130 predicted target genes including genes encoding MYB, RAP2, glucan endo-1,3-beta-glucosidase 3-like, aquaporins, syntaxins, WAT1-related proteins, regulatory protein NPR1-like, respiratory burst oxidase homolog protein H and so on, most of which were reported to be stress responsive), followed by mac-miR156h, mac-miR156a.1, mac-miR172c-3p, mac-miR156k, mac-miR172e-3p.1, whose target genes number was respectively 80, 79, 75, 64 and 62. The high abundance of predicted target genes suggests that these miRNAs may exert their roles through wide ways. While, only one target gene was identified for mac-miR3630-3p.1, mac-nmR3, mac-nmR8, mac-miR5538, mac-miR5141 and mac-miR3630-3p.2. Additionally, no predicted target genes was found for mac-miR8577 and some novel miRNAs (mac-nmR10, mac-nmR11, mac-nmR12, mac-nmR13, mac-nmR16, mac-nmR17, mac-nmR2, mac-nmR24, mac-nmR26, mac-nmR30, mac-nmR31, mac-nmR33 and mac-nmR9), which would make it difficult to understand their roles.

According to the target gene annotation result, the target genes of some miRNA families were found to be similar in different plant species, such as *squamosa promoter-binding-like proteins* (*SPB*) for miR156 and miR529^40^, *ARF* for miR160 and miR167^41^, *nuclear TFY* for miR169^42^, *laccase* for miR397^43^, *MYB* for miR858 and miR159^22,44^, *scarerrow* for miR171^45^, *NAC* for miR164^46^, *AP2* for miR172^47^, *EIN3* for miR395^22^. Moreover, *IAA* genes were found to be targeted by miR156, *GDSL* genes were predicted to be targets of miR164. *PPO* genes were predicted to be targeted by miR528. *Laccase* genes were also identified as targets of miR408 and miR397.

We also found that the number of the miRNA targeting the same gene also differed a lot (Supplemental Data S5), and the targeting miRNAs of the same gene are usually from the same miRNA family. For example, a *transcription factor GAMYB-like* gene (GSMUA_Achr10G29280_001) was predicted to be targets of 16 miR159s and 6 miR319s, which all belong to the miR159 family. A *scarecrow-like protein 27* (GSMUA_Achr11G04360_001) was predicted to be targets of 22 miRNA members of miR171 family. The miRNAs of the same miRNA family targeting the same gene verified the function conservation of a miRNA family. The miRNAs targeting the same gene were even found to belong to different miRNA family. For example, the gene encoding scarecrow-like protein 15 (GSMUA_Achr4G07190_001) was predicted to be targeted by 21 miR171 family members and 5 miR408 members, the *squamosa promoter-binding-like protein 12* gene (GSMUA_Achr10G02970_001) was predicted to be target of 15 miR156 family members, three miR529 family members and novel_22. Another *squamosa promoter-binding-like protein 12* gene (GSMUA_Achr10G23280_001) was predicted target of 15 miR156 family members and novel_22. And a *homeobox-leucine zipper protein HOX9* gene (GSMUA_Achr5G18530_001) was predicted to be target of 4 miRNAs of miR172 family and 13 members of miR166 miRNA family. The high diversity of the gene’s corresponding targeting miRNA suggested that the regulation of gene’s expression can be achieved by many miRNAs.

**Figure 6:**
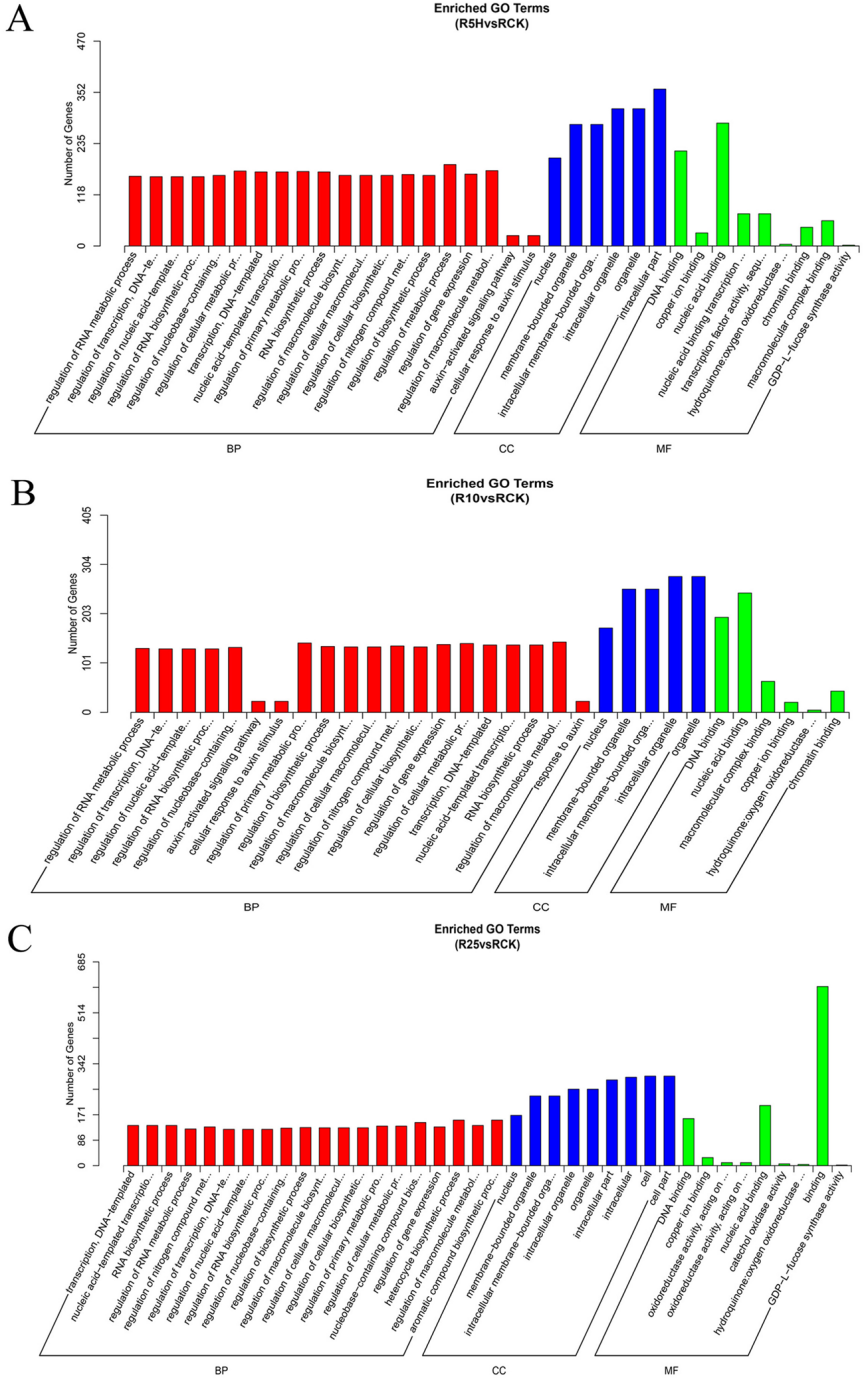
GO enrichment analysis results of the predicted target genes of DE miRNAs identified in the comparisons of R5H vs RCK (**A**) R10 vs RCK (**B**) and R25 vs RCK. (**C**) RCK, R5H, R10 and R25 represent the root samples treated with *Foc* TR4 for 0 h, 5 h, 10 h and 25 h, respectively. BP: biological process; CC: cellular component; MF: molecular function.

The predicted target genes of the DE miRNAs (DET) were then subjected to GO and KEGG analysis for their function. After GO enrichment analysis, most of the DETs were assigned to three GO ontologies, i.e. the biological process (BP), the cellular component (CC) and molecular function (MF) (Fig. 6).

From the aspect of BP, 53, 53 and 56 GO terms were significantly enriched in comparison of R5H vs RCK, R10 vs RCK and R25 vs RCK, respectively. Almost half of the enriched GO BP terms were biological process regulation related GOs such as ‘regulation of transcription’, ‘regulation of RNA biosynthetic process’, ‘regulation of RNA metabolic process’ and so on. Many other GOs such as ‘auxin-activated signaling pathway’, ‘cellular response to auxin stimulus’, ‘response to auxin’, ‘response to hormone’, ‘response to endogenous stimulus’, ‘phenylpropanoid catabolic process’, ‘lignin catabolic process’, ‘lignin metabolic process’ were also included. Notably, ‘auxin-activated signaling pathway’, ‘cellular response to auxin stimulus’ and ‘response to auxin’ GO terms were significantly enriched in all the three comparisons, and the ‘auxin-activated signaling pathway’ and the ‘cellular response to auxin stimulus’ term were even identified as the top two enriched GO terms in comparisons of R10 vs RCK and R25 vs RCK (Supplemental Data S6). The auxin signaling related genes included 17 *auxin response factor genes* (*ARFs*) targeted by members of miR156, miR160 and miR167. Moreover, ‘lignin catabolic process’ and ‘lignin metabolic process’ terms were also enriched in all the three comparisons. And the target genes enriched in the two GO terms were 4 *laccase* genes targeted by several miR408 and miR397 miRNA family members (Table 3). In addition, 4 *polyphenol oxidase* (*PPO*) genes targeted by mac-miR528-5p were found to be enriched in the ‘phenylpropanoid catabolic process’ (Table 3).

**Table 3:** The significantly enriched GO terms of the predicted target genes of DE miRNAs.

GO terms/gene IDs	Gene description	DE miRNA
**Auxin-activated signaling pathway**		
GSMUA_Achr5G18540_001	auxin response factor 18-like isoform X1	mac-miR160b.2^a,b^,mac-miR160h^a,c^
GSMUA_Achr10G26310_001	auxin-responsive protein IAA17-like	mac-miR156a.1^a,b,c^,mac-mir156h^b^
GSMUA_Achr11G18520_001	auxin response factor 17-like isoform X1	mac-miR167.1^a,c^,mac-miR167c-5p^a,c^, mac-miR167.1^b,*^
GSMUA_Achr6G19850_001	auxin response factor 17-like	mac-miR167.1^a,c^,mac-miR167c-5p^a,c^, mac-miR167.1^b,*^
GSMUA_Achr8G13620_001	auxin response factor 12-like	mac-miR167.1^a,c^,mac-miR167c-5p^a,c^, mac-miR167.1^b,*^
GSMUA_Achr4G25960_001	auxin response factor 12-like isoform X1	mac-miR167.1^a,c^,mac-miR167c-5p^a,c^ mac-miR167.1^b,*^
GSMUA_Achr3G23290_001	auxin response factor 17-like	mac-miR167.1^a,c^,mac-miR167c-5p^a,c^ mac-miR167.1^b,*^
GSMUA_Achr4G18240_001	auxin response factor 18-like	mac-miR160b.2^a,b^,mac-miR160h^a,c^
GSMUA_AchrUn_randomG06470_001	auxin response factor 12-like isoform X2	mac-miR167.1^a,c^,mac-miR167c-5p^a,c^ mac-miR167.1^b,*^
GSMUA_Achr11G25770_001	auxin response factor 6-like	mac-miR167.1^a,c^,mac-miR167c-5p^a,c^ mac-miR167.1^b,*^
GSMUA_Achr10G04600_001	auxin response factor 17-like isoform X1	mac-miR160b.2^a,b^,mac-miR160h^a,c^
GSMUA_Achr8G18930_001	auxin response factor 18-like isoform X1	mac-miR160b.2^a,b^,mac-miR160h^a,c^
GSMUA_Achr5G00590_001	auxin response factor 17-like	mac-miR167.1^a,c^,mac-miR167c-5p^a,c^ mac-miR167.1^b,*^
GSMUA_Achr5G14630_001	auxin response factor 18 isoform X2	mac-miR160b.2^a,b^,mac-miR160h^a,c^
GSMUA_Achr5G26580_001	auxin response factor 6-like isoform X1	mac-miR167.1^a,c^,mac-miR167c-5p^a,c^ mac-miR167.1^b,*^
GSMUA_Achr11G01610_001	auxin response factor 12-like	mac-miR167.1^a,c^,mac-miR167c-5p^a,c^ mac-miR167.1^b,*^
GSMUA_Achr9G29480_001	auxin response factor 17-like isoform X1	mac-miR160b.2^a,b^,mac-miR160h^a,c^
GSMUA_Achr5G02450_001	auxin response factor 6-like	mac-miR167.1^a,c^,mac-miR167c-5p^a,c^ mac-miR167.1^b,*^
GSMUA_Achr5G03960_001	auxin response factor 18-like	mac-miR160b.2^a,b^,mac-miR160h^a,c^
**Lignin metabolic process/Phenylpropanoid catabolic process**		
GSMUA_Achr3G09960_001	laccase-3-like	mac-miR408d^a,c^,mac-miR408-3p.3^a,c^,mac-miR397.1^a,b,c^,mac-miR397.4^a,b,c^
GSMUA_Achr6G05000_001	laccase-4-like	mac-miR397a.1^a,b,c^,mac-miR397.4^a,b,c^
GSMUA_Achr3G15320_001	laccase-11	mac-miR397.2^a,b,c^,mac-miR397b.1^a,b,c^,mac-miR397b.2^a,b,c^,mac-miR397.4^a,b,c^,mac-miR397a.1^a,b,c^,mac-miR397a.2^a,b,c^,mac-miR397a.3^a,b.c^,mac-miR397.1^a,b,c^,mac-miR397.3^a^
GSMUA_Achr9G03350_001	laccase-4-like	mac-miR397.4^a,b,c^,mac-miR397b.2^a,b,c^,mac-miR397.2^a,b,c^,mac-miR397b.1^a,b,c^,mac-miR397.1^a,b,c^,mac-miR397.3^a^,mac-miR397a.2^a,b,c^,mac-miR397a.1^a,b,c^,mac-miR397a.3^a,b.c^
**Catechol oxidase activity**		
GSMUA_Achr8G34370_001	polyphenol oxidase	mac-miR528-5p^c^
GSMUA_Achr6G29370_001	polyphenol oxidase	mac-miR528-5p^c^
GSMUA_AchrUn_randomG22740_001	polyphenol oxidase	mac-miR528-5p^c^
GSMUA_Achr7G03450_001	polyphenol oxidase	mac-miR528-5p^c^
GSMUA_AchrUn_randomG25220_001	polyphenol oxidase	mac-miR528-5p^c^
GSMUA_AchrUn_randomG22730_001	polyphenol oxidase	mac-miR528-5p^c^

^a^R5H vs RCK, ^b^R10 vs RCK, ^c^R25 vs RCK, the miRNA with/without *means that it was down-regulated/up-regulated.

From the aspect of CC, 10 terms, including ‘nucleus’, ‘membrane-bounded organelle’, ‘intracellular membrane-bounded organelle’, ‘intracellular organelle’, ‘organelle’, ‘intracellular part’, ‘intracellular’, ‘cell’, ‘cell part’, ‘cellular component’ were enriched for all the three comparisons.

From the aspect of MF, 10, 12 and 9 terms were significantly enriched respectively for the three comparisons. Terms such as ‘DNA binding’, ‘nucleic acid binding’, ‘copper ion binding’, ‘hydroquinone: oxygen oxidoreductase activity’, ‘binding’, ‘GDP-L-fucose synthase activity’, ‘oxidoreductase activity, acting on diphenols and related substances as donors, oxygen as acceptor’, ‘DNA binding’, ‘nucleic acid binding’, ‘copper ion binding’ were enriched at least 2 comparisons. Terms of ‘nucleic acid binding transcription factor activity’, ‘transcription factor activity, sequence-specific DNA binding’ and ‘protein dimerization activity’ were only enriched in comparison of R5H vs RCK. Terms of ‘heterocyclic compound binding’, ‘organic cyclic compound binding’, ‘macromolecular complex binding’, ‘chromatin binding’, ‘sulfate adenylyltransferase (ATP) activity’ and ‘sulfate adenylyltransferase activity’ were only enriched in comparison of R10 vs RCK, and ‘oxidoreductase activity, acting on diphenols and related substances as donors’ and ‘catechol oxidase activity’ terms were found to be specifically enriched in comparison of R25 vs RCK.

**Figure 7:**
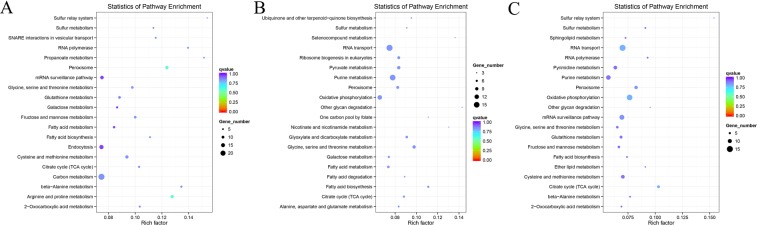
KEGG enrichment analysis results of the predicted target genes of DE miRNAs identified in the comparisons of R5H vs RCK (**A**) R10 vs RCK (**B**) and R25 vs RCK. (**C**) RCK, R5H, R10 and R25 represent the root samples treated with *Foc* TR4 for 0 h, 5 h, 10 h and 25 h, respectively. Only the top 20 enriched pathways of each comparison were shown.

KEGG pathway enrichment analysis showed that no significant pathway enrichment was identified, which may be due to the sample used being in the early *Foc* TR4 infection stage thereby causing no significant change. The top 20 pathways influenced by the *Foc* TR4 in the three early stages were shown in Fig. 7 and Supplemental Data S7. DETs involved in alanine, aspartate and glutamate metabolism, selenocompound metabolism, SNARE interactions in vesicular transport and some other 19 pathways were only enriched in one comparison. Cysteine and methionine metabolism, fatty acid metabolism, mRNA surveillance pathway, phenylalanine, tyrosine and tryptophan biosynthesis, protein export, purine metabolism, RNA transport pathways were found among the top 20 enriched pathways of two comparisons. Notably, pathways of peroxisome, fatty acid biosynthesis, fatty acid degradation, sulfur metabolism, nicotinate and nicotinamide metabolism, pyrimidine metabolism, pyruvate metabolism and metabolic pathways were found among the top 20 enriched pathways of all the three comparisons (Supplemental Datas S8 and S9), suggesting that the *Foc* TR4 greatly influenced these pathways in the early infection stages.

### Quantitative analysis for the expression of miRNAs and their target genes

**Figure 8:**
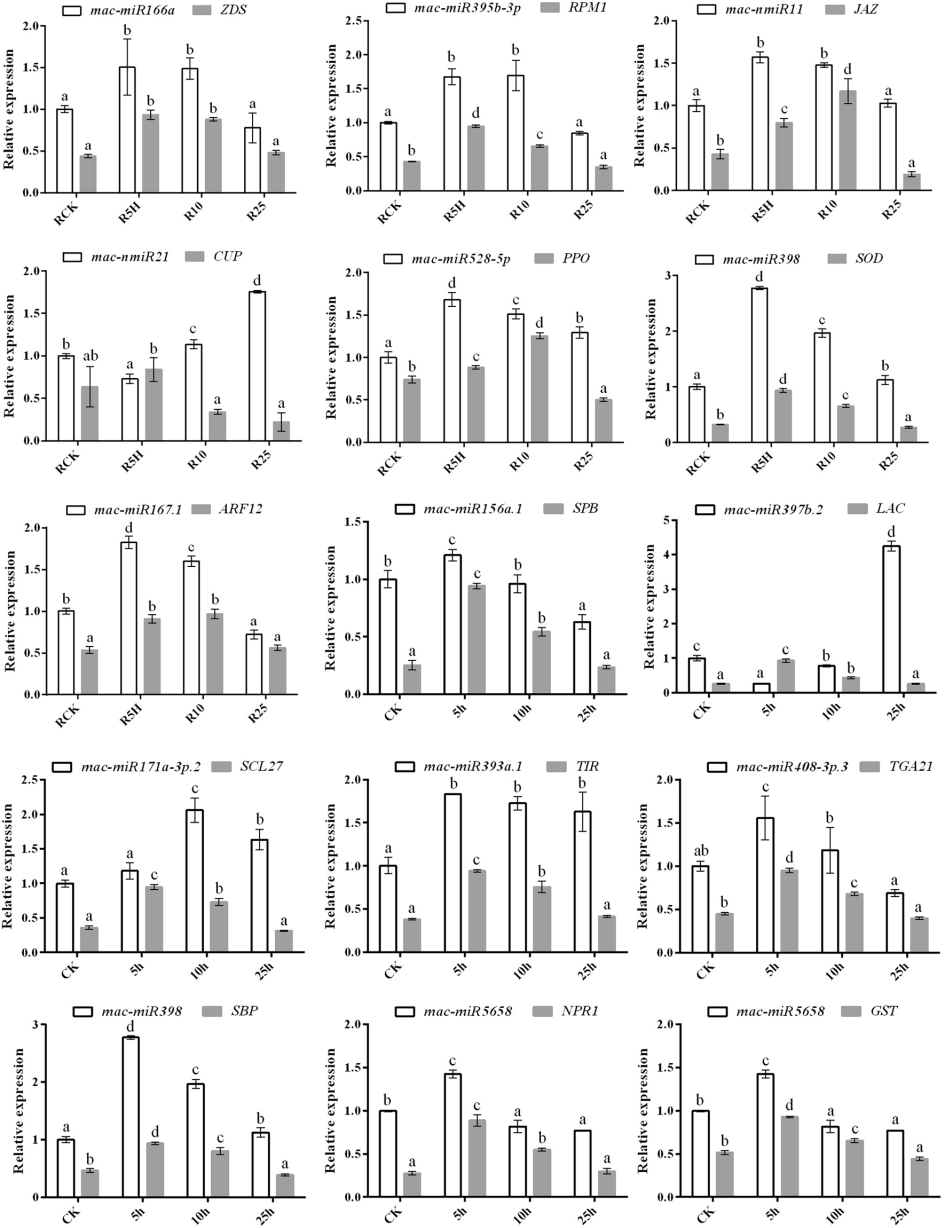
Quantitative real time PCR (qRT-PCR) result of the selected miRNAs and their corresponding target genes. Error bars represent the standard deviation. *ZDS*: *Zeta*-*carotene desaturase*; *RPM1*: *disease resistance protein RPM1*; *JAZ*: *Jasmonate ZIM*-*domain protein*; *CUC2*: *protein CUP*-*SHAPED COTYLEDON 2*; *PPO*: *polyphenol oxidase*; *SCL27*: *scarecrow like protein 27*; *TGA21*: *transcription factor TGA21*; *SBP*: *selenium*-*binding protein*; *SOD*: *superoxide dismutase* [*Cu*-*Zn*]; *SPB16*: *putative squamosa promoter*-*binding*-*like protein 16*; *LAC*: *laccase*-*25*; *NPR1*: *putative regulatory protein NPR1*; *GST*: *glutathione S*-*transferase zeta class*; *TIR*: *transport inhibitor response 1*-*like protein*; *ARF12*: *auxin response factor 12*. Different letters on the columns of the same color indicate significant difference at p-value < 0.05.

The expression of 11 known miRNA and 2 novel miRNA identified in this study was analyzed using poly(A) miRNA-based qRT-PCR^33^ (Fig. 8). After *Foc* TR4 infection, although the expression of mac-miR166a was very significantly up-regulated at R5H and R10 (p-value < 0.01), their fold change were both less than 2 (Supplemental Data S2). The sequencing result also revealed it to be a not differentially expressed miRNA. The expression of mac-miR395b-3p was also slightly up-regulated at the early infection stages and a very significant change was found, although it was not identified as DE miRNA in the RNA-seq data (Supplemental Data S2). Mac-nmiR11 was identified as DE miRNA at R5H compared with RCK, and no expression was detected at R25 (Supplemental Data S2). Our qRT-PCR result showed that the miRNA’s relative expression was significant higher than RCK at R5H and R10H, and no significant difference were found between RCK and R25. Mac-nmiR21 was identified as a R25 specific novel miRNA in our sequencing result (Supplemental Data S2), and the highest relative expression of it was also found in R25 by using qRT-PCR. Mac-miR528-5p was identified as an up-regulated DE miRNA at R25 (Supplemental Data S2), and our qRT-PCR result also revealed that it was significantly up-regulated at R25. Moreover, its expression at R5H and R10 were also found to be very significantly higher than RCK (p-value < 0.01). Mac-miR171a-3p.2 was identified as a down-regulated DE miRNA at R5H and its highest expression was found at R10 in the RNA-Seq data (Supplemental Data S2). Our qRT-PCR result revealed that mac-miR171a-3p.2 expressed the highest at R10 but no significant expression change was found between R5H and RCK (p-value < 0.05). Mac-miR408-3p.3 was identified as an up-regulated DE miRNA at R5H and R25 in the RNA-Seq result (Supplemental Data S2). However, by using qRT-PCR, very significant up-regulation was only identified at R5H (p-value < 0.01) and its expression was found to be down-regulated at R25 compared with RCK. Mac-miR398 was identified as an up-regulated DE miRNA at R5H and R10 in the RNA-Seq result (Supplemental Data S2). Consistently, by using qRT-PCR, very significant expression change was also found at the same two time-point (p-value < 0.01). Mac-miR167.1 was an up-regulated at R5H and R25 but down-regulated at R10 compared with RCK according to our RNA-Seq results (Supplemental Data S2). The qRT-PCR result, however, revealed that this miRNA was significantly up-regulated at R5H and R10, but down-regulated at R25. Mac-miR156a.1 was identified as up-regulated DE miRNA at all the three *Foc* TR4 infected groups by RNA-seq (Supplemental Data S2). Its up-regulation at R5H was verified by qRT-PCR analysis, this miRNA was found to be significantly up-regulated at R5H but significantly down-regulated at R25 compared with RCK, and no significant expression difference was found between R10 and RCK. Mac-miR393a.1 was identified as up-regulated DE miRNA at R10 and R25 (Supplemental Data S2). Consistently, its up-regulation at the two time points was also verified by using qRT-PCR. Besides, its up-regulation was also found at R5H. Mac-miR397b.2 was identified as up-regulated DE miRNA at all the three *Foc* TR4 infected groups by RNA-seq (Supplemental Data S2). Although our qRT-PCR result showed it was down-regulated at R5H and its expression change at R10 compared with RCK was not significant, it was found to be up-regulated more than 4 fold at R25 compared with RCK. Mac-miR5658 was identified as a R5H specific novel miRNA in our sequencing result (Supplemental Data S2), and its expression at R5H was also found to be significant higher than RCK at R25 by using qRT-PCR. Overall, there were some consistencies between the sRNA high-throughput sequencing data and our RNA-Seq result. The differences might be caused by the calculation and principle differences of the two methods.

To investigate the expression patterns of the miRNAs’ target genes, the expression levels of one predicted target of these miRNAs were checked. Expected inverse relationship during the early *Foc* TR4 infection stage was identified between mac-nmiR21 and *CUP*, and mac-miR397b.2 and *LAC*. However, no contrasting expression pattern was found between the expression of the other miRNAs and their corresponding target genes, indicating that they may not target genes or these genes’ expression regulation were more complex^22^.

## Discussion

### Peroxisome and fatty acid metabolism were greatly influenced by *Foc* TR4 infection

Pathogen infection will induce the generation of reactive oxygen species (ROS)^48^. Peroxisomes are important producers of ROS and many antioxidants are located in them^49^. In this study, the target genes of several DE miRNA, such as mac-miR398, mac-miR398a-3p.1, mac-miR156a.1, mac-miR397.1 and so on, were involved in peroxisomes pathway. The pathway was found in the top 20 enriched pathways in all the three *Foc* TR4 infected groups compared with RCK, indicating that the *Foc* TR4 infection greatly influenced the peroxisome pathway.

Plant will induce rapid production of ROS in response to pathogen infection to inhibit their growth and invasion^11,50^. However, ROS accumulation will also cause damages to plant cells. Notably, all the DE miRNAs that own peroxisome related target genes were found to be up-regulated compared with RCK. Our qRT-PCR result also confirmed the up-regulation of miR398. Thus, it was deduced that the up-regulation of these miRNAs might be very necessary for decreasing the ROS damage to banana itself  ^50^.

Peroxisomes accomplish a variety of biochemical functions, in which β-oxidation of fatty acids were included^51,52^. Fatty acids (FAs) are an important source of reserve energy and essential components of membrane lipids in all living organisms and are also biosynthetic precursors of cuticular components and phytohormone jasmonic acid (JA). Therefore, FA metabolic pathways play significant roles in pathogen defense^53,54^. Recently, FA has been identified as the major carbon source that transfer from host plant to symbiotic fungus^55,56^. And plant FAs were proved to be required for pathogen colonization^56^. In our present study, several DE miRNAs were predicted to target FA metabolism related genes such as *biotin carboxylase 2*, *acetyl-CoA carboxylase 1* and two *long chain acyl-CoA synthetase 9* genes. It was deduced that these miRNAs contribute a lot to the banana resistance by influencing the nutrient exchange between banana and *Foc* TR4.

### Auxin pathway is an important target of *Foc* TR4 to facilitate its infection

The plant growth-defense tradeoffs regulation are dependent on the phytohormonal networks^57^. However, pathogens could disturb plant hormonal homeostasis to defeat plant immunity, modify babitate structure, enhance nutrient acquisition and facilitate infection and dissemination^57^.

Auxins are an important phytohormone for plant growth and disease resistance^58–61^. The reduction of auxin content was found to be significant for the enhancement of plant fungal pathogen resistance^62,63^. In Arabidopsis, *F*. *oxysporum* infection was reported to alter the auxin homeostasis in the root and promoted the pathogen colonization, and the auxin-signaling mutants were found to be more resistant to *F*. *oxysporum*^64^. Notably, the *Foc* 4 infection in banana could also increase the auxin content in banana^65^. All these suggested that pathogens could enhance the auxin biosynthesis of the plant to alter plant growth and development in their favor^66^. Conversely, in our study, many auxin signaling related *auxin response factor* genes (*ARFs*) were predicted to be targets of up-regulated DE miRNAs such as members of miR156, miR160 and miR167 families. This indicated that their expression was down-regulated. MiRNA-directed regulation of *ARFs* was proven to be essential for the root development and pivotal for modulating the expression of early auxin response genes^67,68^. Our qRT-PCR result also revealed that mac-miR167.1 was significantly up-regulated at R5H and R10. The up-regulation of their corresponding miRNAs suggested that *Foc* TR4 infection repressed the auxin response pathway. The suppression of the auxin response pathway could decrease the resistance of Arabidopsis to fungal pathogens^60,69^. The up-regulation of members of miR156, miR160 and miR167 families might function in banana-*Foc* TR4 interaction by repressing banana auxin response pathway.

Transport inhibitor response protein 1 (TIR1), the auxin receptor, is important for auxin signaling. In our present study, two *TIR1* genes were predicted to be target genes of up-regulated miR393 members. And mac-miR393a.1, targeting a *TIR1* gene (GSMUA_Achr5G26930_001), was identified to be significantly up-regulated in R5H, R10 and R25. MiR393 could enhance the plant disease resistance by negatively regulating genes for the F-box auxin receptors including *TIR1*^70^. In cassava, higher miR393 expression was found in more anthracnose disease resistant cultivar, suggesting that this miRNA plays a role in fungal-infected cassava by influencing the auxin signaling^71^. The up-regulation of these miR393s in *Foc* TR4 infected banana root indicated that they might act in banana disease response by repressing auxin signaling.

### Sulfate metabolism related miRNAs were *Foc* TR4 responsive

According to the KEGG pathway enrichment analysis, the sulfur metabolism were found to be enriched in all the three *Foc* TR4 infection stages, suggesting that sulfur metabolism were greatly influenced by the pathogen infection. Most of the DE miRNAs targeting sulfur metabolism related genes were up-regulated at least one stage, such as mac-miR171a.2, mac-miR171b.2 and mac-171c.1 targeting *serine acetyltransferase 1* and mac-miR395t targeting *ATP sulfurylase 1* genes. And our qRT-PCR results also verified the up-regulation of miR171 and miR395 member. The differential expression of these miRNAs might influence the sulfur metabolism in banana root. In plant, sulfur is an important component, and the sulfur nutritional status were directly related to plants’ health^72^. Moreover, sulfate metabolism was close related to the ethylene biosynthesis^73^, and *Foc* infection could significantly activate the ethylene biosynthesis and signaling related genes^13^. Additionally, accumulating evidences proved that its assimilation and metabolism are regulated by a variety of stresses^74^ and sufficient sulfate supply was found to be necessary for plant development due to sulfur induced resistance or sulfur enhanced defense^75,76^. Thus, it was concluded that miRNA-mediated sulfur metabolism changes might function in the *Foc* TR4 infection response of banana.

### MiRNA participate in the banana wilt disease response by regulating lignin metabolism

Plant secondary metabolites, such as lignin, contributed greatly to an enhanced resistance to fungal diseases^77^. In Arabidopsis, miR397 and miR408 potentially regulate several *laccase* genes^78^. MiR397 could target the two *laccase* (*lac*) genes, *LAC4* and *LAC17*, involved in lignification^79,80^. While the over-expression of miR408 in Arabidopsis changed the root gravitropic response^81^. In *Populus trichocarpa*, miR408 showed regulatory role in suppressing lignin deposition, and it was near basent in the major lignification tissue-the developing xylem^82^. MiR397 was also found to be the negative regulator of *LAC* genes affecting lignin content. In our present study, four *lac* genes were found to be targeted by the banana miR397 and miR408 family members. And our RNA-Seq result identified that the DE miRNAs targeting *LAC* genes were all significantly up-regulated by *Foc* TR4. Moreover, mac-miR397b.2 targeting *LAC* gene was found to be up-regulated more than 4 times at R25 compared with RCK according to our qRT-PCR result. It suggested that the lignin deposition in banana root was suppressed in response to *Foc* TR4 infection.

MiR528 is a monocot-specific miRNA. In this study, the *Foc* TR4 responsive mac-miR528-5p was predicted to target 4 *PPO* genes. PPO function a lot in cell wall lignification. In the study of Deng^15^, the cell wall lignification related genes were significantly induced by *Foc* TR4 in Fusarium wilt resistant ‘Kangku No. 5′, indicating that these genes might contribute to its high resistance. In maize, miR528 was predicted to target *lac* genes and its negative regulation role on *lac* expression and lignin biosynthesis were also identified^83^. The up-regulation of mac-miR528-5p in *Foc* TR4 infected banana root indicated that *Foc* TR4 infection might influence the banana cell wall lignification. The up-regulation of miR397, miR408 and miR528 in banana root suggested that banana altered its lignin metabolism in response to *Foc* TR4 infection.

### Mac-miR5658 was a stress related miRNA

*NPR1*, one predicted target of miR5658, is one of the best-studied resistance-related genes^84^. *Aquaporins*, predicted target genes of mac-miR5658, are important for root development and their expression was regulated by auxin^6,85^, which again could provide evidence for the role of auxin in banana-*Foc* TR4 interaction. Coincidentally, the gene encoding WAT1, a vacuolar auxin transport facilitator required for auxin homeostasis^86^, was also predicted to be target of miR5658. Moreover, SNARE interactions in vesicular transport was identified to be enriched at R5H. The genes involved in this pathways were *syntaxins* that targeted by mac-miR5658. S*yntaxins* have been function in mediating the root growth^49^, and they also contribute to the plant disease resistance especially to the fungal diseases^87–92^. Mac-miR5658 owns so many stress responsive or pathogenesis related target genes, and its significant up-regulation revealed by both RNA-Seq and qRT-PCR indicated that it may contributed a lot during the banana-*Foc* TR4 interaction. The function of this miRNA need to be further studied for the clarification of its role in banana disease resistance.

**Figure 9:**
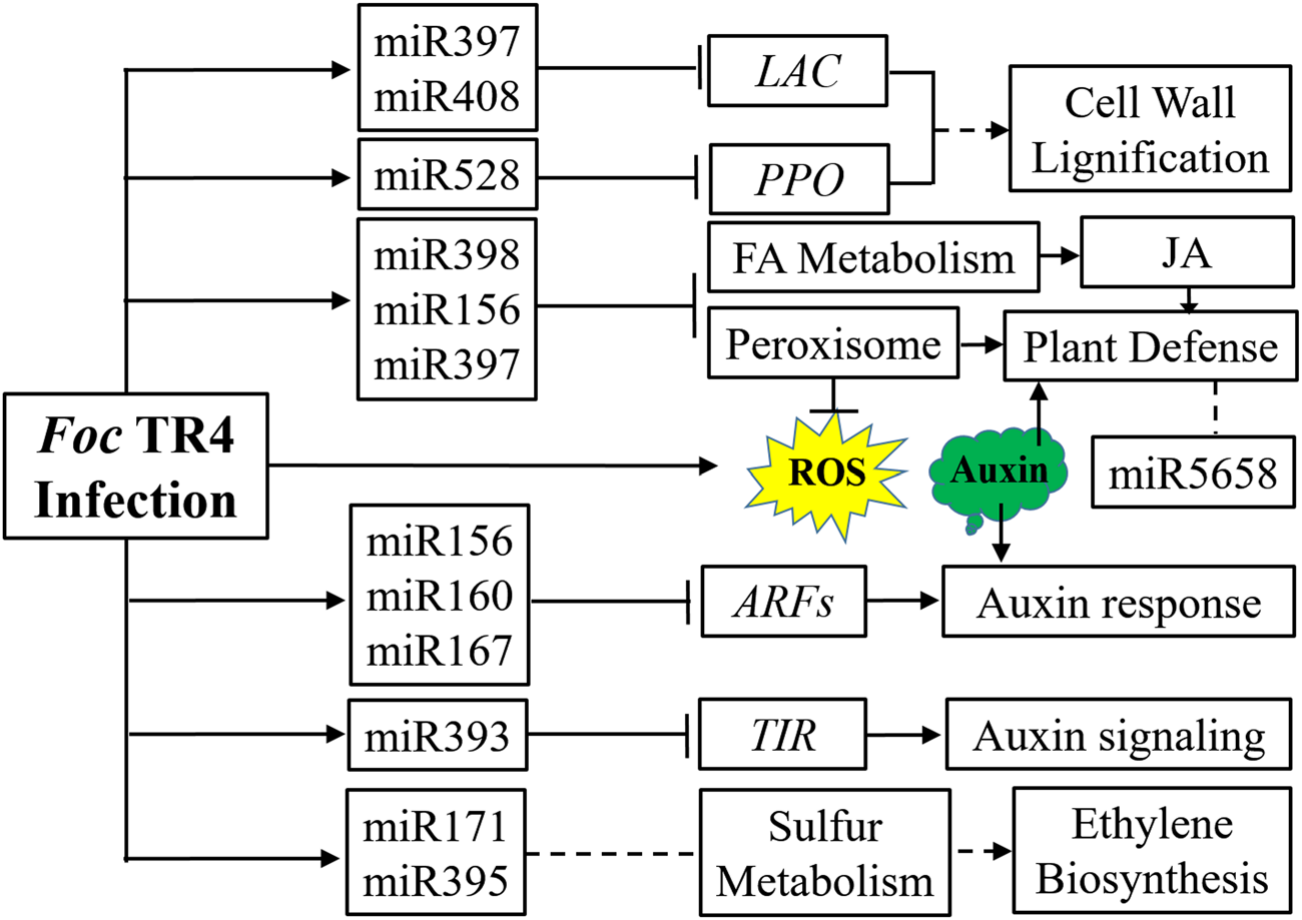
The role of miRNAs in the complex defense response to the *Foc* TR4 infection. Cell wall is the first barrier to the pathogen^48^, at the early stages of the *Foc* TR4 infection, several miRNAs including miR397, miR408 and miR528 were induced, which will lead to the down-regulation of *LAC* and *PPO* genes and ultimately the suppression of the cell wall lignification^78–82^. The *Foc* TR4 infection will induce the accumulation of ROS^48^, which will also cause damage to the plant cell^11,50^. The expression of banana miR156, miR397 and miR398 was induced, which will repress the expression of peroxisome related and FA metabolism related genes. The repression of peroxisome related genes will alleviate the harmful effect of ROS, while the FA metabolism change will influence the nutrient exchange between banana roots and *Foc* TR4 and the metabolism of JA^51–54^. The auxin content in banana could be enhanced by the *Foc* TR4 infection^65^, which might be helpful for the infection of pathogen^60,69^. To suppress the auxin signaling, miR156, miR160, miR167 and miR393 were up-regulated. The up-regulation of miR171 and miR395 will influence the sulfur metabolism and the ethylene biosynthesis^72,73^, which might contribute to the banana defense to *Foc* TR4. Mac-miR5658 was predicted to target many stress or pathogen responsive genes, indicating that it plays a role in regulation of banana-*Foc* TR4 interactions.

MiRNA plays important roles in plant-fungal pathogen interaction by regulating the plant genes’ expression or by silencing the virulence genes of fungal pathogens^93^. In this study, we identified 254 known miRNAs from 48 miRNA families and 28 novel miRNAs from roots of *Foc* TR4-inoculated ‘Tianbaojiao’ banana in the early stage (i.e. 5 h, 10 h and 25 h post *Foc* TR4 inoculation, respectively), which could provide basis for the exploration of the banana disease response and the pathogenesis of *Foc* TR4. Differential expression analysis identified 84, 77 and 74 differentially expressed (DE) miRNAs at the three *Foc* TR4 infection times respectively compared with control healthy banana (CK). Function analysis of the DETs revealed the role of banana miRNAs in the complex defense response to *Foc* TR4 infection mainly by regulating the expression of peroxisome, fatty acid metabolism, auxin-activated signaling pathway, sulfur metabolism and so on, which will lead to the cell wall lignification repression, fungi growth inhibition, phytohormone signaling and plant defense activation (Fig. 9).

## Supplementary information

Supplemental information

## Data Availability

All the data generated or analyzed during this study are included in this published article and its Supplementary Information Files.
