# Supplementary material for: Identification of Fusarium oxysporum f. sp. cubense tropical race 4 (Foc TR4) responsive miRNAs in banana root
Source: Sci Rep. 2019 Sep 23;9:13682. doi: 10.1038/s41598-019-50130-2 (PMC6757108; doi:10.1038/s41598-019-50130-2)
Supplement: Supplementary file 1 — Supplemental information [file 41598_2019_50130_MOESM1_ESM.pdf]

Identification of *Fusarium oxysporum* f. sp. *cubense* tropical race 4 (*Foc* TR4) responsive miRNAs in banana root

Chunzhen Cheng*, Fan Liu, Xueli Sun, Na Tian, Raphael Anue Mensah, Dan Li, Zhongxiong Lai*

Supplemental data S1 Primers used in this study

miRNA or Gene Name	Sequence or Gene ID	Forward Primer	Reverse Primer	Annealing Temperature
mac-miR166a	UCGGACCAGGCUUCAUUCC	TCGGACCAGGCTTCATTCC	GATCGCCCTTCTACGTCGTAT	60℃
mac-miR395b-3p	AAGUGUUUGGGGGAACUC	AAGTGTTTGGGGGAACTC	GATCGCCCTTCTACGTCGTAT	54℃
mac-nmiR11	UAGGAGAGAUGACACCGGCUU	TAGGAGAGATGACACCGGCTT	GATCGCCCTTCTACGTCGTAT	60℃
mac-nmiR21	UGGAGAAGCAGGGCACGUGCAC	TGGAGAAGCAGGGCACGTGCAC	GATCGCCCTTCTACGTCGTAT	55℃
mac-miR528-5p	UGGAAGGGGCAUGCAGAGGA[illegible]	TGGAAGGGGCATGCAGAGGAG	GATCGCCCTTCTACGTCGTAT	60℃
mac-miR171a-3p.2	UGAUUGAGCCGCGCCAAUAU	TGATTGAGCCGCGCCAATAT	GATCGCCCTTCTACGTCGTAT	55℃
mac-miR408-3p.3	CUGCACUGCCUCUUCCCUGGC	CTGCACTGCCTCTTCCCTGGCA	GATCGCCCTTCTACGTCGTAT	60℃
mac-miR398	UGUGUUCUCAGGUCACCCCU	TGTGTTCTCAGGTCACCCCTA	GATCGCCCTTCTACGTCGTAT	57℃
mac-miR167.1	UCAAGCUGCCAGCAUGAUCUA	TCAAGCTGCCAGCATGATCTA	GATCGCCCTTCTACGTCGTAT	55℃
mac-miR156a.1	UGACAGAAGAGAGAGAGCAC	TGACAGAAGAGAGAGAGCAC	GATCGCCCTTCTACGTCGTAT	55℃
mac-miR397b.2	UUUGAGUGCAGCGUUGAUGA	TTTGAGTGCAGCGTTGATGA	GATCGCCCTTCTACGTCGTAT	54℃
mac-miR393a.1	UCCAAAGGGAUCGCAUUGAUC	TCCAAAGGGATCGCATTGATC	GATCGCCCTTCTACGTCGTAT	55℃
mac-miR5658	AUGAUGAUGAUGAUGAUGAAA	ATGATGATGATGATGATGAAA	GATCGCCCTTCTACGTCGTAT	54℃
U6	CAUCCGAUAAAAUUGGAACGA	CATCCGATAAAATTGGAACGA	GATCGCCCTTCTACGTCGTAT	54℃
ZDS	GSMUA_Achr4T17130_001	CGGGGAAGAATTAGCAGAGC	GCTTGCCAGAGAGAGGTAGA	58℃
RPM1	GSMUA_Achr8T30710_001	ACGTGCTCTTCCCGATAACA	CCACCGTCTCCAAGTGTTTG	58℃
JAZ	GSMUA_Achr5T05900_001	AGGATTCGAGAGCTGCAACT	CCTCCCTTCAGAAGAGACGA	58℃
CUC2	GSMUA_Achr10T22350_001	TTCTCGGCAGGTCTTAAGGC	AGCTGGAGATTCTCGTGGAG	58℃
PPO	GSMUA_Achr7T03450_001	TATTCAGCTCCTCACCCTGC	TCGACCTTAGCAGCGATCTC	58℃
SCL27	GSMUA_Achr8T11160_001	AATCACCAACTCCCCTCTCC	TGTGCACAACGTCCAAATGA	58℃
TGA21	GSMUA_Achr2T06770_001	CAGGGTCTCTTCTTGGGAGG	CTGGAAGAGCTCGTCGTAGA	58℃
SBP	GSMUA_Achr6T36110_001	AAGTTGAGGCTTCGATCCGG	TCGCCAAGTAGTCCGGTTTT	58℃
ARF12	GSMUA_Achr11T01610_001	ATCAGTGGGGAGGTCTTTGG	TCCAAGGAGAAGCACATCGT	58℃
SPB16	GSMUA_AchrUn_randomT21900_001	AGCAGAAAGTAGCAGCAGGA	ACTGGAGGCTCGAAACAAGA	58℃
LAC	GSMUA_Achr6T33870_001	GTCTCCGATGCATTCACCAT	CCGGCAACCTTGAAGAAGAA	58℃
TIR1	GSMUA_Achr3T23850_001	AATGCTGCGAAACTGGAGAC	TGGGGTGTCAGATCTTGGAC	58℃
SOD	GSMUA_Achr2T02380_001	TCGTCTTCTCGTTTCGGAGG	AAGGCCAGAGATGGATCCAG	58℃
NPR1	GSMUA_Achr7T09190_001	CAGAACAGTGGAACTCGGGA	AATGCCGACCGATGTTGATG	58℃
GST	GSMUA_Achr8T29090_001	TTAACCAATACAACGGCCGG	CAGTCGGAATACAGCGTGAG	58℃
EIF5A-2	FJ800963.1	ACACCAAAGATGATCTGAGGCT	AAGGAACACCAGGGCAACAGT	58℃
GAPDH	AY821550	GCAAGGATGCCCCAATGT	AGCAAGACAGTTGGTTGTGCAG	58℃

Supplemental data S2 Information of the identified known miRNAs

MiRNA name	Homology miRNA	Mature miRNA sequence	RCK (TPM)	R5H (TPM)	R10 (TPM)	R25 (TPM)	Fold change (R5H/RCK)	Fold change (R10/RCK)	Fold change (R25/RCK)	Signature (R5H/RCK)	Signature (R10/RCK)	Signature (R25/RCK)
mac-miR1511-3p	ppe-miR1511-3p	ACCUGGCUCUGAUACCAUAAC	43.44	21.06	79.72	26.60	-1.05	0.88	-0.71	FALSE	FALSE	FALSE
mac-miR156	hbr-miR156	UUGACAGAAGAUAGAGAGC	304.10	221.09	239.16	399.05	-0.46	-0.35	0.39	FALSE	FALSE	FALSE
mac-miR156a.1	ahy-miR156a	UGACAGAAGAGAGAGAGCAC	0.00	10.53	8.86	26.60	4.40	4.15	5.73	TRUE	TRUE	TRUE
mac-miR156a.2	pta-miR156a	CAGAAGAUAGAGAGCACAUC	6.21	0.00	0.00	0.00	-3.63	-3.63	-3.63	TRUE	TRUE	FALSE
mac-miR156a-3p	zma-miR156a-3p	GCUCACUUCUCUCUCUGUCAGU	0.00	5.26	0.00	33.25	3.40	NA	6.06	TRUE	NA	TRUE
mac-miR156a-5p	ath-miR156a-5p	UGACAGAAGAGAGUGAGCAC	6392.33	4716.52	4721.17	13301.73	-0.44	-0.44	1.06	FALSE	FALSE	TRUE
mac-miR156b.1	cca-miR156b	UGACAGAAGAGAGUGAGCAUA	806.80	679.05	540.32	1343.47	-0.25	-0.58	0.74	FALSE	FALSE	FALSE
mac-miR156b.2	pta-miR156b	CAGAAGAUAGAGAGCACAAC	6.21	0.00	0.00	0.00	-3.63	-3.63	-3.63	TRUE	TRUE	FALSE
mac-miR156b-3p.1	bdi-miR156b-3p	GCUCACUUCUCUCUCUGUCACC	0.00	5.26	0.00	33.25	3.40	NA	6.06	TRUE	NA	TRUE
mac-miR156c-3p.2	osa-miR156c-3p	GCUCACUUCUCUCUCUGUCAGC	0.00	5.26	0.00	33.25	3.40	NA	6.06	TRUE	NA	TRUE
mac-miR156e-3p	zma-miR156e-3p	GCUCACUGCUCUCUCUGUCAUC	68.27	57.90	88.58	133.02	-0.24	0.38	0.96	FALSE	FALSE	FALSE
mac-miR156f-5p	stu-miR156f-5p	CUGACAGAAGAGAGUGAGCA	806.80	679.05	540.32	1343.47	-0.25	-0.58	0.74	FALSE	FALSE	FALSE
mac-miR156g.1	ath-miR156g	CGACAGAAGAGAGUGAGCAC	18.62	5.26	17.72	19.95	-1.82	-0.07	0.10	TRUE	FALSE	FALSE
mac-miR156g.2	gma-miR156g	ACAGAAGAUAGAGAGCACAG	6.21	0.00	0.00	0.00	-3.63	-3.63	-3.63	TRUE	TRUE	FALSE
mac-miR156g-5p	mtr-miR156g-5p	UUGACAGAAGAUAGAGGGCAC	0.00	5.26	0.00	NA	3.40	NA	NA	TRUE	NA	NA
mac-miR156h	vvi-miR156h	UGACAGAAGAGAGAGAGCAU	0.00	0.00	8.86	NA	NA	4.15	NA	NA	TRUE	NA
mac-miR156h-3p	bdi-miR156h-3p	GCUCACUGCUCUUCCUGUCAUC	12.41	0.00	8.86	6.65	-4.63	-0.49	-0.90	TRUE	FALSE	FALSE
mac-miR156k	osa-miR156k	UGACAGAAGAGAGAGAGCACA	0.00	10.53	0.00	6.65	4.40	NA	3.73	TRUE	NA	TRUE
mac-miR156k-5p	zma-miR156k-5p	UGACAGAAGAGAGCGAGCAC	186.18	147.39	115.15	359.15	-0.34	-0.69	0.95	FALSE	FALSE	FALSE
mac-miR156l-5p	osa-miR156l-5p	CGACAGAAGAGAGUGAGCAUA	6.21	5.26	17.72	6.65	-0.24	1.51	0.10	FALSE	TRUE	FALSE
mac-miR157a-5p	ath-miR157a-5p	UUGACAGAAGAUAGAGAGCAC	304.10	221.09	239.16	399.05	-0.46	-0.35	0.39	FALSE	FALSE	FALSE
mac-miR157d-3p	aly-miR157d-3p	GCUCUCUAUGCUUCUGUCAUC	111.71	73.70	124.01	152.97	-0.60	0.15	0.45	FALSE	FALSE	FALSE
mac-miR159	aqc-miR159	UUUGGACUGAAGGGAGCUCUA	37.24	42.11	44.29	46.56	0.18	0.25	0.32	FALSE	FALSE	FALSE
mac-miR159a.2	ath-miR159a	UUUGGAUUGAAGGGAGCUCUA	50400.10	52218.62	39363.77	49269.61	0.05	-0.36	-0.03	FALSE	FALSE	FALSE
mac-miR159a.3	mdm-miR159a	CUUGGAUUGAAGGGAGCUCC	67820.74	77259.34	65866.11	54510.49	0.19	-0.04	-0.32	FALSE	FALSE	FALSE
mac-miR159a.4	pta-miR159a	UUGGAUUGAAGGGAGCUCCA	67820.74	77259.34	65866.11	54510.49	0.19	-0.04	-0.32	FALSE	FALSE	FALSE
mac-miR159a.1	osa-miR159a.1	UUUGGAUUGAAGGGAGCUCUG	15279.53	18650.26	14526.68	16434.29	0.29	-0.07	0.11	FALSE	FALSE	FALSE
mac-miR159a-3p	bdi-miR159a-3p	CUUGGAUUGAAGGGAGCUCU	4145.70	4890.23	4499.73	3438.50	0.24	0.12	-0.27	FALSE	FALSE	FALSE
mac-miR159b	lus-miR159b	UUUGGAUUGAAGGGAGCUCUC	15279.53	18650.26	14526.68	16434.29	0.29	-0.07	0.11	FALSE	FALSE	FALSE
mac-miR159b-3p	ath-miR159b-3p	UUUGGAUUGAAGGGAGCUCUU	15279.53	18650.26	14526.68	16434.29	0.29	-0.07	0.11	FALSE	FALSE	FALSE
mac-miR159c.1	ath-miR159c	UUUGGAUUGAAGGGAGCUCCU	7428.75	8090.73	7201.34	6165.35	0.12	-0.04	-0.27	FALSE	FALSE	FALSE
mac-miR159c.2	osa-miR159c	AUUGGAUUGAAGGGAGCUCCA	5213.16	5911.44	5722.10	4456.08	0.18	0.13	-0.23	FALSE	FALSE	FALSE
mac-miR159c.3	pta-miR159c	CUUGGAUUGAAGGGAGCUCCC	67820.74	77259.34	65866.11	54510.49	0.19	-0.04	-0.32	FALSE	FALSE	FALSE
mac-miR159c.4	sof-miR159c	CUUGGAUUGAAGGGAGCUCCU	68937.85	78638.50	67044.19	55488.17	0.19	-0.04	-0.31	FALSE	FALSE	FALSE
mac-miR159d	osa-miR159d	AUUGGAUUGAAGGGAGCUCCG	5213.16	5911.44	5722.10	4456.08	0.18	0.13	-0.23	FALSE	FALSE	FALSE
mac-miR159e	osa-miR159e	AUUGGAUUGAAGGGAGCUCCU	5368.31	6021.99	5846.10	4502.64	0.17	0.12	-0.25	FALSE	FALSE	FALSE
mac-miR159f	osa-miR159f	CUUGGAUUGAAGGGAGCUCUA	5709.65	6379.94	5544.94	4602.40	0.16	-0.04	-0.31	FALSE	FALSE	FALSE
mac-miR159h-3p	zma-miR159h-3p	UUUGGAGUGAAGGGAGCUCUG	0.00	5.26	0.00	NA	3.40	NA	NA	TRUE	NA	NA
mac-miR160.1	aau-miR160	UGGCAUACAGGGAGCCAGGCA	316.51	410.59	310.02	312.59	0.38	-0.03	-0.02	FALSE	FALSE	FALSE
mac-miR160.2	csi-miR160	GCCUGGCUCCCUGUAUGCCAU	316.51	410.59	310.02	312.59	0.38	-0.03	-0.02	FALSE	FALSE	FALSE
mac-miR160a-3p	osa-miR160a-3p	GCGUGCAAGGAGCCAAGCAUG	12.41	31.58	35.43	26.60	1.35	1.51	1.10	TRUE	TRUE	TRUE
mac-miR160a-5p	ath-miR160a-5p	UGCCUGGCUCCCUGUAUGCCA	310.31	400.06	301.16	299.29	0.37	-0.04	-0.05	FALSE	FALSE	FALSE
mac-miR160b.1	gma-miR160b	UGCCUGGCUCCCUGUAUGCC	322.72	415.85	310.02	312.59	0.37	-0.06	-0.05	FALSE	FALSE	FALSE
mac-miR160b.2	ppt-miR160b	CGCCUGGCUCCCUGUAUGCCA	0.00	5.26	8.86	NA	3.40	4.15	NA	TRUE	TRUE	NA
mac-miR160d-3p	osa-miR160d-3p	GCGUGCGAGGAGCCAAGCAUG	6.21	47.38	17.72	33.25	2.93	1.51	2.42	TRUE	TRUE	TRUE
mac-miR160e-5p	osa-miR160e-5p	UGCCUGGCUCCCUGUAUGCCG	179.98	163.18	186.01	192.88	-0.14	0.05	0.10	FALSE	FALSE	FALSE
mac-miR160f-5p	osa-miR160f-5p	UGCCUGGCUCCCUGAAUGCCA	266.86	315.84	194.87	259.38	0.24	-0.45	-0.04	FALSE	FALSE	FALSE
mac-miR160h	ptc-miR160h	UGCCUGGCUCCCUGCAUGCCA	0.00	5.26	0.00	6.65	3.40	NA	3.73	TRUE	NA	TRUE
mac-miR162.1	bdi-miR162	UCGAUAAACCUCUGCAUCCGG	11028.32	11549.16	11134.17	12330.70	0.07	0.01	0.16	FALSE	FALSE	FALSE
mac-miR162.2	cln-miR162	UUGAUAAACCUCUGCAUCCAG	6.21	0.00	0.00	6.65	-3.63	-3.63	0.10	TRUE	TRUE	FALSE
mac-miR162-3p	zma-miR162-3p	UCGAUAAACCUCUGCAUCCA	4443.60	3926.92	3711.39	3651.33	-0.18	-0.26	-0.28	FALSE	FALSE	FALSE
mac-miR162a-3p	ath-miR162a-3p	UCGAUAAACCUCUGCAUCCAG	4443.60	3926.92	3711.39	3644.67	-0.18	-0.26	-0.29	FALSE	FALSE	FALSE
mac-miR164	vun-miR164	UGGAGAAGGGGAGCACGUGCA	6.21	0.00	0.00	6.65	-3.63	-3.63	0.10	TRUE	TRUE	FALSE
mac-miR164a.1	ath-miR164a	UGGAGAAGCAGGGCACGUGCA	353.75	468.49	380.88	485.51	0.41	0.11	0.46	FALSE	FALSE	FALSE
mac-miR164a.2	hci-miR164a	UGGAGAAGCAGGGCACGUGAA	6.21	52.64	0.00	26.60	3.08	-3.63	2.10	TRUE	TRUE	TRUE
mac-miR164b	gma-miR164b	UGGAGAAGCAGGGCACGUGC	353.75	468.49	380.88	485.51	0.41	0.11	0.46	FALSE	FALSE	FALSE
mac-miR164c	osa-miR164c	UGGAGAAGCAGGGUACGUGCA	266.86	84.22	141.72	146.32	-1.66	-0.91	-0.87	TRUE	FALSE	FALSE
mac-miR164c-5p	ath-miR164c-5p	UGGAGAAGCAGGGCACGUGCG	111.71	131.60	35.43	133.02	0.24	-1.66	0.25	FALSE	TRUE	FALSE
mac-miR164d	osa-miR164d	UGGAGAAGCAGGGCACGUGCU	111.71	131.60	35.43	133.02	0.24	-1.66	0.25	FALSE	TRUE	FALSE
mac-miR164e	osa-miR164e	UGGAGAAGCAGGGCACGUGAG	24.82	57.90	8.86	59.86	1.22	-1.49	1.27	TRUE	TRUE	TRUE
mac-miR164h-5p	zma-miR164h-5p	UGGAGAAGCAGGGCACGUGUG	86.89	126.34	26.57	152.97	0.54	-1.71	0.82	FALSE	TRUE	FALSE
mac-miR166a	vvi-miR166a	UCGGACCAGGCUUCAUUCC	26853.99	22766.69	22117.76	25353.10	-0.24	-0.28	-0.08	FALSE	FALSE	FALSE
mac-miR166a-3p	ath-miR166a-3p	UCGGACCAGGCUUCAUUCCCC	43331.30	44370.03	44129.23	47340.86	0.03	0.03	0.13	FALSE	FALSE	FALSE
mac-miR166b	mtr-miR166b	UCGGACCAGGCUUCAUUCCUA	26853.99	22766.69	22117.76	25353.10	-0.24	-0.28	-0.08	FALSE	FALSE	FALSE
mac-miR166b-5p	osa-miR166b-5p	GGAAUGUUGUCUGGCUCGGGG	24.82	31.58	35.43	26.60	0.35	0.51	0.10	FALSE	FALSE	FALSE
mac-miR166c	gra-miR166c	UAUAUGAUCUCGGACCAGGCU	676.47	573.77	593.47	645.13	-0.24	-0.19	-0.07	FALSE	FALSE	FALSE
mac-miR166d-5p	osa-miR166d-5p	GGAAUGUUGUCUGGCUCGAGG	3432.00	4895.50	3578.52	4236.60	0.51	0.06	0.30	FALSE	FALSE	FALSE
mac-miR166e	cpa-miR166e	GGACCAGGCUUCAUUCCCC	42598.97	43269.86	43172.60	46469.60	0.02	0.02	0.13	FALSE	FALSE	FALSE
mac-miR166g-3p	osa-miR166g-3p	UCGGACCAGGCUUCAUUCCUC	27114.64	23045.68	22418.93	25605.83	-0.23	-0.27	-0.08	FALSE	FALSE	FALSE
mac-miR166h-3p	zma-miR166h-3p	UCGGACCAGGCUUCAUUCCC	42716.89	43364.61	43314.32	46576.01	0.02	0.02	0.12	FALSE	FALSE	FALSE
mac-miR166i.1	cme-miR166i	UCGGACCAGGCUUCAUUCUC	1743.93	1605.51	1399.52	1775.78	-0.12	-0.32	0.03	FALSE	FALSE	FALSE
mac-miR166i.2	mes-miR166i	UUGGACCAGGCUUCAUUCCCC	6.21	5.26	0.00	0.00	-0.24	-3.63	-3.63	FALSE	TRUE	FALSE
mac-miR166j	ppt-miR166j	UCCGGACCAGGCUUCAUUCCC	74.47	57.90	62.00	46.56	-0.36	-0.26	-0.68	FALSE	FALSE	FALSE
mac-miR166m.1	gma-miR166m	CGGACCAGGCUUCAUUCCCC	49.65	36.85	35.43	39.91	-0.43	-0.49	-0.32	FALSE	FALSE	FALSE
mac-miR166m.2	osa-miR166m	UCGGACCAGGCUUCAUUCCCU	3605.77	3347.89	2852.19	3598.12	-0.11	-0.34	0.00	FALSE	FALSE	FALSE
mac-miR166n	ptc-miR166n	UCGGACCAGGCUUCAUUCCUU	26853.99	22766.69	22117.76	25353.10	-0.24	-0.28	-0.08	FALSE	FALSE	FALSE
mac-miR166u	gma-miR166u	UCUCGGACCAGGCUUCAUUC	66740.87	63594.06	63385.95	69302.02	-0.07	-0.07	0.05	FALSE	FALSE	FALSE
mac-miR167.1	aqc-miR167	UCAAGCUGCCAGCAUGAUCUA	6.21	15.79	0.00	19.95	1.35	-3.63	1.68	TRUE	TRUE	TRUE
mac-miR167.2	ppt-miR167	GGAAGCUGCCAGCAUGAUCCU	0.00	0.00	0.00	6.65	NA	NA	3.73	NA	NA	TRUE
mac-miR167a-5p	ath-miR167a-5p	UGAAGCUGCCAGCAUGAUCUA	1805.99	2489.86	1780.40	2587.19	0.46	-0.02	0.52	FALSE	FALSE	FALSE
mac-miR167b-5p	sly-miR167b-5p	UAAAGCUGCCAGCAUGAUCUGG	533.73	431.65	451.74	731.60	-0.31	-0.24	0.45	FALSE	FALSE	FALSE
mac-miR167c.1	gra-miR167c	UCAGAUGAAGCUGCCAGCAUGA	844.04	1100.17	788.34	1197.16	0.38	-0.10	0.50	FALSE	FALSE	FALSE
mac-miR167c.2	vvi-miR167c	UGAAGCUGCCAGCAUGAUCUC	844.04	1100.17	788.34	1197.16	0.38	-0.10	0.50	FALSE	FALSE	FALSE
mac-miR167c-5p	ath-miR167c-5p	UAAGCUGCCAGCAUGAUCUUG	0.00	10.53	0.00	6.65	4.40	NA	3.73	TRUE	NA	TRUE
mac-miR167d.1	ath-miR167d	UGAAGCUGCCAGCAUGAUCUGG	3419.59	3732.16	3357.08	5067.96	0.13	-0.03	0.57	FALSE	FALSE	FALSE
mac-miR167d.2	bna-miR167d	UGAAGCUGCCAGCAUGAUCU	2048.03	2474.07	1984.13	3185.76	0.27	-0.05	0.64	FALSE	FALSE	FALSE
mac-miR167d-5p	osa-miR167d-5p	UGAAGCUGCCAGCAUGAUCUG	18314.33	16365.69	20231.07	31644.82	-0.16	0.14	0.79	FALSE	FALSE	FALSE
mac-miR167f-5p	ptc-miR167f-5p	UGAAGCUGCCAGCAUGAUCUU	844.04	1100.17	788.34	1197.16	0.38	-0.10	0.50	FALSE	FALSE	FALSE
mac-miR167h	gma-miR167h	AUCAUGCUGGCAGCUUCAACUG	844.04	1100.17	788.34	1197.16	0.38	-0.10	0.50	FALSE	FALSE	FALSE
mac-miR167i	gma-miR167i	UCAUGCUGGCAGCUUCAACUGG	403.40	352.69	239.16	445.61	-0.19	-0.75	0.14	FALSE	FALSE	FALSE
mac-miR168a-5p	ath-miR168a-5p	UCGCUUGGUGCAGGUCGGGAA	3742.30	2863.60	3215.36	3611.42	-0.39	-0.22	-0.05	FALSE	FALSE	FALSE
mac-miR168b.1	bna-miR168b	UCGCUUGGUGCAGGUCGAGAA	6.21	10.53	0.00	0.00	0.76	-3.63	-3.63	FALSE	TRUE	FALSE
mac-miR168b.2	gma-miR168b	UCGCUUGGUGCAGGUCGGG	3742.30	2858.34	3206.50	3604.77	-0.39	-0.22	-0.05	FALSE	FALSE	FALSE
mac-miR169a-3p	stu-miR169a-3p	GCAGUCUCCUUGGCUACU	0.00	0.00	8.86	26.60	NA	4.15	5.73	NA	TRUE	TRUE
mac-miR169a-5p	ath-miR169a-5p	CAGCCAAGGAUGACUUGCCGA	0.00	0.00	8.86	NA	NA	4.15	NA	NA	TRUE	NA
mac-miR169b-5p	ath-miR169b-5p	CAGCCAAGGAUGACUUGCCGG	6.21	0.00	17.72	6.65	-3.63	1.51	0.10	TRUE	TRUE	FALSE
mac-miR169c	gma-miR169c	AAGCCAAGGAUGACUUGCCGA	0.00	0.00	0.00	6.65	NA	NA	3.73	NA	NA	TRUE
mac-miR169c-3p	stu-miR169c-3p	GCAGUCUCCUUGGCUACC	0.00	0.00	0.00	6.65	NA	NA	3.73	NA	NA	TRUE
mac-miR169d-5p	sbi-miR169d-5p	UAGCCAAGGAUGACUUGCCU	0.00	0.00	0.00	6.65	NA	NA	3.73	NA	NA	TRUE
mac-miR169e.1	gma-miR169e	AGCCAAGGAUGACUUGCCGG	0.00	5.26	0.00	NA	3.40	NA	NA	TRUE	NA	NA
mac-miR169e.2	osa-miR169e	UAGCCAAGGAUGACUUGCCGG	18.62	26.32	8.86	19.95	0.50	-1.07	0.10	FALSE	FALSE	FALSE
mac-miR169f.1	osa-miR169f.1	UAGCCAAGGAUGACUUGCCUA	0.00	0.00	0.00	6.65	NA	NA	3.73	NA	NA	TRUE
mac-miR169h	ath-miR169h	UAGCCAAGGAUGACUUGCCUG	0.00	0.00	0.00	6.65	NA	NA	3.73	NA	NA	TRUE
mac-miR169h-3p	aly-miR169h-3p	GGCAGUCUCCUUGGCUAUU	0.00	0.00	0.00	6.65	NA	NA	3.73	NA	NA	TRUE
mac-miR169m	bdi-miR169m	UAGCCAAGGAUGACUUGCCG	18.62	26.32	8.86	19.95	0.50	-1.07	0.10	FALSE	FALSE	FALSE
mac-miR169p	osa-miR169p	UAGCCAAGGACAAACUUGCCGG	0.00	10.53	0.00	6.65	4.40	NA	3.73	TRUE	NA	TRUE
mac-miR169s	ptc-miR169s	UCAGCCAAGGAUGACUUGCCG	6.21	5.26	17.72	13.30	-0.24	1.51	1.10	FALSE	TRUE	FALSE
mac-miR169u	gma-miR169u	CAGCCAAGGAUGACUUGCCGU	0.00	0.00	8.86	NA	NA	4.15	NA	NA	TRUE	NA
mac-miR169v	gma-miR169v	CAGCCAAGGAUGACUUGCC	6.21	5.26	17.72	13.30	-0.24	1.51	1.10	FALSE	TRUE	FALSE
mac-miR171	ctr-miR171	UUGAGCCGCGUCAAUAUCUCC	37.24	15.79	62.00	53.21	-1.24	0.74	0.51	TRUE	FALSE	FALSE
mac-miR171a.1	gma-miR171a	UGAGCCGUGCCAAUAUCACGA	0.00	0.00	0.00	6.65	NA	NA	3.73	NA	NA	TRUE
mac-miR171a.2	htu-miR171a	UGAUUGAGCCGUGCCAAUAU	422.02	510.61	345.45	904.52	0.27	-0.29	1.10	FALSE	FALSE	TRUE
mac-miR171a.3	ppt-miR171a	UGAGCCGCGCCAAUAUCACAU	0.00	5.26	8.86	NA	3.40	4.15	NA	TRUE	TRUE	NA
mac-miR171a-3p.1	ath-miR171a-3p	UGAUUGAGCCGCGCCAAUAUC	18.62	31.58	26.57	13.30	0.76	0.51	-0.49	FALSE	FALSE	FALSE
mac-miR171a-3p.2	zma-miR171a-3p	UGAUUGAGCCGCGCCAAUAU	6.21	0.00	8.86	0.00	-3.63	0.51	-3.63	TRUE	FALSE	FALSE
mac-miR171b.1	mtr-miR171b	UGAUUGAGCCGCGUCAAUAUC	0.00	5.26	0.00	6.65	3.40	NA	3.73	TRUE	NA	TRUE
mac-miR171b.2	osa-miR171b	UGAUUGAGCCGUGCCAAUAUC	422.02	510.61	345.45	904.52	0.27	-0.29	1.10	FALSE	FALSE	TRUE
mac-miR171b.3	ppt-miR171b	UUGAGCCGCGCCAAUAUCACA	0.00	5.26	8.86	NA	3.40	4.15	NA	TRUE	TRUE	NA
mac-miR171b-3p.1	ath-miR171b-3p	UUGAGCCGUGCCAAUAUCACG	0.00	0.00	0.00	6.65	NA	NA	3.73	NA	NA	TRUE
mac-miR171b-3p.2	stu-miR171b-3p	UUGAGCCGCGUCAAUAUCUCU	235.83	121.07	203.73	232.78	-0.96	-0.21	-0.02	FALSE	FALSE	FALSE
mac-miR171b-3p.3	zma-miR171b-3p	UUGAGCCGUGCCAAUAUCAC	0.00	0.00	0.00	6.65	NA	NA	3.73	NA	NA	TRUE
mac-miR171c.1	mtr-miR171c	UGAUUGAGCCGUGCCAAUAUU	18.62	5.26	17.72	39.91	-1.82	-0.07	1.10	TRUE	FALSE	TRUE
mac-miR171c.2	ptc-miR171c	AGAUUGAGCCGCGCCAAUAUC	229.63	189.50	177.15	166.27	-0.28	-0.37	-0.47	FALSE	FALSE	FALSE
mac-miR171d	sly-miR171d	UUGAGCCGCGCCAAUAUCAC	229.63	189.50	177.15	166.27	-0.28	-0.37	-0.47	FALSE	FALSE	FALSE
mac-miR171f	aqc-miR171f	UAAUUGAGCCGUGCCAAUAUC	0.00	5.26	0.00	6.65	3.40	NA	3.73	TRUE	NA	TRUE
mac-miR171f.1	sbi-miR171f	AUGAGCCGAACCAAUAUCACU	0.00	5.26	0.00	NA	3.40	NA	NA	TRUE	NA	NA
mac-miR171f.2	vvi-miR171f	UUGAGCCGCGCCAAUAUCACU	229.63	189.50	177.15	166.27	-0.28	-0.37	-0.47	FALSE	FALSE	FALSE
mac-miR171h.1	osa-miR171h	GUGAGCCGAACCAAUAUCACU	0.00	5.26	0.00	NA	3.40	NA	NA	TRUE	NA	NA
mac-miR171h.2	vvi-miR171h	UGGUUGAGCCGCGCCAAUAUC	0.00	0.00	8.86	NA	NA	4.15	NA	NA	TRUE	NA
mac-miR171i	mdm-miR171i	UGAGCCGAACCAAUAUCACUC	0.00	5.26	0.00	NA	3.40	NA	NA	TRUE	NA	NA
mac-miR171i-3p	osa-miR171i-3p	GGAUUGAGCCGCGUCAAUAUC	242.04	121.07	203.73	232.78	-1.00	-0.25	-0.06	FALSE	FALSE	FALSE
mac-miR171i-5p	osa-miR171i-5p	AGGUAUUGGCGUGCCUCAAUC	6.21	5.26	0.00	0.00	-0.24	-3.63	-3.63	FALSE	TRUE	FALSE
mac-miR171k	ptc-miR171k	GGAUUGAGCCGCGCCAAUAUC	0.00	0.00	8.86	NA	NA	4.15	NA	NA	TRUE	NA
mac-miR171m	gma-miR171m	UUGAGCCGCGUCAAUAUCUCA	6.21	0.00	0.00	6.65	-3.63	-3.63	0.10	TRUE	TRUE	FALSE
mac-miR171n	gma-miR171n	UUGAGCCGCGUCAAUAUCUUA	0.00	5.26	0.00	6.65	3.40	NA	3.73	TRUE	NA	TRUE
mac-miR172a.1	ath-miR172a	AGAAUCUUGAUGAUGCUGCAU	316.51	257.93	150.58	305.94	-0.30	-1.07	-0.05	FALSE	TRUE	FALSE
mac-miR172a.2	mtr-miR172a	AGAAUCCUGAUGAUGCUGCAG	0.00	0.00	0.00	6.65	NA	NA	3.73	NA	NA	TRUE
mac-miR172a-5p	aly-miR172a-5p	GUGGCAUCAUCAAGAUUCACA	0.00	0.00	0.00	13.30	NA	NA	4.73	NA	NA	TRUE
mac-miR172b	sbi-miR172b	GGAAUCUUGAUGAUGCUGCA	0.00	15.79	0.00	19.95	4.98	NA	5.32	TRUE	NA	TRUE
mac-miR172c.1	ath-miR172c	AGAAUCUUGAUGAUGCUGCAG	24.82	10.53	17.72	33.25	-1.24	-0.49	0.42	TRUE	FALSE	FALSE
mac-miR172c.2	osa-miR172c	UGAAUCUUGAUGAUGCUGCAC	0.00	0.00	8.86	13.30	NA	4.15	4.73	NA	TRUE	TRUE
mac-miR172c-3p	stu-miR172c-3p	AGAAUCUUGAUGAUGCUGC	18.62	10.53	0.00	13.30	-0.82	-5.22	-0.49	FALSE	TRUE	FALSE
mac-miR172d	gma-miR172d	GGAAUCUUGAUGAUGCUGCAGC	0.00	0.00	0.00	13.30	NA	NA	4.73	NA	NA	TRUE
mac-miR172e-3p.1	aly-miR172e-3p	GAAUCUUGAUGAUGCUGCAU	0.00	15.79	0.00	19.95	4.98	NA	5.32	TRUE	NA	TRUE
mac-miR172e-3p.2	ath-miR172e-3p	GGAAUCUUGAUGAUGCUGCAU	0.00	15.79	0.00	19.95	4.98	NA	5.32	TRUE	NA	TRUE
mac-miR172f	cme-miR172f	UGAAUCUUGAUGAUGCCGCAC	12.41	10.53	17.72	0.00	-0.24	0.51	-4.63	FALSE	FALSE	TRUE
mac-miR172j	lus-miR172j	GCAGCAUCAUCAAGAUUCCCA	0.00	0.00	0.00	13.30	NA	NA	4.73	NA	NA	TRUE
mac-miR319	pta-miR319	UUGGACUGAAGGGAGCUCC	484.08	463.23	451.74	665.09	-0.06	-0.10	0.46	FALSE	FALSE	FALSE
mac-miR319a.1	ath-miR319a	UUGGACUGAAGGGAGCUCCCU	35902.54	33157.77	36006.69	25133.62	-0.11	0.00	-0.51	FALSE	FALSE	FALSE
mac-miR319a.2	ppt-miR319a	CUUGGACUGAAGGGAGCUCC	775.77	715.90	699.76	864.61	-0.12	-0.15	0.16	FALSE	FALSE	FALSE
mac-miR319a-3p	mtr-miR319a-3p	UUGGACUGAAGGGAGCUCCC	35207.46	32989.32	36015.55	24368.77	-0.09	0.03	-0.53	FALSE	FALSE	FALSE
mac-miR319a-5p	mtr-miR319a-5p	AGAGCUUCCUUCAGUCCACUC	43.44	52.64	70.86	53.21	0.28	0.71	0.29	FALSE	FALSE	FALSE
mac-miR319c	ath-miR319c	UUGGACUGAAGGGAGCUCCUU	8229.35	8648.71	9318.34	7103.12	0.07	0.18	-0.21	FALSE	FALSE	FALSE
mac-miR319e	ptc-miR319e	UUGGACUGAAGGGAGCUCCU	8223.14	8648.71	9291.76	7076.52	0.07	0.18	-0.22	FALSE	FALSE	FALSE
mac-miR319q	gma-miR319q	UGGACUGAAGGGAGCUCCUUC	9321.63	9606.75	10469.84	8000.99	0.04	0.17	-0.22	FALSE	FALSE	FALSE
mac-miR3630-3p.1	han-miR3630-3p	UGUGGGAAUCUCUCUGAUGCUU	0.00	0.00	8.86	NA	NA	4.15	NA	NA	TRUE	NA
mac-miR3630-3p.2	vvi-miR3630-3p	UUUGGGAAUCUCUCUGAUGCAC	24.82	15.79	8.86	0.00	-0.65	-1.49	-5.63	FALSE	TRUE	TRUE
mac-miR3711	pab-miR3711	UGGCGCUAGAAGGAGGGCCU	31.03	15.79	70.86	33.25	-0.97	1.19	0.10	FALSE	TRUE	FALSE
mac-miR390.1	atr-miR390.1	UAAAGCUCAGGAGGGAUAGCG	86.89	100.02	124.01	139.67	0.20	0.51	0.68	FALSE	FALSE	FALSE
mac-miR390a-5p	ath-miR390a-5p	AAGCUCAGGAGGGAUAGCGCC	86.89	100.02	124.01	139.67	0.20	0.51	0.68	FALSE	FALSE	FALSE
mac-miR390e	gma-miR390e	AGCUCAGGAGGGAUAGCGCC	86.89	100.02	124.01	139.67	0.20	0.51	0.68	FALSE	FALSE	FALSE
mac-miR393-5p	ata-miR393-5p	UUCCAAAGGGAUCGCAUUGAU	37.24	10.53	8.86	53.21	-1.82	-2.07	0.51	TRUE	TRUE	FALSE
mac-miR393a.1	osa-miR393a	UCCAAAGGGAUCGCAUUGAUC	0.00	0.00	8.86	6.65	#NAME?	4.15	3.73	NA	TRUE	TRUE
mac-miR393a.2	ppe-miR393a	CAUCCAAAGGGAUCGCAUUGA	0.00	0.00	8.86	6.65	NA	4.15	3.73	NA	TRUE	TRUE
mac-miR393a-5p	ath-miR393a-5p	UCCAAAGGGAUCGCAUUGAUCC	0.00	0.00	8.86	6.65	NA	4.15	3.73	NA	TRUE	TRUE
mac-miR394a	ath-miR394a	UUGGCAUUCUGUCCACCUCC	242.04	163.18	124.01	266.03	-0.57	-0.96	0.14	FALSE	FALSE	FALSE
mac-miR395a.1	ath-miR395a	CUGAAGUGUUUGGGGGAACUC	198.60	110.54	292.31	252.73	-0.85	0.56	0.35	FALSE	FALSE	FALSE
mac-miR395a.2	mtr-miR395a	AUGAAGUGUUUGGGGGAACUC	186.18	89.49	265.73	239.43	-1.06	0.51	0.36	FALSE	FALSE	FALSE
mac-miR395b.1	osa-miR395b	GUGAAGUGUUUGGGGGAACUC	186.18	89.49	265.73	239.43	-1.06	0.51	0.36	FALSE	FALSE	FALSE
mac-miR395b.2	tae-miR395b	UGAAGUGUUUGGGGGAACUC	198.60	105.28	292.31	252.73	-0.92	0.56	0.35	FALSE	FALSE	FALSE
mac-miR395b-3p	ata-miR395b-3p	AAGUGUUUGGGGGAACUC	186.18	89.49	265.73	239.43	-1.06	0.51	0.36	FALSE	FALSE	FALSE
mac-miR395d	gma-miR395d	UGAAGUGUUUGGGGGAACUUU	86.89	42.11	168.30	139.67	-1.05	0.95	0.68	FALSE	FALSE	FALSE
mac-miR395g	mtr-miR395g	UUGAAGUGUUUGGGGGAACUC	186.18	89.49	265.73	239.43	-1.06	0.51	0.36	FALSE	FALSE	FALSE
mac-miR395h	mtr-miR395h	AUGAAGUGUUUGGGGGAACUU	86.89	42.11	168.30	139.67	-1.05	0.95	0.68	FALSE	FALSE	FALSE
mac-miR395t	osa-miR395t	GUGAAGUGUUUGGGGAAACUC	0.00	0.00	8.86	NA	NA	4.15	NA	NA	TRUE	NA
mac-miR396	smo-miR396	UUCCACGGCUUUCUUGAACC	6.21	10.53	0.00	33.25	0.76	-3.63	2.42	FALSE	TRUE	TRUE
mac-miR396a.1	atr-miR396a	UUCCACAGGCUUUCUUGAACAU	74.47	63.17	53.15	39.91	-0.24	-0.49	-0.90	FALSE	FALSE	FALSE
mac-miR396a.2	mdm-miR396a	UUCCACAGCUUUCUUGAACAG	2364.54	1926.61	2373.87	1955.35	-0.30	0.01	-0.27	FALSE	FALSE	FALSE
mac-miR396a.3	ppe-miR396a	UUCCACAGCUUUCUUGAACGU	2668.64	2300.36	2559.89	2261.29	-0.21	-0.06	-0.24	FALSE	FALSE	FALSE
mac-miR396a.4	vvi-miR396a	UUCCACAGCUUUCUUGAACUA	6119.26	5948.29	6714.16	6138.75	-0.04	0.13	0.00	FALSE	FALSE	FALSE
mac-miR396a-3p.1	ath-miR396a-3p	GUUCAAUAAAGCUGUGGGAAG	1222.61	1884.50	1532.39	1483.14	0.62	0.33	0.28	FALSE	FALSE	FALSE
mac-miR396a-3p.2	cca-miR396a-3p	GUUCAAGAAAGCUGUGGGAAA	49.65	31.58	115.15	46.56	-0.65	1.21	-0.09	FALSE	TRUE	FALSE
mac-miR396a-3p.3	gma-miR396a-3p	UUCAAUAAAGCUGUGGGAAG	1222.61	1879.24	1532.39	1483.14	0.62	0.33	0.28	FALSE	FALSE	FALSE
mac-miR396a-3p.4	osa-miR396a-3p	GUUCAAUAAAGCUGUGGGAA	422.02	642.20	628.90	591.93	0.61	0.58	0.49	FALSE	FALSE	FALSE
mac-miR396a-5p	ath-miR396a-5p	UUCCACAGCUUUCUUGAACUG	22329.71	21355.94	20461.37	19274.21	-0.06	-0.13	-0.21	FALSE	FALSE	FALSE
mac-miR396b	vvi-miR396b	UUCCACAGCUUUCUUGAACU	7801.12	7327.45	8246.55	7355.86	-0.09	0.08	-0.08	FALSE	FALSE	FALSE
mac-miR396b-5p	ath-miR396b-5p	UUCCACAGCUUUCUUGAACUU	7677.00	7222.17	8211.12	7196.24	-0.09	0.10	-0.09	FALSE	FALSE	FALSE
mac-miR396c	cca-miR396c	UUCAAGAAAGCUGUGGGAAAA	49.65	31.58	115.15	46.56	-0.65	1.21	-0.09	FALSE	TRUE	FALSE
mac-miR396c-3p	osa-miR396c-3p	GGUCAAGAAAGCUGUGGGAAG	6.21	10.53	0.00	0.00	0.76	-3.63	-3.63	FALSE	TRUE	FALSE
mac-miR396e-5p	osa-miR396e-5p	UCCACAGGCUUUCUUGAACUG	5796.54	4269.08	5677.81	5852.76	-0.44	-0.03	0.01	FALSE	FALSE	FALSE
mac-miR396f	ptc-miR396f	UUCCACGGCUUUCUUGAACUG	1930.11	1689.74	1603.25	1835.64	-0.19	-0.27	-0.07	FALSE	FALSE	FALSE
mac-miR396f-5p	osa-miR396f-5p	UCUCCACAGGCUUUCUUGAACU	6026.17	4411.21	5828.39	6078.89	-0.45	-0.05	0.01	FALSE	FALSE	FALSE
mac-miR396g	osa-miR396g	UCCACAGGCUUUCUUGAACGG	235.83	142.13	221.44	133.02	-0.73	-0.09	-0.83	FALSE	FALSE	FALSE
mac-miR396g-3p	zma-miR396g-3p	GUUCAAGAAAGCUGUGGAAGA	22236.61	21219.08	20346.22	19194.40	-0.07	-0.13	-0.21	FALSE	FALSE	FALSE
mac-miR396g-5p.1	ptc-miR396g-5p	UUCCACGGCUUUCUUGAACUU	74.47	42.11	53.15	66.51	-0.82	-0.49	-0.16	FALSE	FALSE	FALSE
mac-miR396g-5p.2	zma-miR396g-5p	UCCCACAGCUUUAUUGAACUG	260.66	394.80	442.89	432.31	0.60	0.76	0.73	FALSE	FALSE	FALSE
mac-miR396h	gma-miR396h	UCCACAGCUUUCUUGAACUG	22193.17	21255.93	20266.50	19181.10	-0.06	-0.13	-0.21	FALSE	FALSE	FALSE
mac-miR397.1	mes-miR397	UUUGAGUGCAGCGUUGAUGA	0.00	26.32	35.43	73.16	5.72	6.15	7.19	TRUE	TRUE	TRUE
mac-miR397.2	nta-miR397	AUUGAGUGCAGCGUUGAUGU	0.00	26.32	44.29	79.81	5.72	6.47	7.32	TRUE	TRUE	TRUE
mac-miR397.3	pab-miR397	UCAUUGAGUGCAGCGUUGACG	0.00	5.26	0.00	NA	3.40	NA	NA	TRUE	NA	NA
mac-miR397.4	sly-miR397	AUUGAGUGCAGCGUUGAUGA	0.00	26.32	35.43	73.16	5.72	6.15	7.19	TRUE	TRUE	TRUE
mac-miR397a.1	ath-miR397a	UCAUUGAGUGCAGCGUUGAUG	0.00	36.85	44.29	79.81	6.20	6.47	7.32	TRUE	TRUE	TRUE
mac-miR397a.2	atr-miR397a	UCCUUGAGUGCAGCGUUGAUG	0.00	26.32	35.43	73.16	5.72	6.15	7.19	TRUE	TRUE	TRUE
mac-miR397a.3	hvu-miR397a	CCGUUGAGUGCAGCGUUGAUG	0.00	26.32	35.43	73.16	5.72	6.15	7.19	TRUE	TRUE	TRUE
mac-miR397b.1	osa-miR397b	UUAUUGAGUGCAGCGUUGAUG	0.00	26.32	35.43	73.16	5.72	6.15	7.19	TRUE	TRUE	TRUE
mac-miR397b.2	ptc-miR397b	CCAUUGAGUGCAGCGUUGAUG	0.00	26.32	35.43	73.16	5.72	6.15	7.19	TRUE	TRUE	TRUE
mac-miR397b-3p	hvu-miR397b-3p	AUCAACGCUGCACUCAACGGC	0.00	26.32	35.43	73.16	5.72	6.15	7.19	TRUE	TRUE	TRUE
mac-miR398	ahy-miR398	UGUGUUCUCAGGUCACCCCU	12.41	26.32	53.15	13.30	1.08	2.10	0.10	TRUE	TRUE	FALSE
mac-miR398a-3p.1	ath-miR398a-3p	UGUGUUCUCAGGUCACCCCUU	12.41	26.32	53.15	13.30	1.08	2.10	0.10	TRUE	TRUE	FALSE
mac-miR398a-3p.2	zma-miR398a-3p	UGUGUUCUCAGGUCGCCCCCG	6.21	0.00	0.00	0.00	-3.63	-3.63	-3.63	TRUE	TRUE	FALSE
mac-miR398b	osa-miR398b	UGUGUUCUCAGGUCGCCCCUG	304.10	384.27	425.17	545.37	0.34	0.48	0.84	FALSE	FALSE	FALSE
mac-miR398b-3p	ath-miR398b-3p	UGUGUUCUCAGGUCACCCCUG	6.21	0.00	8.86	0.00	-3.63	0.51	-3.63	TRUE	FALSE	FALSE
mac-miR399b	ath-miR399b	UGCCAAAGGAGAGUUGCCCUG	0.00	5.26	0.00	NA	3.40	NA	NA	TRUE	NA	NA
mac-miR399d	cme-miR399d	UGCCAAAGGAGAGUUGCCCUU	0.00	5.26	0.00	NA	3.40	NA	NA	TRUE	NA	NA
mac-miR399e	osa-miR399e	UGCCAAAGGAGAUUUGCCCAG	0.00	0.00	0.00	6.65	NA	NA	3.73	NA	NA	TRUE
mac-miR399j	osa-miR399j	UGCCAAAGGAGAGUUGCCCUA	12.41	15.79	8.86	13.30	0.35	-0.49	0.10	FALSE	FALSE	FALSE
mac-miR408-3p.1	ata-miR408-3p	UGCACUGCCUCUUCCCUGCC	80.68	73.70	62.00	53.21	-0.13	-0.38	-0.60	FALSE	FALSE	FALSE
mac-miR408-3p.2	ath-miR408-3p	AUGCACUGCCUCUUCCCUGGC	719.91	1489.70	1116.07	1636.11	1.05	0.63	1.18	TRUE	FALSE	TRUE
mac-miR408-3p.3	osa-miR408-3p	CUGCACUGCCUCUUCCCUGGC	719.91	1489.70	1116.07	1636.11	1.05	0.63	1.18	TRUE	FALSE	TRUE
mac-miR408-5p	bra-miR408-5p	GGGAGCCAGGGAAGAGGCAGU	719.91	1489.70	1116.07	1636.11	1.05	0.63	1.18	TRUE	FALSE	TRUE
mac-miR408b	ppt-miR408b	UGCACUGCCUCUUCCCUGGCU	670.26	1394.95	1062.93	1562.95	1.06	0.67	1.22	TRUE	FALSE	TRUE
mac-miR408d	gma-miR408d	UGCACUGCCUCUUCCCUGGC	744.74	1489.70	1116.07	1649.41	1.00	0.58	1.15	TRUE	FALSE	TRUE
mac-miR444a-3p.2	osa-miR444a-3p.2	UGCAGUUGCUGCCUCAAGCUU	6.21	15.79	8.86	0.00	1.35	0.51	-3.63	TRUE	FALSE	FALSE
mac-miR444f	osa-miR444f	UGCAGUUGUUGCCUCAAGCUU	62.06	36.85	35.43	53.21	-0.75	-0.81	-0.22	FALSE	FALSE	FALSE
mac-miR4995	gma-miR4995	AGGCAGUGGCUUGGUUAAGGG	37.24	10.53	26.57	33.25	-1.82	-0.49	-0.16	TRUE	FALSE	FALSE
mac-miR5083	osa-miR5083	AGACUACAAUUAUCUGAUCA	130.33	94.75	70.86	106.41	-0.46	-0.88	-0.29	FALSE	FALSE	FALSE
mac-miR5139	rgl-miR5139	AAACCUGGCUCUGAUACCA	43.44	36.85	88.58	33.25	-0.24	1.03	-0.39	FALSE	TRUE	FALSE
mac-miR5141	rgl-miR5141	AGACCCGACGCGACUGACAGAU	37.24	21.06	17.72	6.65	-0.82	-1.07	-2.49	FALSE	TRUE	TRUE
mac-miR5179	bdi-miR5179	UUUUGCUCAAGACCGCGCAAC	0.00	5.26	0.00	NA	3.40	NA	NA	TRUE	NA	NA
mac-miR5234	mtr-miR5234	UUUUGUUGUGGAUGGCAGAAG	0.00	5.26	0.00	NA	3.40	NA	NA	TRUE	NA	NA
mac-miR528-5p	osa-miR528-5p	UGGAAGGGGCAUGCAGAGGAG	1458.44	2084.53	1434.95	3451.80	0.52	-0.02	1.24	FALSE	FALSE	TRUE
mac-miR529-5p	zma-miR529-5p	AGAAGAGAGAGAGAGUACAGCCU	0.00	0.00	17.72	6.65	NA	5.15	3.73	NA	TRUE	TRUE
mac-miR529a	osa-miR529a	CUGUACCCUCUCUCUUCUUC	55.86	15.79	88.58	59.86	-1.82	0.67	0.10	TRUE	FALSE	FALSE
mac-miR529b	osa-miR529b	AGAAGAGAGAGAGAGUACAGCUU	130.33	63.17	115.15	152.97	-1.05	-0.18	0.23	FALSE	FALSE	FALSE
mac-miR529e	ppt-miR529e	AGAAGAGAGAGAGAGUACAGCCC	0.00	0.00	8.86	NA	NA	4.15	NA	NA	TRUE	NA
mac-miR530	stu-miR530	UCUGCAUUUGCACCUGCACCU	6.21	5.26	0.00	0.00	-0.24	-3.63	-3.63	FALSE	TRUE	FALSE
mac-miR530-5p	osa-miR530-5p	UGCAUUUGCACCUGCACCUA	12.41	10.53	0.00	6.65	-0.24	-4.63	-0.90	FALSE	TRUE	FALSE
mac-miR530a.1	ptc-miR530a	UGCAUUUGCACCUGCACCUU	6.21	5.26	0.00	0.00	-0.24	-3.63	-3.63	FALSE	TRUE	FALSE
mac-miR530a.2	tcc-miR530a	UGCAUUUGCACCUGCACCUC	6.21	5.26	0.00	0.00	-0.24	-3.63	-3.63	FALSE	TRUE	FALSE
mac-miR535a.1	mes-miR535a	UGACAACGAGAGAGAGCACGU	173.77	142.13	345.45	166.27	-0.29	0.99	-0.06	FALSE	FALSE	FALSE
mac-miR535a.2	ppt-miR535a	UGACAACGAGAGAGAGCACGC	1905.29	2105.59	2356.16	3325.43	0.14	0.31	0.80	FALSE	FALSE	FALSE
mac-miR535b	mes-miR535b	UGACAACGAGAGAGAGCACGG	173.77	142.13	345.45	166.27	-0.29	0.99	-0.06	FALSE	FALSE	FALSE
mac-miR535d	mdm-miR535d	UGACGACGAGAGAGAGCACGC	273.07	231.61	434.03	372.45	-0.24	0.67	0.45	FALSE	FALSE	FALSE
mac-miR5368	gma-miR5368	GGACAGUCUCAGGUAGACA	74.47	42.11	17.72	73.16	-0.82	-2.07	-0.03	FALSE	TRUE	FALSE
mac-miR5538	osa-miR5538	ACUGAACUCAAUCACUUGCUGC	0.00	0.00	0.00	6.65	NA	NA	3.73	NA	NA	TRUE
mac-miR5658	ath-miR5658	AUGAUGAUGAUGAUGAUGAAA	0.00	10.53	0.00	NA	4.40	NA	NA	TRUE	NA	NA
mac-miR6300	gma-miR6300	GUCGUUGUAGUAUAGUGG	757.15	589.57	584.61	571.97	-0.36	-0.37	-0.40	FALSE	FALSE	FALSE
mac-miR6478	ptc-miR6478	CCGACCUUAGCUCAGUUGGUG	484.08	373.74	761.77	518.77	-0.37	0.65	0.10	FALSE	FALSE	FALSE
mac-miR8155	cpa-miR8155	UAACCUGGCUCUGAUACCA	43.44	21.06	79.72	26.60	-1.05	0.88	-0.71	FALSE	FALSE	FALSE
mac-miR827	osa-miR827	UUAGAUGACCAUCAGCAAACA	37.24	5.26	62.00	79.81	-2.82	0.74	1.10	TRUE	FALSE	TRUE
mac-miR845	bdi-miR845	UGCUCUGAUACCAAUUGUUGG	12.41	0.00	0.00	0.00	-4.63	-4.63	-4.63	TRUE	TRUE	TRUE
mac-miR8577	atr-miR8577	AAAAGGCUCAGAUGAUGAUGAU	0.00	10.53	0.00	NA	4.40	NA	NA	TRUE	NA	NA
mac-miR858a	ath-miR858a	UUUCGUUGUCUGUUCGACCUU	6.21	10.53	0.00	13.30	0.76	-3.63	1.10	FALSE	TRUE	FALSE
mac-miR858b	ath-miR858b	UUCGUUGUCUGUUCGACCUUG	6.21	10.53	0.00	13.30	0.76	-3.63	1.10	FALSE	TRUE	FALSE

Supplemental data S3 Information of the identified novel miRNAs

miRNA name	precursor_seq	mfe	position	mature_seq	RCK (Reads)	R5H (Reads)	R10 (Reads)	R25 (Reads)	RCK (TPM)	R5H (TPM)	R10 (TPM)	R25 (TPM)	log2.Fold_ change (R5H VS CK)	log2.Fold_ change (R10 VS RCK)	log2.Fold_ change (R25 VS RCK)	Signature	Signature	Signature
mac-nmiR1	uggacgauggacgaacaugaga	-60	1:5318068..5318386:+	ucauauucguacaucguccacc	425	416	229	376	2637.611	2189.813	2028.421	2500.725	-0.26853	-0.37887	-0.07699	FALSE	FALSE	FALSE
mac-nmiR10	uggggauaagaucgugcuucgu	-74.3	9:897951..898270:+	uaaguggcucuuaucccucaug	1	5	3	0	6.206144	26.31987	26.5732	0	2.084282	2.0982	-3.6338	TRUE	TRUE	FALSE
mac-nmiR11	uaggagagaugacaccggcuuc	-35.6	7:1377027..1377345:-	uaggagagaugacaccggcuu	1	8	1	0	6.206144	42.11179	8.857734	0	2.762354	0.51324	-3.6338	TRUE	FALSE	FALSE
mac-nmiR12	ugucggugucugcaaauggugg	-55.81	10:18765269..18765587:+	ugucggugucugcaaauggug	0	2	0	2	0	10.52795	0	13.30173	4.396152	NA	4.7335	TRUE	NA	TRUE
mac-nmiR13	uggagaagcaggguacgugcau	-35.6	5:10325589..10325907:+	caugugcccaucuucuccauc	0	3	2	1	0	15.79192	17.71547	6.650865	4.981115	5.1469	3.7335	TRUE	TRUE	TRUE
mac-nmiR16	gggcauuccgagaauugaacuc	-201	chrUn_random:119727866..119728183:-	caauucucggaaugccccu	0	1	0	1	0	5.263974	0	6.650865	3.396152	NA	3.7335	TRUE	NA	TRUE
mac-nmiR17	ugaagaggcagagaguggaaug	-64.1	1:11761961..11762279:+	uuuggcucucuucuucucaug	0	2	0	1	0	10.52795	0	6.650865	4.396152	NA	3.7335	TRUE	NA	TRUE
mac-nmiR2	ucaagcugccagcaugaucuga	-35.1	7:1098000..1098319:-	ucaagcugccagcaugaucuga	47	96	31	94	291.6888	505.3415	274.5898	625.1813	0.792728	-0.08715	1.0997	FALSE	FALSE	TRUE
mac-nmiR20	uuuuaaagccucucacgcggau	-98.8	8:33250233..33250551:+	uuuuaaagccucucacgcgga	3	4	3	3	18.61843	21.05589	26.5732	19.9526	0.177392	0.51324	0.099744	FALSE	FALSE	FALSE
mac-nmiR21	uggagaagcagggcacgugcac	-38.4	6:31952541..31952857:-	uggagaagcagggcacgugcac	0	0	0	1	0	0	0	6.650865	NA	NA	3.7335	NA	NA	TRUE
mac-nmiR22	ugacaggagagagugagcacac	-38.5	4:21077440..21077755:+	ugacaggagagagugagcac	11	5	8	14	68.26759	26.31987	70.86187	93.11211	-1.37515	0.053809	0.44767	TRUE	FALSE	FALSE
mac-nmiR23	uugugucaggcaauaguaccgc	-57.6	11:6469963..6470280:+	uugugucaggcaauaguacc	5	8	4	5	31.03072	42.11179	35.43094	33.25433	0.440426	0.19131	0.099744	FALSE	FALSE	FALSE
mac-nmiR24	cgauauuggugagguucaaucc	-45.7	1:13597555..13597873:-	cgauauuggugagguucaauc	6	7	1	2	37.23686	36.84782	8.857734	13.30173	-0.01525	-2.0717	-1.4852	FALSE	TRUE	TRUE
mac-nmiR25	uaaagcugccagcaugaucuga	-28.8	1:3895761..3896080:+	uaaagcugccagcaugaucuga	1043	901	742	1521	6473.008	4742.84	6572.439	10115.97	-0.44879	0.021992	0.64402	FALSE	FALSE	FALSE
mac-nmiR26	uccuauauuggcugggaccuaa	-13.4	5:2415607..2415922:-	uccuauauuggcugggaccu	11	11	3	11	68.26759	57.90371	26.5732	73.15952	-0.23765	-1.3612	0.099744	FALSE	TRUE	FALSE
mac-nmiR27	uuccacagcuuucuugaacggc	-32.6	chrUn_random:47515292..47515610:-	uuccacagcuuucuugaacgg	206	217	141	137	1278.466	1142.282	1248.941	911.1685	-0.1626	-0.03371	-0.48872	FALSE	FALSE	FALSE
mac-nmiR3	uggagaagcagggcacguguuc	-43.5	9:9325850..9326168:+	uggagaagcagggcacguguu	36	18	29	91	223.4212	94.75152	256.8743	605.2287	-1.23765	0.2013	1.4376	TRUE	FALSE	TRUE
mac-nmiR30	gaaauccggugcauaccgacua	-46.1	1:15852230..15852548:+	ucaguaugccucggauuucga	5	3	0	4	31.03072	15.79192	0	26.60346	-0.97461	-5.9556	-0.22218	FALSE	TRUE	FALSE
mac-nmiR31	ugacaacgagagagagcacgcc	-46.9	9:4509591..4509909:+	gugcuccuucucguugucacc	4	10	2	2	24.82458	52.63974	17.71547	13.30173	1.084282	-0.48676	-0.90026	TRUE	FALSE	FALSE
mac-nmiR32	gagagcuuccuucaguccacuc	-94.2	7:24546574..24546893:-	uggacugaagggagcuccucuu	242	245	162	194	1501.887	1289.674	1434.953	1290.268	-0.21987	-0.06577	-0.21921	FALSE	FALSE	FALSE
mac-nmiR33	ugccuggcucccuguacgccac	-49.2	5:303915..304232:+	ugccuggcucccuguacgcca	3	8	5	2	18.61843	42.11179	44.28867	13.30173	1.177392	1.2502	-0.48522	TRUE	TRUE	FALSE
mac-nmiR35	ucuuuaaucacgagaaaaacgu	-66	10:18723218..18723536:+	uuuuucucgugacuaaagacg	4	6	3	3	24.82458	31.58384	26.5732	19.9526	0.347317	0.098203	-0.31529	FALSE	FALSE	FALSE
mac-nmiR36	ggaaugaaguuugauccaagau	-40.02	2:17984457..17984776:-	ucggaccaggcuucauuccac	50	54	35	59	310.3072	284.2546	310.0207	392.401	-0.12661	-0.00133	0.33853	FALSE	FALSE	FALSE
mac-nmiR4	cgauauuggugagguucaaucc	-40.4	4:20873303..20873621:+	uugagccgcgccaauaucucu	28	35	26	23	173.772	184.2391	230.3011	152.9699	0.084282	0.40633	-0.18405	FALSE	FALSE	FALSE
mac-nmiR5	cugacgacgagagagagcacgc	-56	3:20865453..20865772:-	cugacgacgagagagagcacgc	24	21	18	36	148.9475	110.5434	159.4392	239.4311	-0.43029	0.098203	0.68471	FALSE	FALSE	FALSE
mac-nmiR6	cgugguauuggaucgguucaug	-33	chrUn_random:20350597..20350916:-	cgugguauuggaucgguucaug	36	26	21	28	223.4212	136.8633	186.0124	186.2242	-0.70713	-0.26437	-0.26283	FALSE	FALSE	FALSE
mac-nmiR8	uggagaagcaggucacgugcag	-41.3	11:23428257..23428575:+	uggagaagcaggucacgugca	6	1	6	7	37.23686	5.263974	53.14641	46.55606	-2.82261	0.51324	0.32214	TRUE	FALSE	FALSE
mac-nmiR9	acgccucuucaucgacccuccac	-35.2	3:27575460..27575776:-	agggacgaugaggagguauu	3	5	0	4	18.61843	26.31987	0	26.60346	0.49932	-5.2187	0.51478	FALSE	TRUE	FALSE

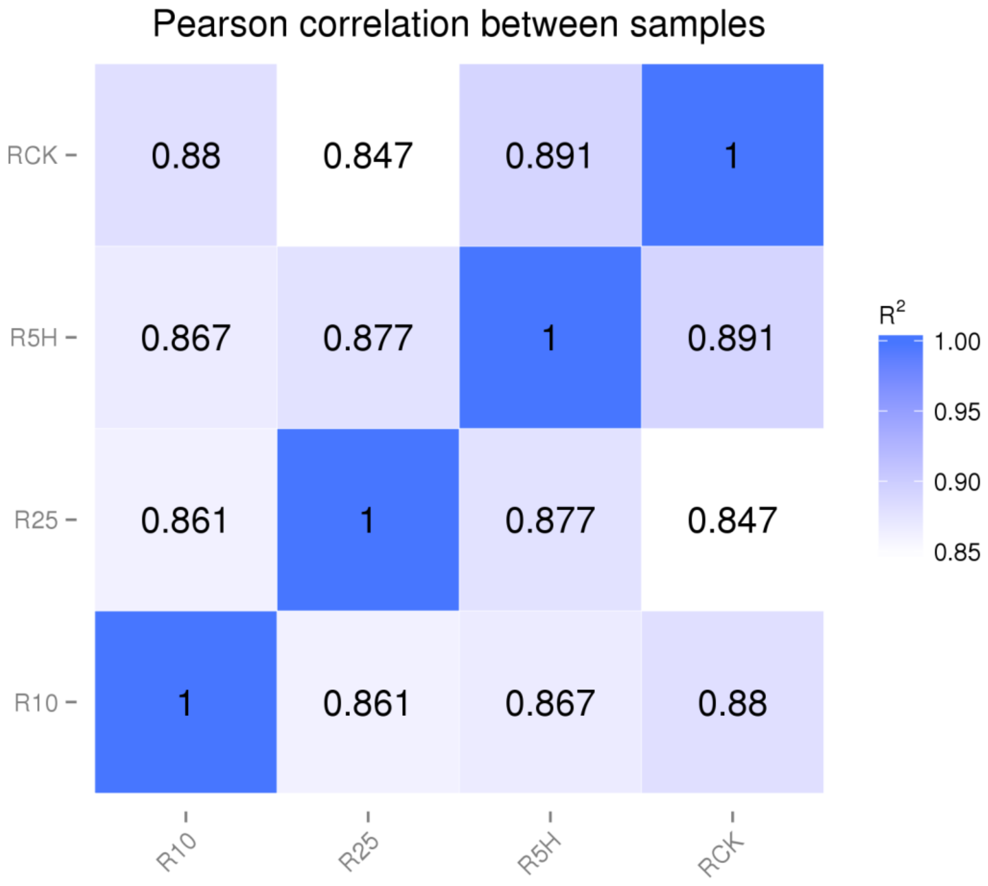

Supplemental data S4 Pearson correlation analysis result among RCK, R5H, R10 and R25.

Supplemental data S5 Predicted target genes of the differentially expressed （DE）miRNAs

geneID	miRNA	InterPro description
GSMUA_Achr10G00250_00	mac-miR169a-3p	Importin-beta, N-terminal||Armadillo-like helical||Armadillo-type fold
GSMUA_Achr10G00320_00	mac-miR529-5p,mac-miR529e,mac-miR529b	HAD-like domain
GSMUA_Achr10G01880_00	mac-miR5658	NHL repeat||Six-bladed beta-propeller, TolB-like
GSMUA_Achr10G02390_00	mac-miR396f-5p	Zinc finger, C2H2||Zinc finger, C2H2-like
GSMUA_Achr10G02910_00	mac-miR169h-3p	Ribosomal protein L11, C-terminal||Ribosomal protein L11, conserved site||Ribosomal protein L11||Ribosomal protein L11, N-terminal
GSMUA_Achr10G02970_00	mac-miR156a.1,mac-miR156k,mac-miR156h,mac-mi	Transcription factor, SBP-box
GSMUA_Achr10G03230_00	mac-miR172c-3p,mac-miR172d,mac-miR172e-3p.2,m	AP2/ERF domain||DNA-binding, integrase-type||AP2-like ethylene-responsive transcription factor
GSMUA_Achr10G03540_00	mac-miR535d,mac-nmiR5	Na+/H+ exchanger||Cation/H+ exchanger||Cation/H+ exchanger, CPA1 family
GSMUA_Achr10G03800_00	mac-miR5658	EF-hand-like domain||Riboflavin synthase-like beta-barrel||NADPH oxidase Respiratory burst||Cytochrome b245, heavy chain||Ferredoxin reductase-type FAD-binding domain||EF-Hand 1, calcium-binding site||Ferric reductase, NAD binding||Ferric reductase transmembrane component-like domain||FAD-binding 8||Calcium-binding EF-hand
GSMUA_Achr10G04030_00	mac-miR169c-3p	Protein of unknown function DUF1313
GSMUA_Achr10G04600_00	mac-miR160h,mac-miR160e-5p,mac-miR160b.2,mac-	Auxin response factor||B3 DNA binding domain||DNA-binding pseudobarrel domain
GSMUA_Achr10G05870_00	mac-miR5658	FGFR1 oncogene partner (FOP), N-terminal dimerisation domain||LisH dimerisation motif
GSMUA_Achr10G05880_00	mac-miR164b	Nucleotide-binding, alpha-beta plait||Zinc finger, RING/FYVE/PHD-type||RNA recognition motif domain, eukaryote||Zinc finger, RING-type
GSMUA_Achr10G05890_00	mac-miR319a-5p	Zinc finger, Dof-type
GSMUA_Achr10G05930_00	mac-miR169a-3p	P-loop containing nucleoside triphosphate hydrolase||ATPase, AAA-type, core||Double Clp-N motif
GSMUA_Achr10G06250_00	mac-miR156h-3p	Ribosomal protein L30, conserved site||Ribosomal protein L30, N-terminal||Ribosomal protein L7, eukaryotic||Ribosomal protein L30, ferredoxin-like fold domain
GSMUA_Achr10G06370_00	mac-miR396f-5p,mac-miR396a.1,mac-miR396e-5p,m	Growth-regulating factor||Glutamine-Leucine-Glutamine, QLQ||WRC
GSMUA_Achr10G07770_00	mac-miR156k-5p	Pentatricopeptide repeat
GSMUA_Achr10G08050_00	mac-miR172j	Protein of unknown function DUF4535||Dihydrolipoamide acetyltransferase, long form||GroEL-like equatorial domain||2-oxo acid dehydrogenase, lipoyl-binding site||Biotin/lipoyl attachment||2-oxoacid dehydrogenase acyltransferase, catalytic domain||Single hybrid motif||Chloramphenicol acetyltransferase-like domain||E3 binding
GSMUA_Achr10G08140_00	mac-miR156b.2	Tetratricopeptide repeat-containing domain||Clustered mitochondria protein, N-terminal||Tetratricopeptide-like helical||Tetratricopeptide repeat
GSMUA_Achr10G08440_00	mac-miR5368	Armadillo-like helical||Cell differentiation, Rcd1-like||Armadillo-type fold
GSMUA_Achr10G09020_00	mac-miR530	Protein of unknown function DUF869, plant
GSMUA_Achr10G09210_00	mac-miR160a-3p	Peptidase S8, subtilisin-related||Peptidase S8, subtilisin, Ser-active site||Peptidase S8/S53, subtilisin/kexin/sedolisin||Proteinase inhibitor I9||Protease-associated domain, PA
GSMUA_Achr10G10180_00	mac-miR393a.1,mac-miR393a.2,mac-miR393a-5p,ma	Nucleobase-ascorbate transporter 11/12||Xanthine/uracil/vitamin C permease
GSMUA_Achr10G10440_00	mac-miR408-3p.3	Basic-leucine zipper domain
GSMUA_Achr10G11270_00	mac-miR408d,mac-miR408-3p.3,mac-miR408b,mac-r	Blue (type 1) copper protein, binding site||Plastocyanin-like||Cupredoxin
GSMUA_Achr10G12000_00	mac-miR393a.1,mac-miR393a.2,mac-miR393-5p	Leucine-rich repeat, cysteine-containing subtype
GSMUA_Achr10G12980_00	mac-miR169a-3p	Transcription factor IIIB subunit Brf1||Brf1-like TBP-binding||Transcription factor TFIIB
GSMUA_Achr10G14200_00	mac-miR169h-3p,mac-miR169c-3p	Ribosomal protein L7Ae/L30e/S12e/Gadd45||50S ribosomal protein L30e-like||Ribosomal protein L7Ae/L8/Nhp2 family||Ribosomal protein L7A/L8||Ribosomal protein L7Ae conserved site
GSMUA_Achr10G15560_00	mac-miR171k,mac-miR171b-3p.3,mac-miR171f.2,ma	IQ motif, EF-hand binding site
GSMUA_Achr10G15640_00	mac-miR166u	Glycoside hydrolase, superfamily||Glycoside hydrolase, family 17||Glycoside hydrolase, catalytic domain
GSMUA_Achr10G15980_00	mac-miR444f	WD40 repeat||WD40/YVTN repeat-like-containing domain||WD40-repeat-containing domain
GSMUA_Achr10G16120_00	mac-miR171a-3p.2,mac-miR171a-3p.1,mac-miR171a.	LIM-domain binding protein/SEUSS
GSMUA_Achr10G17120_00	mac-miR156a-5p,mac-miR156b.1,mac-miR156f-5p	Armadillo-type fold||U box domain||Zinc finger, RING/FYVE/PHD-type||Armadillo-like helical||Armadillo
GSMUA_Achr10G17860_00	mac-miR5234	Protein of unknown function DUF1423, plant
GSMUA_Achr10G17950_00	mac-miR530a.1,mac-miR530a.2,mac-miR530-5p	Myosin II heavy chain-like
GSMUA_Achr10G18020_00	mac-miR396a.4,mac-miR396a-5p,mac-miR396b,mac-	Sel1-like||Tetratricopeptide-like helical
GSMUA_Achr10G18040_00	mac-miR408b,mac-miR408-3p.2,mac-miR408d,mac-r	Plastocyanin-like||Cupredoxin
GSMUA_Achr10G19000_00	mac-miR396h,mac-miR396a.3,mac-miR396a.2,mac-n	ClpP/crotonase-like domain||Carbamoyl-phosphate synthetase large subunit-like, ATP-binding domain||Acetyl-coenzyme A carboxyltransferase, C-terminal||ATP-grasp fold, subdomain 2||ATP-grasp fold, subdomain 1||Acetyl-CoA carboxylase, central domain||Biotin carboxylase, C-terminal||Rudiment single hybrid motif||Biotin carboxylation domain||Pre-ATP-grasp fold||Biotin/lipoyl attachment||Single hybrid motif||Carboxyl transferase||ATP-grasp fold||Carbamoyl-phosphate synthase, large subunit, N-terminal||Acetyl-coenzyme A carboxyltransferase, N-terminal
GSMUA_Achr10G19120_00	mac-miR5658	Ubiquitin carboxyl-terminal hydrolase-like domain||Peptidase C19, ubiquitin carboxyl-terminal hydrolase 2, conserved site||Peptidase C19, ubiquitin carboxyl-terminal hydrolase 2
GSMUA_Achr10G20370_00	mac-miR396f,mac-miR396g-5p.1	FAD binding domain||Fumarate reductase/succinate dehydrogenase flavoprotein-like, C-terminal||Succinate dehydrogenase/fumarate reductase flavoprotein, catalytic domain||L-aspartate oxidase
GSMUA_Achr10G20720_00	mac-miR397.3	Basic-leucine zipper domain
GSMUA_Achr10G21360_00	mac-miR394a	NUDIX hydrolase domain-like||NUDIX hydrolase domain||NUDIX hydrolase, conserved site||Nudix hydrolase 6-like
GSMUA_Achr10G22350_00	mac-miR164a.1,mac-nmiR21,mac-nmiR3,mac-miR16	No apical meristem (NAM) protein
GSMUA_Achr10G22610_00	mac-miR5139	Transcription factor GRAS
GSMUA_Achr10G22640_00	mac-miR5658	Armadillo-like helical||Clathrin/coatomer adaptor, adaptin-like, N-terminal||AP complex subunit beta||Armadillo-type fold||AP-3 complex subunit beta
GSMUA_Achr10G23010_00	mac-miR156a.2,mac-miR156b.2	EF-hand-like domain||Phospholipase C, phosphoinositol-specific, EF-hand-like
GSMUA_Achr10G23280_00	mac-miR156k-5p,mac-miR156g.2,mac-miR157a-5p,m	Transcription factor, SBP-box
GSMUA_Achr10G23440_00	mac-miR166a	Homeobox, conserved site||Leucine zipper, homeobox-associated||Homeodomain||Homeodomain-like
GSMUA_Achr10G24770_00	mac-miR156k,mac-miR156g.2,mac-miR156h,mac-mi	Protein of unknown function DUF3550/UPF0682
GSMUA_Achr10G24920_00	mac-miR396g-3p	NB-ARC||P-loop containing nucleoside triphosphate hydrolase
GSMUA_Achr10G25270_00	mac-miR172c-3p	AP2/ERF domain||DNA-binding, integrase-type
GSMUA_Achr10G25490_00	mac-miR827	Lipid phosphate phosphatase, plant||Phosphatidic acid phosphatase type 2/haloperoxidase
GSMUA_Achr10G25680_00	mac-miR396g-5p.1,mac-miR396b,mac-miR396,mac-r	Leucine-rich repeat||Leucine-rich repeat, typical subtype
GSMUA_Achr10G25890_00	mac-miR396h,mac-miR396b,mac-miR396a-5p	GAGA binding-like
GSMUA_Achr10G26180_00	mac-miR164a.1,mac-nmiR21,mac-miR164d,mac-miR	No apical meristem (NAM) protein
GSMUA_Achr10G26290_00	mac-miR5658	Pleckstrin-like, plant||Domain of unknown function DUF828
GSMUA_Achr10G26310_00	mac-miR156a.1,mac-miR156h	Phox/Bem1p||AUX/IAA protein
GSMUA_Achr10G26450_00	mac-miR171b.2,mac-miR171a.2,mac-miR159a-3p	Aluminum-activated malate transporter
GSMUA_Achr10G26610_00	mac-miR159a.3,mac-miR159c.3,mac-miR159a-3p	Homeodomain-like||SANT/Myb domain||Myb domain
GSMUA_Achr10G26770_00	mac-miR5658	Heavy metal-associated domain, HMA
GSMUA_Achr10G27090_00	mac-miR398a-3p.2,mac-miR398b	Cytochrome c oxidase, subunit Vb
GSMUA_Achr10G27120_00	mac-miR394a	Agenet-like domain||Tudor-like, plant
GSMUA_Achr10G27470_00	mac-miR169a-3p,mac-miR169c-3p	Glyoxalase/fosfomycin resistance/dioxygenase domain||Glyoxalase/Bleomycin resistance protein/Dihydroxybiphenyl dioxygenase
GSMUA_Achr10G27550_00	mac-miR6300	IQ motif, EF-hand binding site
GSMUA_Achr10G27830_00	mac-miR395b.2,mac-miR395g	Galactose-binding domain-like||NADH:ubiquinone oxidoreductase intermediate-associated protein 30||NAD(P)-binding domain
GSMUA_Achr10G28310_00	mac-miR408-5p	Syntaxin, N-terminal||t-SNARE||Target SNARE coiled-coil domain||Syntaxin/epimorphin, conserved site
GSMUA_Achr10G28340_00	mac-miR169c-3p	Pentatricopeptide repeat||Tetratricopeptide-like helical
GSMUA_Achr10G28470_00	mac-miR394a	EMSY N-terminal||Agenet-like domain||Tudor-like, plant
GSMUA_Achr10G29170_00	mac-miR390e	Serine/threonine-protein kinase, active site||Protein kinase, catalytic domain||Protein kinase-like domain||Serine-threonine/tyrosine-protein kinase catalytic domain||Protein kinase, ATP binding site
GSMUA_Achr10G29280_00	mac-miR159a-3p,mac-miR319,mac-miR319q,mac-mi	Homeodomain-like||SANT/Myb domain||Myb domain
GSMUA_Achr10G29410_00	mac-miR167c.2,mac-miR167d.2	ATPase, AAA-type, core||P-loop containing nucleoside triphosphate hydrolase
GSMUA_Achr10G29780_00	mac-miR162-3p	Ribosomal protein L34Ae||Protein of unknown function DUF1666
GSMUA_Achr10G29970_00	mac-miR5658	Myb domain||SANT/Myb domain||Homeodomain-like
GSMUA_Achr10G30040_00	mac-miR444a-3p.2,mac-miR444f	Transcription factor, MADS-box
GSMUA_Achr10G31170_00	mac-miR164a.1,mac-miR164b,mac-miR164c	BRCT domain
GSMUA_Achr11G00590_00	mac-miR5658	Aquaporin-like||Major intrinsic protein||Major intrinsic protein, conserved site
GSMUA_Achr11G01610_00	mac-miR167.1,mac-miR167c-5p,mac-miR167a-5p,ma	AUX/IAA protein||DNA-binding pseudobarrel domain||B3 DNA binding domain||Auxin response factor||Phox/Bem1p
GSMUA_Achr11G01720_00	mac-miR167c.1	Oligopeptide transporter OPT superfamily
GSMUA_Achr11G01740_00	mac-miR395b.2,mac-miR395b-3p	Peptidoglycan-binding lysin domain
GSMUA_Achr11G01870_00	mac-miR396g,mac-miR396a.1	Protein of unknown function DUF789
GSMUA_Achr11G02040_00	mac-miR169c-3p	JmjC domain||Transcription factor jumonji, JmjN||Zinc finger, C2H2-like||Zinc finger C2H2-type/integrase DNA-binding domain||Zinc finger, C2H2
GSMUA_Achr11G02150_00	mac-miR5658	Zinc finger, C2H2
GSMUA_Achr11G02500_00	mac-miR5658	Gnk2-homologous domain
GSMUA_Achr11G02620_00	mac-miR172j,mac-miR172a-5p	Tetratricopeptide repeat-containing domain||Tetratricopeptide repeat||Tetratricopeptide TPR2||Tetratricopeptide-like helical
GSMUA_Achr11G03930_00	mac-miR164	Peptidase S8, subtilisin-related||Peptidase S8/S53, subtilisin/kexin/sedolisin||Proteinase inhibitor I9
GSMUA_Achr11G04360_00	mac-miR171a.1,mac-miR171i-3p,mac-miR171m,mac-	Transcription factor GRAS
GSMUA_Achr11G04450_00	mac-miR166a,mac-miR166h-3p	CCR4-Not complex, Not1 subunit, domain of unknown function DUF3819||CCR4-Not complex component, Not1, C-terminal
GSMUA_Achr11G05040_00	mac-miR395a.1	Immunoglobulin-like fold||Carboxypeptidase-like, regulatory domain||Carboxypeptidase, regulatory domain||Collagen-binding surface protein Cna, B-type domain
GSMUA_Achr11G05620_00	mac-miR6300	RFT1
GSMUA_Achr11G06110_00	mac-miR397.1	Armadillo-type fold||Armadillo-like helical
GSMUA_Achr11G06550_00	mac-miR166i.2	Alpha-ketoglutarate-dependent dioxygenase AlkB-like||Oxoglutarate/iron-dependent dioxygenase
GSMUA_Achr11G07410_00	mac-miR395b-3p	EEIG1/EHBP1 N-terminal domain
GSMUA_Achr11G08130_00	mac-miR156h-3p	Ribosomal protein L30, conserved site||Ribosomal protein L30, N-terminal||Ribosomal protein L7, eukaryotic||Ribosomal protein L30, ferredoxin-like fold domain
GSMUA_Achr11G08190_00	mac-miR172j	Small GTPase superfamily, ARF/SAR type||Small GTPase superfamily, ARF type||P-loop containing nucleoside triphosphate hydrolase||Small GTPase superfamily, Rab type||Small GTPase superfamily, SAR1-type||Small GTP-binding protein domain
GSMUA_Achr11G08860_00	mac-miR169c-3p	Ribokinase-like||Ribokinase||PfkB||Carbohydrate/puine kinase, PfkB, conserved site
GSMUA_Achr11G08880_00	mac-miR396g-5p.2	Rossmann-like alpha/beta/alpha sandwich fold||Tryptophan-tRNA ligase||Aminoacyl-tRNA synthetase, class Ic
GSMUA_Achr11G09200_00	mac-miR393a.1	Pyridoxal phosphate-dependent transferase, major region, subdomain 2||Aminotransferase, class I/classII||Pyridoxal phosphate-dependent transferase, major region, subdomain 1||Pyridoxal phosphate-dependent transferase, major domain
[illegible]	[illegible]	[illegible]

Supplemental data S6 GO enrichment result of the predicted target genes of differentially expressed (DE) miRNAs

GO accession	Description	Term type	Over represented pValue	Corrected pValue	DEG item	DEG list	Bg item	Bg list	Gene IDs
GO:0003677	DNA bindi	molecular_	1.08E-28	6.21E-25	132	422	2629	23090	[illegible]
GO:0005634	nucleus	cellular_cc	2.97E-26	8.57E-23	114	422	2159	23090	[illegible]
GO:0003676	nucleic aci	molecular_	4.80E-17	9.22E-14	141	422	3913	23090	[illegible]
GO:0005507	copper ion	molecular_	2.80E-16	4.04E-13	25	422	163	23090	[illegible]
GO:0006355	regulation	biological_	9.08E-16	7.47E-13	90	422	2025	23090	[illegible]
GO:1903506	regulation	biological_	9.08E-16	7.47E-13	90	422	2025	23090	[illegible]
GO:2001141	regulation	biological_	9.08E-16	7.47E-13	90	422	2025	23090	[illegible]
GO:0051252	regulation	biological_	1.18E-15	8.52E-13	90	422	2034	23090	[illegible]
GO:0043227	membrane	cellular_cc	3.22E-15	1.86E-12	132	422	3730	23090	[illegible]
GO:0043231	intracellul	cellular_cc	3.22E-15	1.86E-12	132	422	3730	23090	[illegible]
GO:0019219	regulation	biological_	4.85E-15	2.54E-12	90	422	2083	23090	[illegible]
GO:0006351	transcripti	biological_	6.38E-15	2.83E-12	93	422	2204	23090	[illegible]
GO:0097659	nucleic aci	biological_	6.38E-15	2.83E-12	93	422	2204	23090	[illegible]
GO:0032774	RNA biosyn	biological_	6.92E-15	2.83E-12	93	422	2207	23090	[illegible]
GO:0010556	regulation	biological_	8.04E-15	2.89E-12	90	422	2101	23090	[illegible]
GO:2000112	regulation	biological_	8.04E-15	2.89E-12	90	422	2101	23090	[illegible]
GO:0031326	regulation	biological_	9.49E-15	3.22E-12	90	422	2107	23090	[illegible]
GO:0009889	regulation	biological_	1.12E-14	3.59E-12	90	422	2113	23090	[illegible]
GO:0010468	regulation	biological_	1.47E-14	4.45E-12	91	422	2160	23090	[illegible]
GO:0051171	regulation	biological_	1.69E-14	4.87E-12	90	422	2128	23090	[illegible]
GO:0009734	auxin-acti	biological_	1.22E-13	3.20E-11	19	422	110	23090	[illegible]
GO:0071365	cellular res	biological_	1.22E-13	3.20E-11	19	422	110	23090	[illegible]
GO:0080090	regulation	biological_	2.32E-13	5.72E-11	90	422	2228	23090	[illegible]
GO:0031323	regulation	biological_	2.38E-13	5.72E-11	90	422	2229	23090	[illegible]
GO:0019222	regulation	biological_	2.52E-13	5.81E-11	91	422	2502	23090	[illegible]
GO:0060255	regulation	biological_	3.44E-13	7.62E-11	91	422	2282	23090	[illegible]
GO:0034654	nucleobase	biological_	5.28E-13	1.13E-10	95	422	2455	23090	[illegible]
GO:0009733	response t	biological_	2.33E-12	4.79E-10	19	422	129	23090	[illegible]
GO:0018130	heterocycl	biological_	1.07E-11	2.11E-09	96	422	2630	23090	[illegible]
GO:0043229	intracellul	cellular_cc	1.78E-11	3.36E-09	141	422	4597	23090	[illegible]
GO:0043226	organelle	cellular_cc	1.80E-11	3.36E-09	141	422	4598	23090	[illegible]
GO:0019438	aromatic c	biological_	2.57E-11	4.63E-09	96	422	2672	23090	[illegible]
GO:1901362	organic cyc	biological_	5.53E-11	9.66E-09	97	422	2751	23090	[illegible]
GO:0009755	hormone-m	biological_	6.85E-10	1.16E-07	19	422	177	23090	[illegible]
GO:0032870	cellular res	biological_	7.54E-10	1.24E-07	19	422	178	23090	[illegible]
GO:0071495	cellular res	biological_	8.44E-10	1.35E-07	19	422	179	23090	[illegible]
GO:0016070	RNA metab	biological_	1.31E-09	2.04E-07	95	422	2836	23090	[illegible]
GO:0050794	regulation	biological_	6.44E-09	9.77E-07	98	422	3058	23090	[illegible]
GO:0050789	regulation	biological_	1.15E-08	1.69E-06	105	422	3401	23090	[illegible]
GO:0009725	response t	biological_	1.20E-08	1.72E-06	20	422	233	23090	[illegible]
GO:0044424	intracellul	cellular_cc	1.72E-08	2.42E-06	151	422	5565	23090	[illegible]
GO:0009719	response t	biological_	2.33E-08	3.19E-06	20	422	242	23090	[illegible]
GO:0065007	biological r	biological_	4.01E-08	5.37E-06	106	422	3531	23090	[illegible]
GO:0044271	cellular nit	biological_	4.79E-08	6.27E-06	101	422	3320	23090	[illegible]
GO:0090304	nucleic aci	biological_	7.35E-08	9.41E-06	97	422	3172	23090	[illegible]
GO:0046271	phenylprop	biological_	1.10E-07	1.32E-05	4	422	4	23090	[illegible]
GO:0046274	lignin cata	biological_	1.10E-07	1.32E-05	4	422	4	23090	[illegible]
GO:0052716	hydroquino	molecular_	1.10E-07	1.32E-05	4	422	4	23090	[illegible]
[illegible]	[illegible]	[illegible]	[illegible]	[illegible]	[illegible]	[illegible]	[illegible]	[illegible]	[illegible]

GO_accession	Description	Term_type	Over_repre	Corrected_p	DEG_item	DEG_list	Bg_item	Bg_list	Gene_IDs
GO:00036	DNA bindi	molecular_	3.10E-28	1.78E-24	130	416	2629	23090	GSMUA_Achr6G30050_001,GSMUA_Achr8G25550_001,GSMUA_Achr10G02970_001,GSMUA_Achr11G23500_001,GSMUA_Achr5G26580_001,GSMUA_Achr6G34420_001, [illegible]
GO:00056	nucleus	cellular_co	1.11E-25	3.20E-22	112	416	2159	23090	GSMUA_Achr4G10670_001,GSMUA_Achr3G23290_001,GSMUA_Achr5G14630_001,GSMUA_Achr4G01450_001,GSMUA_AchrUn_randomG08380_001, [illegible]
GO:00036	nucleic aci	molecular_	1.80E-18	3.46E-15	143	416	3913	23090	GSMUA_Achr11G25770_001,GSMUA_Achr6G36930_001,GSMUA_Achr5G13440_001,GSMUA_Achr8G24960_001,GSMUA_Achr5G25090_001,GSMUA_Achr6G19850_001, [illegible]
GO:00432	membrane-	cellular_co	5.94E-17	6.85E-14	135	416	3730	23090	GSMUA_Achr9G06850_001,GSMUA_Achr5G07520_001,GSMUA_Achr4G25960_001,GSMUA_Achr9G14160_001,GSMUA_Achr5G08780_001,GSMUA_Achr3G05360_001, [illegible]
GO:00432	intracellula	cellular_co	5.94E-17	6.85E-14	135	416	3730	23090	GSMUA_Achr10G02970_001,GSMUA_Achr6G34420_001,GSMUA_Achr5G26580_001,GSMUA_Achr8G25550_001,GSMUA_Achr6G30050_001,GSMUA_Achr6G36010_001, [illegible]
GO:00097	auxin-activ	biological_	9.47E-14	7.80E-11	19	416	110	23090	GSMUA_Achr11G01610_001,GSMUA_Achr10G26310_001,GSMUA_Achr5G00590_001,GSMUA_Achr8G18930_001,GSMUA_Achr5G02450_001,GSMUA_Achr11G25770_001, [illegible]
GO:00713	cellular res	biological_	9.47E-14	7.80E-11	19	416	110	23090	GSMUA_Achr5G18540_001,GSMUA_Achr10G04600_001,GSMUA_Achr5G14630_001,GSMUA_Achr3G23290_001,GSMUA_AchrUn_randomG06470_001, [illegible]
GO:00063	regulation	biological_	5.17E-13	2.98E-10	83	416	2025	23090	GSMUA_Achr11G01610_001,GSMUA_Achr2G09580_001,GSMUA_Achr4G12860_001,GSMUA_Achr8G21030_001,GSMUA_Achr8G18930_001,GSMUA_Achr5G02450_001, [illegible]
GO:19035	regulation	biological_	5.17E-13	2.98E-10	83	416	2025	23090	GSMUA_Achr5G14630_001,GSMUA_Achr3G23290_001,GSMUA_Achr8G33310_001,GSMUA_Achr4G07190_001,GSMUA_Achr3G08080_001,GSMUA_Achr6G31350_001, [illegible]
GO:20011	regulation	biological_	5.17E-13	2.98E-10	83	416	2025	23090	GSMUA_Achr10G04600_001,GSMUA_Achr8G18550_001,GSMUA_Achr2G21430_001,GSMUA_Achr8G28560_001,GSMUA_Achr10G03230_001,GSMUA_Achr2G17590_001, [illegible]
GO:00512	regulation	biological_	6.52E-13	3.42E-10	83	416	2034	23090	GSMUA_Achr3G23360_001,GSMUA_Achr4G18240_001,GSMUA_Achr5G07270_001,GSMUA_Achr11G13590_001,GSMUA_Achr3G29970_001,GSMUA_Achr5G26640_001, [illegible]
GO:00192	regulation	biological_	8.60E-13	3.82E-10	84	416	2083	23090	GSMUA_Achr1G05940_001,GSMUA_Achr5G00590_001,GSMUA_Achr10G26180_001,GSMUA_Achr11G25770_001,GSMUA_Achr11G04360_001,GSMUA_Achr6G19850_001, [illegible]
GO:00104	regulation	biological_	8.61E-13	3.82E-10	86	416	2160	23090	GSMUA_Achr4G18450_001,GSMUA_Achr10G26310_001,GSMUA_Achr11G17950_001,GSMUA_Achr11G21080_001,GSMUA_Achr8G24880_001,GSMUA_Achr1G02000_001, [illegible]
GO:00511	regulation	biological_	1.01E-12	4.17E-10	85	416	2128	23090	GSMUA_Achr9G10210_001,GSMUA_Achr6G19850_001,GSMUA_Achr10G22350_001,GSMUA_Achr5G18530_001,GSMUA_Achr5G00590_001,GSMUA_Achr1G05940_001, [illegible]
GO:00105	regulation	biological_	1.35E-12	4.86E-10	84	416	2101	23090	GSMUA_Achr5G26640_001,GSMUA_Achr3G29970_001,GSMUA_Achr11G13590_001,GSMUA_Achr5G07270_001,GSMUA_Achr4G18240_001,GSMUA_Achr3G23360_001, [illegible]
GO:20001	regulation	biological_	1.35E-12	4.86E-10	84	416	2101	23090	GSMUA_Achr3G23360_001,GSMUA_Achr11G13590_001,GSMUA_Achr5G07270_001,GSMUA_Achr4G18240_001,GSMUA_Achr5G26640_001,GSMUA_Achr3G29970_001, [illegible]
GO:00313	regulation	biological_	1.57E-12	5.31E-10	84	416	2107	23090	GSMUA_Achr10G22610_001,GSMUA_Achr4G06100_001,GSMUA_Achr5G18540_001,GSMUA_Achr5G20860_001,GSMUA_Achr5G06550_001,GSMUA_Achr5G26640_001, [illegible]
GO:00097	response to	biological_	1.81E-12	5.51E-10	19	416	129	23090	GSMUA_Achr5G18540_001,GSMUA_Achr10G04600_001,GSMUA_Achr5G14630_001,GSMUA_AchrUn_randomG06470_001,GSMUA_Achr3G23290_001, [illegible]
GO:00098	regulation	biological_	1.82E-12	5.51E-10	84	416	2113	23090	GSMUA_Achr8G30150_001,GSMUA_Achr1G02000_001,GSMUA_Achr8G13620_001,GSMUA_Achr11G21080_001,GSMUA_Achr8G24880_001,GSMUA_Achr10G26310_001, [illegible]
GO:00063	transcriptio	biological_	2.51E-12	6.89E-10	86	416	2204	23090	GSMUA_Achr5G00500_001,GSMUA_AchrUn_randomG04680_001,GSMUA_Achr11G25720_001,GSMUA_Achr9G29480_001,GSMUA_Achr11G23870_001, [illegible]
GO:00976	nucleic aci	biological_	2.51E-12	6.89E-10	86	416	2204	23090	GSMUA_Achr10G04600_001,GSMUA_Achr8G18550_001,GSMUA_Achr2G21430_001,GSMUA_Achr10G03230_001,GSMUA_Achr8G28560_001,GSMUA_Achr2G17590_001, [illegible]
GO:00327	RNA biosy	biological_	2.70E-12	7.06E-10	86	416	2207	23090	GSMUA_Achr5G06550_001,GSMUA_Achr5G20860_001,GSMUA_Achr5G18540_001,GSMUA_Achr4G06100_001,GSMUA_Achr10G22610_001,GSMUA_Achr3G23360_001, [illegible]
GO:00346	nucleobase	biological_	3.43E-12	8.60E-10	92	416	2455	23090	GSMUA_AchrUn_randomG12610_001,GSMUA_Achr9G04270_001,GSMUA_Achr4G11090_001,GSMUA_Achr9G03060_001,GSMUA_AchrUn_randomG16200_001, [illegible]
GO:00602	regulation	biological_	6.20E-12	1.49E-09	87	416	2282	23090	GSMUA_Achr5G26490_001,GSMUA_Achr5G14630_001,GSMUA_Achr3G23290_001,GSMUA_Achr8G33310_001,GSMUA_Achr4G07190_001,GSMUA_Achr3G08080_001, [illegible]
GO:00181	heterocycl	biological_	1.09E-11	2.41E-09	95	416	2630	23090	GSMUA_Achr11G04360_001,GSMUA_Achr11G25770_001,GSMUA_Achr10G26180_001,GSMUA_Achr1G05940_001,GSMUA_Achr5G00590_001,GSMUA_Achr5G18530_001, [illegible]
GO:00800	regulation	biological_	1.10E-11	2.41E-09	85	416	2228	23090	GSMUA_Achr4G07190_001,GSMUA_Achr8G33310_001,GSMUA_Achr3G08080_001,GSMUA_Achr5G14630_001,GSMUA_Achr5G26490_001,GSMUA_Achr3G23290_001, [illegible]
GO:00313	regulation	biological_	1.13E-11	2.41E-09	85	416	2229	23090	GSMUA_Achr9G27530_001,GSMUA_AchrUn_randomG06470_001,GSMUA_Achr10G25270_001,GSMUA_Achr8G20090_001,GSMUA_Achr6G33140_001, [illegible]
GO:00192	regulation	biological_	2.30E-11	4.73E-09	91	416	2502	23090	GSMUA_Achr10G22350_001,GSMUA_Achr6G19850_001,GSMUA_Achr9G10210_001,GSMUA_Achr5G18530_001,GSMUA_Achr10G26180_001,GSMUA_Achr1G05940_001, [illegible]
GO:00194	aromatic c	biological_	2.61E-11	5.18E-09	95	416	2672	23090	GSMUA_Achr10G26180_001,GSMUA_Achr5G00590_001,GSMUA_Achr1G05940_001,GSMUA_Achr11G04360_001,GSMUA_Achr11G25770_001,GSMUA_Achr10G22350_001, [illegible]
GO:00432	intracellula	cellular_co	1.01E-10	1.90E-08	137	416	4597	23090	GSMUA_AchrUn_randomG05850_001,GSMUA_Achr4G24900_001,GSMUA_Achr9G23140_001,GSMUA_Achr9G20220_001,GSMUA_Achr7G12850_001,GSMUA_Achr6G05690_001, [illegible]
GO:00432	organelle	cellular_co	1.02E-10	1.90E-08	137	416	4598	23090	GSMUA_Achr10G22350_001,GSMUA_Achr8G23820_001,GSMUA_Achr6G19850_001,GSMUA_Achr9G10210_001,GSMUA_Achr5G25090_001,GSMUA_Achr8G24960_001, [illegible]
GO:19013	organic cy	biological_	1.24E-10	2.24E-08	95	416	2751	23090	GSMUA_Achr8G20090_001,GSMUA_Achr6G27140_001,GSMUA_Achr10G25270_001,GSMUA_Achr9G27530_001,GSMUA_AchrUn_randomG06470_001, [illegible]
GO:00097	hormone-m	biological_	5.40E-10	9.43E-08	19	416	177	23090	GSMUA_Achr5G18540_001,GSMUA_Achr10G04600_001,GSMUA_Achr5G14630_001,GSMUA_AchrUn_randomG06470_001,GSMUA_Achr3G23290_001, [illegible]
GO:00328	cellular res	biological_	5.94E-10	1.01E-07	19	416	178	23090	GSMUA_Achr4G18240_001,GSMUA_Achr5G03960_001,GSMUA_Achr9G29480_001,GSMUA_Achr10G04600_001,GSMUA_Achr5G18540_001,GSMUA_Achr3G23290_001, [illegible]
GO:00714	cellular res	biological_	6.54E-10	1.08E-07	19	416	179	23090	GSMUA_Achr5G14630_001,GSMUA_AchrUn_randomG06470_001,GSMUA_Achr3G23290_001,GSMUA_Achr5G18540_001,GSMUA_Achr10G04600_001, [illegible]
GO:00055	copper ion	molecular_	9.89E-10	1.58E-07	18	416	163	23090	GSMUA_Achr3G15320_001,GSMUA_Achr9G03700_001,GSMUA_Achr9G03380_001,GSMUA_Achr3G09960_001,GSMUA_Achr6G33880_001,GSMUA_Achr4G24900_001, [illegible]
GO:00444	intracellula	cellular_co	3.32E-09	5.18E-07	152	416	5565	23090	GSMUA_Achr5G26490_001,GSMUA_Achr3G23290_001,GSMUA_Achr6G29790_001,GSMUA_Achr8G33310_001,GSMUA_Achr4G04300_001,GSMUA_Achr3G08080_001, [illegible]
GO:00160	RNA metal	biological_	5.75E-09	8.73E-07	92	416	2836	23090	GSMUA_Achr5G03960_001,GSMUA_Achr3G07370_001,GSMUA_Achr1G09830_001,GSMUA_Achr6G33140_001,GSMUA_Achr5G25100_001,GSMUA_Achr9G27530_001, [illegible]
GO:00903	nucleic aci	biological_	9.37E-09	1.36E-06	99	416	3172	23090	GSMUA_Achr8G18930_001,GSMUA_Achr5G02450_001,GSMUA_Achr4G12860_001,GSMUA_Achr2G09580_001,GSMUA_Achr9G12650_001,GSMUA_Achr8G21030_001, [illegible]
GO:00097	response to	biological_	9.42E-09	1.36E-06	20	416	233	23090	GSMUA_Achr5G02450_001,GSMUA_Achr8G18930_001,GSMUA_Achr6G18900_001,GSMUA_Achr8G13620_001,GSMUA_Achr11G25770_001,GSMUA_Achr10G26310_001, [illegible]
GO:00097	response to	biological_	1.84E-08	2.58E-06	20	416	242	23090	GSMUA_Achr8G18930_001,GSMUA_Achr5G02450_001,GSMUA_Achr8G13620_001,GSMUA_Achr6G18900_001,GSMUA_Achr11G25770_001,GSMUA_Achr11G01610_001, [illegible]
GO:00056	intracellula	cellular_co	5.30E-08	7.27E-06	154	416	5898	23090	GSMUA_Achr5G18530_001,GSMUA_Achr11G16520_001,GSMUA_Achr6G22280_001,GSMUA_Achr9G22260_001,GSMUA_Achr5G00590_001,GSMUA_Achr10G26180_001, [illegible]
GO:00067	cellular aro	biological_	9.06E-08	1.21E-05	113	416	3968	23090	GSMUA_Achr6G33140_001,GSMUA_AchrUn_randomG15230_001,GSMUA_Achr5G03960_001,GSMUA_Achr1G09830_001,GSMUA_Achr3G07370_001,GSMUA_Achr5G25100_001, [illegible]
GO:00462	phenylprop	biological_	1.04E-07	1.30E-05	4	416	4	23090	GSMUA_Achr3G09960_001,GSMUA_Achr6G05000_001,GSMUA_Achr3G15320_001,GSMUA_Achr9G03350_001
GO:00462	lignin catal	biological_	1.04E-07	1.30E-05	4	416	4	23090	GSMUA_Achr3G09960_001,GSMUA_Achr9G03350_001,GSMUA_Achr6G05000_001,GSMUA_Achr3G15320_001
GO:00527	hydroquinc	molecular_	1.04E-07	1.30E-05	4	416	4	23090	GSMUA_Achr3G09960_001,GSMUA_Achr3G15320_001,GSMUA_Achr6G05000_001,GSMUA_Achr9G03350_001
GO:00507	regulation	biological_	1.39E-07	1.70E-05	100	416	3401	23090	GSMUA_Achr7G00440_001,GSMUA_Achr11G25720_001,GSMUA_Achr11G23870_001,GSMUA_Achr9G29480_001,GSMUA_AchrUn_randomG04680_001, [illegible]
GO:00442	cellular nit	biological_	1.59E-07	1.91E-05	98	416	3320	23090	GSMUA_Achr6G36740_001,GSMUA_Achr6G30050_001,GSMUA_AchrUn_randomG16250_001,GSMUA_Achr6G34420_001,GSMUA_Achr5G26580_001, [illegible]
GO:00061	nucleobase	biological_	1.78E-07	2.04E-05	106	416	3695	23090	GSMUA_Achr9G26140_001,GSMUA_Achr6G04510_001,GSMUA_Achr3G29730_001,GSMUA_Achr6G30570_001,GSMUA_Achr6G18900_001,GSMUA_Achr5G29290_001, [illegible]
GO:00713	cellular res	biological_	1.79E-07	2.04E-05	19	416	252	23090	GSMUA_Achr9G29480_001,GSMUA_Achr4G18240_001,GSMUA_Achr5G03960_001,GSMUA_Achr5G18540_001,GSMUA_Achr10G04600_001,GSMUA_Achr5G14630_001, [illegible]
GO:00507	regulation	biological_	1.80E-07	2.04E-05	92	416	3058	23090	GSMUA_Achr5G09480_001,GSMUA_AchrUn_randomG12610_001,GSMUA_Achr9G04270_001,GSMUA_Achr4G11090_001,GSMUA_Achr9G03060_001, [illegible]
GO:19013	organic cy	biological_	1.99E-07	2.20E-05	113	416	4030	23090	GSMUA_Achr10G22610_001,GSMUA_Achr5G18540_001,GSMUA_Achr4G06100_001,GSMUA_Achr7G20550_001,GSMUA_Achr5G06550_001,GSMUA_Achr5G20860_001, [illegible]
GO:00650	biological	biological_	2.32E-07	2.53E-05	102	416	3531	23090	GSMUA_Achr3G08080_001,GSMUA_Achr4G07190_001,GSMUA_Achr8G33310_001,GSMUA_Achr3G23290_001,GSMUA_Achr5G26490_001,GSMUA_Achr5G14630_001, [illegible]
GO:00708	cellular res	biological_	4.12E-07	4.39E-05	19	416	266	23090	GSMUA_Achr4G25960_001,GSMUA_Achr5G26580_001,GSMUA_Achr11G18520_001,GSMUA_Achr6G19850_001,GSMUA_Achr8G18930_001,GSMUA_Achr5G02450_001, [illegible]
GO:19013	heterocycl	molecular_	4.34E-07	4.51E-05	189	416	7895	23090	GSMUA_Achr9G22260_001,GSMUA_Achr6G22280_001,GSMUA_Achr8G30190_001,GSMUA_Achr10G26180_001,GSMUA_Achr6G06030_001,GSMUA_Achr5G00590_001, [illegible]
GO:00971	organic cy	molecular_	4.38E-07	4.51E-05	189	416	7896	23090	GSMUA_Achr6G02530_001,GSMUA_Achr8G33560_001,GSMUA_Achr11G17950_001,GSMUA_Achr9G20620_001,GSMUA_Achr2G06420_001, [illegible]
GO:00464	heterocycl	biological_	4.73E-07	4.79E-05	109	416	3913	23090	GSMUA_Achr9G06850_001,GSMUA_Achr11G18520_001,GSMUA_Achr7G03320_001,GSMUA_Achr4G25960_001,GSMUA_Achr2G13090_001,GSMUA_Achr3G29730_001, [illegible]
GO:00056	cell	cellular_co	1.44E-06	0.00014	156	416	6271	23090	GSMUA_Achr6G22280_001,GSMUA_Achr9G22260_001,GSMUA_Achr6G06030_001,GSMUA_Achr10G26180_001,GSMUA_Achr5G00590_001,GSMUA_Achr5G18530_001, [illegible]
GO:00444	cell part	cellular_co	1.44E-06	0.00014	156	416	6271	23090	GSMUA_Achr8G18550_001,GSMUA_Achr1G15010_001,GSMUA_Achr8G12560_001,GSMUA_Achr10G12980_001,GSMUA_Achr5G00500_001,GSMUA_Achr8G09300_001, [illegible]
GO:00098	lignin meta	biological_	1.51E-06	0.000145	4	416	6	23090	GSMUA_Achr3G09960_001,GSMUA_Achr3G15320_001,GSMUA_Achr6G05000_001,GSMUA_Achr9G03350_001
GO:00448	macromole	molecular_	2.24E-06	0.000212	33	416	746	23090	GSMUA_Achr7G18550_001,GSMUA_Achr9G20620_001,GSMUA_Achr11G23500_001,GSMUA_Achr8G24960_001,GSMUA_Achr6G36930_001,GSMUA_Achr9G15920_001, [illegible]
GO:00036	chromatin	molecular_	3.08E-06	0.000286	28	416	585	23090	GSMUA_Achr8G18460_001,GSMUA_Achr1G18610_001,GSMUA_Achr3G27700_001,GSMUA_Achr8G17380_001,GSMUA_Achr8G31350_001,GSMUA_Achr11G22820_001, [illegible]
GO:00104	gene expre	biological_	6.62E-06	0.000605	95	416	3417	23090	GSMUA_Achr10G26180_001,GSMUA_Achr1G05940_001,GSMUA_Achr5G00590_001,GSMUA_Achr11G04360_001,GSMUA_Achr11G25770_001,GSMUA_Achr10G22350_001, [illegible]
GO:00054	binding	molecular_	7.03E-06	0.000633	299	416	14278	23090	GSMUA_Achr4G12860_001,GSMUA_Achr1G23170_001,GSMUA_Achr8G18930_001,GSMUA_Achr5G02450_001,GSMUA_Achr6G05690_001,GSMUA_Achr9G20220_001, [illegible]
GO:00100	response to	biological_	9.09E-06	0.000806	21	416	389	23090	GSMUA_Achr6G19850_001,GSMUA_Achr11G18520_001,GSMUA_Achr5G26580_001,GSMUA_Achr4G25960_001,GSMUA_Achr10G26310_001,GSMUA_Achr11G01610_001, [illegible]
GO:00346	cellular ma	biological_	1.88E-05	0.001642	93	416	3406	23090	GSMUA_Achr5G26640_001,GSMUA_Achr3G29970_001,GSMUA_Achr5G07270_001,GSMUA_Achr11G13590_001,GSMUA_Achr4G18240_001,GSMUA_Achr3G23360_001, [illegible]
GO:00047	sulfate ade	molecular_	2.29E-05	0.001971	3	416	4	23090	GSMUA_Achr1G14080_001,GSMUA_Achr3G13570_001,GSMUA_Achr8G26890_001
GO:00090	macromole	biological_	3.68E-05	0.003116	93	416	3464	23090	GSMUA_Achr5G26640_001,GSMUA_Achr3G29970_001,GSMUA_Achr3G23360_001,GSMUA_Achr5G07270_001,GSMUA_Achr11G13590_001,GSMUA_Achr4G18240_001, [illegible]
GO:00047	sulfate ade	molecular_	5.65E-05	0.004721	3	416	5	23090	GSMUA_Achr1G14080_001,GSMUA_Achr3G13570_001,GSMUA_Achr8G26890_001
GO:00346	cellular nit	biological_	0.000201	0.016325	112	416	4553	23090	GSMUA_Achr4G18240_001,GSMUA_Achr5G07270_001,GSMUA_Achr11G13590_001,GSMUA_Achr3G23360_001,GSMUA_Achr3G29970_001,GSMUA_Achr3G19800_001, [illegible]
GO:00422	response to	biological_	0.000201	0.016325	23	416	554	23090	GSMUA_Achr9G29480_001,GSMUA_Achr5G03960_001,GSMUA_Achr4G18240_001,GSMUA_Achr5G18540_001,GSMUA_Achr10G04600_001,GSMUA_Achr11G13300_001, [illegible]
GO:00001	sulfate assi	biological_	0.000304	0.024334	3	416	8	23090	GSMUA_Achr1G14080_001,GSMUA_Achr8G26890_001,GSMUA_Achr3G13570_001
GO:00166	oxidoreduc	molecular_	0.000325	0.0256	4	416	19	23090	GSMUA_Achr9G03350_001,GSMUA_Achr3G15320_001,GSMUA_Achr6G05000_001,GSMUA_Achr3G09960_001
GO:00055	cellular_co	cellular_co	0.000329	0.0256	198	416	9080	23090	GSMUA_Achr8G01450_001,GSMUA_Achr7G03320_001,GSMUA_Achr7G09580_001,GSMUA_Achr5G16960_001,GSMUA_Achr11G18520_001,GSMUA_Achr8G24810_001, [illegible]
GO:00442	cellular bio	biological_	0.000464	0.035647	109	416	4501	23090	GSMUA_Achr3G23360_001,GSMUA_Achr11G13590_001,GSMUA_Achr5G07270_001,GSMUA_Achr4G18240_001,GSMUA_Achr5G26640_001,GSMUA_Achr3G29970_001, [illegible]
GO:00068	nitrogen co	biological_	0.000804	0.061004	116	416	4920	23090	GSMUA_Achr7G20550_001,GSMUA_Achr5G20860_001,GSMUA_Achr5G06550_001,GSMUA_Achr5G01490_001,GSMUA_Achr10G22610_001,GSMUA_Achr5G18540_001, [illegible]
GO:00096	phenylprop	biological_	0.000973	0.072856	4	416	25	23090	GSMUA_Achr3G09960_001,GSMUA_Achr9G03350_001,GSMUA_Achr6G05000_001,GSMUA_Achr3G15320_001
GO:19015	organic sul	biological_	0.00161	0.11897	108	416	4608	23090	GSMUA_Achr5G14630_001,GSMUA_Achr5G26490_001,GSMUA_Achr3G23290_001,GSMUA_Achr8G33310_001,GSMUA_Achr4G07190_001,GSMUA_Achr11G20390_001, [illegible]
GO:00090	biosyntheti	biological_	0.00172	0.12548	112	416	4819	23090	GSMUA_Achr10G24770_001,GSMUA_Achr6G31350_001,GSMUA_Achr5G17720_001,GSMUA_Achr8G11160_001,GSMUA_Achr6G29460_001,GSMUA_Achr1G20690_001, [illegible]
GO:00047	shikimate 3	molecular_	0.001897	0.13497	2	416	4	23090	GSMUA_Achr7G03670_001,GSMUA_Achr7G03660_001
GO:00196	shikimate r	biological_	0.001897	0.13497	2	416	4	23090	GSMUA_Achr7G03670_001,GSMUA_Achr7G03660_001
GO:00166	oxidoreduc	molecular_	0.001965	0.13806	4	416	30	23090	GSMUA_Achr3G09960_001,GSMUA_Achr6G05000_001,GSMUA_Achr3G15320_001,GSMUA_Achr9G03350_001
GO:00038	3-dehydroc	molecular_	0.003124	0.21691	2	416	5	23090	GSMUA_Achr7G03670_001,GSMUA_Achr7G03660_001
GO:00071	signal trans	biological_	0.003259	0.22356	25	416	765	23090	GSMUA_Achr5G14630_001,GSMUA_Achr3G23290_001,GSMUA_AchrUn_randomG06470_001,GSMUA_Achr8G28560_001,GSMUA_Achr5G18540_001, [illegible]
GO:00447	single orga	biological_	0.00391	0.26508	25	416	776	23090	GSMUA_Achr4G18240_001,GSMUA_Achr5G03960_001,GSMUA_Achr9G29480_001,GSMUA_AchrUn_randomG02030_001,GSMUA_Achr10G04600_001, [illegible]
GO:00230	signaling	biological_	0.003974	0.26631	25	416	777	23090	GSMUA_Achr9G29480_001,GSMUA_AchrUn_randomG02030_001,GSMUA_Achr4G18240_001,GSMUA_Achr5G03960_001,GSMUA_Achr5G14630_001, [illegible]
GO:00705	adenylyltra	molecular_	0.00454	0.30076	3	416	19	23090	GSMUA_Achr1G14080_001,GSMUA_Achr8G26890_001,GSMUA_Achr3G13570_001
GO:00068	retrograde	biological_	0.00463	0.30323	2	416	6	23090	GSMUA_Achr1G20180_001,GSMUA_Achr4G04300_001
GO:00071	cell comm	biological_	0.005846	0.37856	26	416	844	23090	GSMUA_Achr11G25770_001,GSMUA_Achr8G13620_001,GSMUA_Achr8G18930_001,GSMUA_Achr5G02450_001,GSMUA_Achr5G00590_001,GSMUA_Achr9G28750_001, [illegible]
GO:00100	primary sh	biological_	0.006405	0.40565	2	416	7	23090	GSMUA_Achr5G18530_001,GSMUA_Achr5G01490_001
GO:00904	embryonic	biological_	0.006405	0.40565	2	416	7	23090	GSMUA_Achr5G18530_001,GSMUA_Achr5G01490_001
GO:00039	adenine ph	molecular_	0.008312	0.49896	2	416	8	23090	GSMUA_Achr8G18490_001,GSMUA_Achr3G19800_001
GO:00061	adenine sal	biological_	0.008312	0.49896	2	416	8	23090	GSMUA_Achr3G19800_001,GSMUA_Achr8G18490_001
GO:00430	purine nucl	biological_	0.008312	0.49896	2	416	8	23090	GSMUA_Achr3G19800_001,GSMUA_Achr8G18490_001
GO:00460	adenine m	biological_	0.008312	0.49896	2	416	8	23090	GSMUA_Achr8G18490_001,GSMUA_Achr3G19800_001
GO:00460	adenine bi	biological_	0.008312	0.49896	2	416	8	23090	GSMUA_Achr8G18490_001,GSMUA_Achr3G19800_001
GO:00086	protein ph	molecular_	0.008874	0.52722	3	416	24	23090	GSMUA_AchrUn_randomG02030_001,GSMUA_Achr7G09580_001,GSMUA_Achr9G28750_001
GO:00517	cellular res	biological_	0.009604	0.56097	31	416	1100	23090	GSMUA_Achr5G14630_001,GSMUA_Achr3G23290_001,GSMUA_AchrUn_randomG06470_001,GSMUA_Achr8G28560_001,GSMUA_Achr8G12560_001, [illegible]
GO:00469	protein din	molecular_	0.009637	0.56097	22	416	707	23090	GSMUA_Achr5G00590_001,GSMUA_Achr8G11070_001,GSMUA_Achr9G09260_001,GSMUA_Achr10G26310_001,GSMUA_Achr11G01610_001,GSMUA_Achr8G13620_001, [illegible]
GO:00001	protein ph	cellular_co	0.009951	0.56225	3	416	25	23090	GSMUA_AchrUn_randomG02030_001,GSMUA_Achr9G28750_001,GSMUA_Achr7G09580_001
GO:00082	protein ser	cellular_co	0.009951	0.56225	3	416	25	23090	GSMUA_AchrUn_randomG02030_001,GSMUA_Achr7G09580_001,GSMUA_Achr9G28750_001
GO:19032	phosphatas	cellular_co	0.009951	0.56225	3	416	25	23090	GSMUA_Achr9G28750_001,GSMUA_Achr7G09580_001,GSMUA_AchrUn_randomG02030_001
GO:00455	regulation	biological_	0.010722	0.59413	2	416	9	23090	GSMUA_Achr5G01490_001,GSMUA_Achr5G18530_001
GO:00485	embryonic	biological_	0.010722	0.59413	2	416	9	23090	GSMUA_Achr5G01490_001,GSMUA_Achr5G18530_001
GO:00198	protein ph	molecular_	0.011101	0.6093	3	416	26	23090	GSMUA_Achr9G28750_001,GSMUA_Achr7G09580_001,GSMUA_AchrUn_randomG02030_001
GO:19013	organic cy	biological_	0.01413	0.76822	6	416	109	23090	GSMUA_Achr3G15320_001,GSMUA_Achr6G05000_001,GSMUA_Achr9G03350_001,GSMUA_Achr8G22970_001,GSMUA_Achr8G09010_001,GSMUA_Achr3G09960_001
GO:00192	phosphatas	molecular_	0.014995	0.80764	3	416	29	23090	GSMUA_Achr9G28750_001,GSMUA_Achr7G09580_001,GSMUA_AchrUn_randomG02030_001
GO:00100	shoot syste	biological_	0.015915	0.84564	6	416	112	23090	GSMUA_Achr5G01490_001,GSMUA_Achr6G28900_001,GSMUA_Achr7G13570_001,GSMUA_Achr8G28560_001,GSMUA_Achr5G18530_001,GSMUA_Achr3G24160_001
GO:00300	L-malate d	molecular_	0.015994	0.84564	2	416	11	23090	GSMUA_Achr5G02000_001,GSMUA_AchrUn_randomG11210_001
GO:00091	nucleobase	biological_	0.016352	0.85671	3	416	30	23090	GSMUA_Achr8G22970_001,GSMUA_Achr3G19800_001,GSMUA_Achr8G18490_001
GO:00165	superoxide	molecular_	0.017999	0.86529	1	416	1	23090	GSMUA_Achr4G24900_001
GO:00193	siroheme b	biological_	0.018017	0.86529	1	416	1	23090	GSMUA_Achr6G27140_001
GO:00431	precorrin-2	molecular_	0.018017	0.86529	1	416	1	23090	GSMUA_Achr6G27140_001
GO:00461	siroheme m	biological_	0.018017	0.86529	1	416	1	23090	GSMUA_Achr6G27140_001
GO:00171	dihydropyr	molecular_	0.018017	0.86529	1	416	1	23090	GSMUA_Achr8G22970_001
GO:00191	beta-1,4-m	molecular_	0.018017	0.86529	1	416	1	23090	GSMUA_Achr3G24160_001
GO:00315	wybutosin	biological_	0.018017	0.86529	1	416	1	23090	GSMUA_Achr6G29460_001
GO:00315	wybutosin	biological_	0.018017	0.86529	1	416	1	23090	GSMUA_Achr6G29460_001
GO:00472	galactinol-	molecular_	0.018017	0.86529	1	416	1	23090	GSMUA_Achr11G14030_001
GO:00517	mannan sy	molecular_	0.018017	0.86529	1	416	1	23090	GSMUA_Achr3G24160_001
GO:00082	nucleotidas	molecular_	0.018962	0.89601	2	416	12	23090	GSMUA_Achr8G03300_001,GSMUA_Achr3G25870_001
GO:00339	glycopeptid	molecular_	0.018968	0.89601	2	416	12	23090	GSMUA_Achr3G19090_001,GSMUA_Achr11G16520_001
GO:00082	metalloexo	molecular_	0.022153	1	2	416	13	23090	GSMUA_Achr8G24810_001,GSMUA_Achr7G19370_001
GO:00091	purine nucl	biological_	0.025328	1	2	416	14	23090	GSMUA_Achr8G18490_001,GSMUA_Achr3G19800_001
GO:00431	purine-con	biological_	0.025328	1	2	416	14	23090	GSMUA_Achr3G19800_001,GSMUA_Achr8G18490_001
GO:00041	cytochrom	molecular_	0.025468	1	2	416	14	23090	GSMUA_Achr5G08780_001,GSMUA_Achr10G27090_001
GO:00150	heme-copp	molecular_	0.025468	1	2	416	14	23090	GSMUA_Achr10G27090_001,GSMUA_Achr5G08780_001
GO:00166	oxidoreduc	molecular_	0.025468	1	2	416	14	23090	GSMUA_Achr10G27090_001,GSMUA_Achr5G08780_001
GO:00166	oxidoreduc	molecular_	0.025468	1	2	416	14	23090	GSMUA_Achr10G27090_001,GSMUA_Achr5G08780_001
GO:00061	purine nucl	biological_	0.032658	1	2	416	16	23090	GSMUA_Achr8G18490_001,GSMUA_Achr3G19800_001
GO:00725	reactive ox	biological_	0.032718	1	4	416	67	23090	GSMUA_Achr6G05690_001,GSMUA_Achr2G02380_001,GSMUA_Achr4G24900_001,GSMUA_Achr5G01490_001
GO:00053	organic aci	molecular_	0.035096	1	3	416	40	23090	GSMUA_Achr6G19840_001,GSMUA_Achr9G24350_001,GSMUA_Achr7G07960_001
GO:00469	carboxylic	molecular_	0.035096	1	3	416	40	23090	GSMUA_Achr6G19840_001,GSMUA_Achr9G24350_001,GSMUA_Achr7G07960_001
GO:00311	SCF-depen	biological_	0.035668	1	1	416	2	23090	GSMUA_Achr3G23630_001
GO:00165	copper cha	molecular_	0.035693	1	1	416	2	23090	GSMUA_Achr4G24900_001
GO:00081	poly-pyrim	molecular_	0.035697	1	1	416	2	23090	GSMUA_Achr8G02340_001
GO:00082	poly(U) R	molecular_	0.035697	1	1	416	2	23090	GSMUA_Achr8G02340_001
GO:00041	dihydrooro	molecular_	0.035711	1	1	416	2	23090	GSMUA_Achr8G22970_001
GO:00057	signal reco	cellular_co	0.035711	1	1	416	2	23090	GSMUA_Achr11G21230_001
GO:00062	pyrimidine	biological_	0.035711	1	1	416	2	23090	GSMUA_Achr8G22970_001
GO:00062	uracil catal	biological_	0.035711	1	1	416	2	23090	GSMUA_Achr8G22970_001
GO:00086	2-C-methy	molecular_	0.035711	1	1	416	2	23090	GSMUA_Achr5G03350_001
GO:00160	zeta DNA p	cellular_co	0.035711	1	1	416	2	23090	GSMUA_Achr11G20390_001
GO:00166	oxidoreduc	molecular_	0.035711	1	1	416	2	23090	GSMUA_Achr1G22900_001
GO:00168	phosphorus	molecular_	0.035711	1	1	416	2	23090	GSMUA_Achr5G03350_001
GO:00461	nucleobase	biological_	0.035711	1	1	416	2	23090	GSMUA_Achr8G22970_001
GO:00503	sulfite redu	molecular_	0.035711	1	1	416	2	23090	GSMUA_Achr1G22900_001
GO:00800	photosyste	biological_	0.035711	1	1	416	2	23090	GSMUA_Achr8G28560_001
GO:00194	aromatic c	biological_	0.036042	1	5	416	101	23090	GSMUA_Achr3G15320_001,GSMUA_Achr6G05000_001,GSMUA_Achr9G03350_001,GSMUA_Achr8G09010_001,GSMUA_Achr3G09960_001
GO:00068	superoxide	biological_	0.040897	1	2	416	18	23090	GSMUA_Achr2G02380_001,GSMUA_Achr4G24900_001
GO:00041	aminopeptid	molecular_	0.040966	1	2	416	18	23090	GSMUA_Achr8G24810_001,GSMUA_Achr7G19370_001
GO:00010	nucleic aci	molecular_	0.042023	1	27	416	1052	23090	GSMUA_Achr8G18550_001,GSMUA_Achr8G20090_001,GSMUA_Achr4G06100_001,GSMUA_Achr2G21430_001,GSMUA_Achr10G25270_001,GSMUA_Achr10G03230_001, [illegible]
GO:00037	transcriptio	molecular_	0.042023	1	27	416	1052	23090	GSMUA_Achr10G03230_001,GSMUA_Achr1G26740_001,GSMUA_Achr5G06550_001,GSMUA_Achr8G18550_001,GSMUA_Achr10G25270_001,GSMUA_Achr8G20090_001, [illegible]
[illegible]	[illegible]	[illegible]	[illegible]	[illegible]	[illegible]	[illegible]	[illegible]	[illegible]	[illegible]

GO_accession	Description	Term_type	Over_represented_pValue	Corrected_pValue	DEG_item	DEG_list	Bg_item	Bg_list	Gene_IDs
GO:00056	nucleus	cellular_component	9.97E-25	5.74E-21	108	402	2159	23090	GSMUA_Achr4G12860_001,GSMUA_Achr8G12560_001,GSMUA_Achr1G09830_001,GSMUA_Achr7G03320_001,GSMUA_Achr8G18550_001,GSMUA_Achr2G18380_001,[illegible]
GO:00055	copper ion	molecular_function	7.95E-18	2.29E-14	26	402	163	23090	GSMUA_Achr6G33870_001,GSMUA_Achr9G17900_001,GSMUA_Achr9G03350_001,GSMUA_Achr8G23110_001,GSMUA_Achr5G04350_001,[illegible]
GO:00432	membrane-	cellular_component	4.55E-17	6.56E-14	132	402	3730	23090	[illegible]
GO:00432	intracellul	cellular_component	4.55E-17	6.56E-14	132	402	3730	23090	[illegible]
GO:00036	DNA bindi	molecular_function	2.32E-16	2.67E-13	104	402	2629	23090	[illegible]
GO:00097	auxin-activ	biological_process	5.15E-14	4.24E-11	19	402	110	23090	[illegible]
GO:00713	cellular res	biological_process	5.15E-14	4.24E-11	19	402	110	23090	[illegible]
GO:00166	oxidoreduc	molecular_function	1.81E-13	1.31E-10	10	402	19	23090	[illegible]
GO:00097	response to	biological_process	9.98E-13	6.39E-10	19	402	129	23090	[illegible]
GO:00346	nucleobase	biological_process	1.15E-12	6.65E-10	91	402	2455	23090	[illegible]
GO:00432	intracellul	cellular_component	3.37E-12	1.44E-09	138	402	4597	23090	[illegible]
GO:00432	organelle	cellular_component	3.43E-12	1.44E-09	138	402	4598	23090	[illegible]
GO:00181	heterocycl	biological_process	3.50E-12	1.44E-09	94	402	2630	23090	[illegible]
GO:00194	aromatic	biological_process	3.50E-12	1.44E-09	95	402	2672	23090	[illegible]
GO:00063	transcripti	biological_process	6.36E-12	2.29E-09	83	402	2204	23090	[illegible]
GO:00976	nucleic aci	biological_process	6.36E-12	2.29E-09	83	402	2204	23090	[illegible]
GO:00327	RNA biosy	biological_process	6.82E-12	2.31E-09	83	402	2207	23090	[illegible]
[illegible]									

Supplemental data S7 KEGG enrichment result of the predicted target genes of differentially expressed (DE) miRNAs

Term	Database	ID	Input numl	Backgroun	P-Value	Corrected	Gene IDs
Peroxisome	KEGG PATHWAY	mus04146	6	97	0.003479	0.21567	GSMUA_Achr4G07060_001|GSMUA_Achr4G09570_001|GSMUA_Achr11G18410_001|GSMUA_Achr2G02380_001|GSMUA_Achr8G30270_001|GSMUA_Achr1G13190_001
Fatty acid biosynthesis	KEGG PATHWAY	mus00061	4	54	0.009221	0.28584	GSMUA_Achr8G24640_001|GSMUA_Achr10G19000_001|GSMUA_Achr1G13190_001|GSMUA_Achr8G30270_001
SNARE interactions in vesicular transport	KEGG PATHWAY	mus04130	3	52	0.043225	0.651163	GSMUA_Achr8G03020_001|GSMUA_Achr10G28310_001|GSMUA_Achr6G31140_001
Nicotinate and nicotinamide metabolism	KEGG PATHWAY	mus00760	2	23	0.048651	0.651163	GSMUA_Achr3G25870_001|GSMUA_Achr8G03300_001
Fatty acid metabolism	KEGG PATHWAY	mus01212	4	95	0.052513	0.651163	GSMUA_Achr8G24640_001|GSMUA_Achr10G19000_001|GSMUA_Achr1G13190_001|GSMUA_Achr8G30270_001
Propanoate metabolism	KEGG PATHWAY	mus00640	2	33	0.088083	0.710235	GSMUA_Achr8G24640_001|GSMUA_Achr10G19000_001
Synthesis and degradation of ketone bodies	KEGG PATHWAY	mus00072	1	7	0.108202	0.710235	GSMUA_Achr9G00950_001
Cysteine and methionine metabolism	KEGG PATHWAY	mus00270	4	128	0.118833	0.710235	GSMUA_Achr3G16330_001|GSMUA_Achr9G18460_001|GSMUA_Achr4G28160_001|GSMUA_AchrUn_randomG11210_001
Sulfur metabolism	KEGG PATHWAY	mus00920	2	44	0.138782	0.710235	GSMUA_Achr3G16330_001|GSMUA_Achr1G22900_001
Fatty acid degradation	KEGG PATHWAY	mus00071	2	45	0.143662	0.710235	GSMUA_Achr1G13190_001|GSMUA_Achr8G30270_001
Fructose and mannose metabolism	KEGG PATHWAY	mus00051	3	90	0.146142	0.710235	GSMUA_Achr7G05110_001|GSMUA_Achr11G18240_001|GSMUA_Achr4G20510_001
Glycine, serine and threonine metabolism	KEGG PATHWAY	mus00260	3	92	0.152843	0.710235	GSMUA_Achr4G28160_001|GSMUA_Achr4G09570_001|GSMUA_Achr3G23560_001
Pyrimidine metabolism	KEGG PATHWAY	mus00240	4	142	0.154346	0.710235	GSMUA_Achr3G25870_001|GSMUA_Achr9G23310_001|GSMUA_Achr8G03300_001|GSMUA_Achr9G01450_001
Metabolic pathways	KEGG PATHWAY	mus01100	39	2344	0.186574	0.710235	GSMUA_AchrUn_randomG11210_001|GSMUA_Achr3G04930_001|GSMUA_Achr7G03670_001|GSMUA_Achr8G26330_001|GSMUA_Achr10G27090_001|GSMUA_Achr1G22400_001|GSMUA_Achr2G17330_001|GSMUA_Achr4G09570_001|GSMUA_Achr4G20510_001|GSMUA_Achr1G22900_001|GSMUA_Achr8G24640_001|GSMUA_Achr7G03660_001|GSMUA_Achr7G05110_001|GSMUA_Achr3G25870_001|GSMUA_Achr9G00950_001|GSMUA_Achr4G16070_001|GSMUA_Achr3G23340_001|GSMUA_Achr9G18460_001|GSMUA_Achr10G23010_001|GSMUA_Achr5G07520_001|GSMUA_Achr8G29090_001|GSMUA_Achr4G28160_001|GSMUA_Achr8G03300_001|GSMUA_Achr4G32620_001|GSMUA_Achr11G18240_001|GSMUA_Achr3G16330_001|GSMUA_Achr8G08790_001|GSMUA_AchrUn_randomG26070_001|GSMUA_Achr5G20960_001|GSMUA_AchrUn_randomG26330_001|GSMUA_Achr2G05090_001|GSMUA_Achr2G04860_001|GSMUA_Achr9G23310_001|GSMUA_Achr8G30270_001|GSMUA_Achr6G35540_001|[illegible]
Pyruvate metabolism	KEGG PATHWAY	mus00620	3	108	0.209502	0.710235	GSMUA_AchrUn_randomG11210_001|GSMUA_Achr8G24640_001|GSMUA_Achr10G19000_001
Terpenoid backbone biosynthesis	KEGG PATHWAY	mus00900	2	63	0.235863	0.710235	GSMUA_Achr5G03350_001|GSMUA_Achr9G00950_001
mRNA surveillance pathway	KEGG PATHWAY	mus03015	4	173	0.244152	0.710235	GSMUA_AchrUn_randomG02030_001|GSMUA_Achr3G26010_001|GSMUA_Achr8G01190_001|GSMUA_Achr8G06590_001
Phenylalanine, tyrosine and tryptophan bios	KEGG PATHWAY	mus00400	2	65	0.246394	0.710235	GSMUA_Achr7G03660_001|GSMUA_Achr7G03670_001
Lysine biosynthesis	KEGG PATHWAY	mus00300	1	19	0.249052	0.710235	GSMUA_Achr4G28160_001
Protein export	KEGG PATHWAY	mus03060	2	67	0.256942	0.710235	GSMUA_Achr5G20650_001|GSMUA_Achr11G21230_001
Brassinosteroid biosynthesis	KEGG PATHWAY	mus00905	1	20	0.259738	0.710235	GSMUA_Achr8G26330_001
Inositol phosphate metabolism	KEGG PATHWAY	mus00562	2	70	0.272776	0.710235	GSMUA_Achr10G23010_001|GSMUA_Achr1G22400_001
Butanoate metabolism	KEGG PATHWAY	mus00650	1	22	0.280659	0.710235	GSMUA_Achr9G00950_001
Glycosaminoglycan degradation	KEGG PATHWAY	mus00531	1	23	0.290897	0.710235	GSMUA_Achr4G32620_001
ABC transporters	KEGG PATHWAY	mus02010	1	24	0.300991	0.710235	GSMUA_Achr4G12130_001
Purine metabolism	KEGG PATHWAY	mus00230	4	193	0.307182	0.710235	GSMUA_Achr3G25870_001|GSMUA_Achr9G23310_001|GSMUA_Achr8G03300_001|GSMUA_Achr9G01450_001
Glyoxylate and dicarboxylate metabolism	KEGG PATHWAY	mus00630	2	77	0.309631	0.710235	GSMUA_AchrUn_randomG11210_001|GSMUA_Achr3G23560_001
Cutin, suberine and wax biosynthesis	KEGG PATHWAY	mus00073	1	26	0.320751	0.710235	GSMUA_Achr9G08080_001
Phosphatidylinositol signaling system	KEGG PATHWAY	mus04070	2	84	0.346105	0.739948	GSMUA_Achr10G23010_001|GSMUA_Achr1G22400_001
Starch and sucrose metabolism	KEGG PATHWAY	mus00500	5	276	0.369665	0.747172	GSMUA_Achr4G16070_001|GSMUA_Achr3G04930_001|GSMUA_Achr8G08790_001|GSMUA_Achr3G23340_001|GSMUA_Achr6G35540_001
RNA transport	KEGG PATHWAY	mus03013	4	214	0.374908	0.747172	GSMUA_Achr9G13950_001|GSMUA_Achr3G26010_001|GSMUA_Achr6G11580_001|GSMUA_Achr1G28070_001
Lysine degradation	KEGG PATHWAY	mus00310	1	33	0.385637	0.747172	GSMUA_Achr4G09570_001
Phenylpropanoid biosynthesis	KEGG PATHWAY	mus00940	4	231	0.429445	0.795888	GSMUA_Achr2G05090_001|GSMUA_AchrUn_randomG26070_001|GSMUA_Achr2G04860_001|GSMUA_AchrUn_randomG26330_001
Carbon fixation in photosynthetic organism	KEGG PATHWAY	mus00710	2	102	0.436455	0.795888	GSMUA_AchrUn_randomG11210_001|GSMUA_Achr11G18240_001
RNA polymerase	KEGG PATHWAY	mus03020	1	43	0.467777	0.805616	GSMUA_Achr9G23310_001
Tyrosine metabolism	KEGG PATHWAY	mus00350	1	43	0.467777	0.805616	GSMUA_Achr8G29090_001
Valine, leucine and isoleucine degradation	KEGG PATHWAY	mus00280	1	46	0.490217	0.806841	GSMUA_Achr9G00950_001
Oxidative phosphorylation	KEGG PATHWAY	mus00190	3	183	0.494516	0.806841	GSMUA_Achr3G13280_001|GSMUA_Achr1G26020_001|GSMUA_Achr10G27090_001
Amino sugar and nucleotide sugar metaboli	KEGG PATHWAY	mus00520	3	188	0.512254	0.814353	GSMUA_Achr7G05110_001|GSMUA_Achr8G08790_001|GSMUA_Achr4G20510_001
N-Glycan biosynthesis	KEGG PATHWAY	mus00510	1	51	0.525546	0.814596	GSMUA_Achr5G07520_001
Basal transcription factors	KEGG PATHWAY	mus03022	1	58	0.57095	0.860313	GSMUA_AchrUn_randomG25670_001
RNA degradation	KEGG PATHWAY	mus03018	2	145	0.62103	0.860313	GSMUA_Achr6G31430_001|GSMUA_Achr5G04250_001
Citrate cycle (TCA cycle)	KEGG PATHWAY	mus00020	1	68	0.628414	0.860313	GSMUA_AchrUn_randomG11210_001
Plant hormone signal transduction	KEGG PATHWAY	mus04075	6	453	0.639542	0.860313	GSMUA_Achr2G06770_001|GSMUA_Achr7G09190_001|GSMUA_Achr10G10440_001|GSMUA_Achr5G26930_001|GSMUA_Achr3G23850_001|GSMUA_Achr10G26310_001
Photosynthesis	KEGG PATHWAY	mus00195	1	73	0.654207	0.860313	GSMUA_Achr2G17330_001
Proteasome	KEGG PATHWAY	mus03050	1	73	0.654207	0.860313	GSMUA_Achr4G00300_001
Pentose phosphate pathway	KEGG PATHWAY	mus00030	1	74	0.659147	0.860313	GSMUA_Achr11G18240_001
Carbon metabolism	KEGG PATHWAY	mus01200	4	321	0.682289	0.860313	GSMUA_Achr3G16330_001|GSMUA_AchrUn_randomG11210_001|GSMUA_Achr8G24640_001|GSMUA_Achr11G18240_001
Galactose metabolism	KEGG PATHWAY	mus00052	1	81	0.69182	0.860313	GSMUA_Achr3G23340_001
Biosynthesis of amino acids	KEGG PATHWAY	mus01230	4	326	0.6938	0.860313	GSMUA_Achr3G16330_001|GSMUA_Achr7G03660_001|GSMUA_Achr7G03670_001|GSMUA_Achr4G28160_001
Biosynthesis of secondary metabolites	KEGG PATHWAY	mus01110	16	1235	0.708165	0.860906	GSMUA_AchrUn_randomG11210_001|GSMUA_Achr7G03660_001|GSMUA_Achr7G03670_001|GSMUA_Achr8G26330_001|GSMUA_Achr8G24640_001|GSMUA_AchrUn_randomG26070_001|GSMUA_Achr3G23560_001|GSMUA_Achr2G05090_001|GSMUA_Achr3G25870_001|GSMUA_Achr2G04860_001|GSMUA_Achr4G28160_001|GSMUA_Achr8G03300_001|GSMUA_Achr9G00950_001|GSMUA_Achr5G03350_001|GSMUA_Achr11G18240_001|GSMUA_AchrUn_randomG26330_001
Glycerolipid metabolism	KEGG PATHWAY	mus00561	1	90	0.72929	0.868029	GSMUA_Achr5G20960_001
Arginine and proline metabolism	KEGG PATHWAY	mus00330	1	94	0.744454	0.868029	GSMUA_Achr9G18460_001
Glutathione metabolism	KEGG PATHWAY	mus00480	1	102	0.772293	0.868029	GSMUA_Achr9G01450_001
Pentose and glucuronate interconversions	KEGG PATHWAY	mus00040	1	105	0.781936	0.868029	GSMUA_Achr6G35540_001
Ribosome	KEGG PATHWAY	mus03010	5	453	0.784026	0.868029	GSMUA_Achr8G17480_001|GSMUA_Achr8G02340_001|GSMUA_Achr4G05780_001|GSMUA_Achr10G06250_001|GSMUA_Achr11G08130_001
Endocytosis	KEGG PATHWAY	mus04144	2	214	0.815427	0.87851	GSMUA_AchrUn_randomG01480_001|GSMUA_Achr9G12760_001
Glycerophospholipid metabolism	KEGG PATHWAY	mus00564	1	119	0.821832	0.87851	GSMUA_Achr5G20960_001
Plant-pathogen interaction	KEGG PATHWAY	mus04626	2	227	0.840134	0.882853	GSMUA_Achr2G20880_001|GSMUA_Achr4G18830_001
Glycolysis / Gluconeogenesis	KEGG PATHWAY	mus00010	1	164	0.907076	0.937312	GSMUA_Achr11G18240_001
Protein processing in endoplasmic reticulur	KEGG PATHWAY	mus04141	2	302	0.932719	0.94801	GSMUA_Achr3G26430_001|GSMUA_Achr2G13250_001
Spliceosome	KEGG PATHWAY	mus03040	1	229	0.963854	0.963854	GSMUA_Achr3G26010_001

Term	Database	ID	Input numl	Backgroun	P-Value	Corrected	Gene IDs
Purine metabolism	KEGG PATHWAY	mus00230	9	193	0.004896	0.102591	GSMUA_AchrUn_randomG26160_001|GSMUA_Achr2G06220_001|GSMUA_Achr8G18490_001|GSMUA_Achr1G14080_001|GSMUA_Achr3G13570_001|GSMUA_Achr3G19800_001|GSMUA_Achr3G25870_001|GSMUA_Achr8G03300_001|GSMUA_Achr8G26890_001
Peroxisome	KEGG PATHWAY	mus04146	6	97	0.005868	0.102591	GSMUA_Achr4G07060_001|GSMUA_Achr4G09570_001|GSMUA_Achr11G18410_001|GSMUA_Achr2G02380_001|GSMUA_Achr8G30270_001|GSMUA_Achr1G13190_001
Sulfur metabolism	KEGG PATHWAY	mus00920	4	44	0.006889	0.102591	GSMUA_Achr1G14080_001|GSMUA_Achr8G26890_001|GSMUA_Achr1G22900_001|GSMUA_Achr3G13570_001
Selenocompound metabolism	KEGG PATHWAY	mus00450	3	22	0.006955	0.102591	GSMUA_Achr1G14080_001|GSMUA_Achr8G26890_001|GSMUA_Achr3G13570_001
Circadian rhythm - plant	KEGG PATHWAY	mus04712	5	86	0.014691	0.173355	GSMUA_Achr6G16850_001|GSMUA_Achr5G16060_001|GSMUA_Achr6G34710_001|GSMUA_Achr5G01690_001|GSMUA_Achr9G09590_001
Ribosome biogenesis in eukaryotes	KEGG PATHWAY	mus03008	5	96	0.022073	0.217056	GSMUA_Achr6G16850_001|GSMUA_Achr5G16060_001|GSMUA_Achr6G34710_001|GSMUA_Achr5G01690_001|GSMUA_Achr9G09590_001
Pyruvate metabolism	KEGG PATHWAY	mus00620	5	108	0.033676	0.283838	GSMUA_AchrUn_randomG26160_001|GSMUA_Achr5G02000_001|GSMUA_Achr10G19000_001|GSMUA_Achr6G19750_001|GSMUA_AchrUn_randomG11210_001
Nicotinate and nicotinamide metabolism	KEGG PATHWAY	mus00760	2	23	0.058948	0.365831	GSMUA_Achr3G25870_001|GSMUA_Achr8G03300_001
Fatty acid biosynthesis	KEGG PATHWAY	mus00061	3	54	0.061377	0.365831	GSMUA_Achr10G19000_001|GSMUA_Achr1G13190_001|GSMUA_Achr8G30270_001
RNA transport	KEGG PATHWAY	mus03013	7	214	0.062005	0.365831	GSMUA_Achr3G26010_001|GSMUA_Achr9G13950_001|GSMUA_Achr1G28070_001|GSMUA_Achr5G07670_001|GSMUA_Achr6G11580_001|GSMUA_Achr5G26490_001|GSMUA_Achr10G00250_001
Metabolic pathways	KEGG PATHWAY	mus01100	45	2344	0.114705	0.615234	GSMUA_Achr5G08780_001|GSMUA_Achr3G04930_001|GSMUA_Achr7G03670_001|GSMUA_Achr10G27090_001|GSMUA_Achr8G24810_001|GSMUA_Achr2G17330_001|GSMUA_Achr4G09570_001|GSMUA_Achr3G19800_001|GSMUA_Achr11G20390_001|GSMUA_Achr1G22900_001|GSMUA_AchrUn_randomG16250_001|GSMUA_Achr8G17110_001|GSMUA_Achr3G13980_001|GSMUA_Achr1G14080_001|GSMUA_Achr1G22400_001|GSMUA_Achr3G25870_001|GSMUA_AchrUn_randomG15230_001|GSMUA_Achr3G13570_001|GSMUA_Achr3G24160_001|GSMUA_Achr3G23340_001|GSMUA_Achr10G23010_001|GSMUA_Achr8G18490_001|GSMUA_Achr7G00440_001|GSMUA_Achr5G07520_001|GSMUA_Achr11G09200_001|GSMUA_Achr9G11910_001|GSMUA_Achr8G03300_001|GSMUA_Achr4G32620_001|GSMUA_Achr5G02000_001|GSMUA_Achr9G11940_001|GSMUA_AchrUn_randomG11210_001|GSMUA_AchrUn_randomG26160_001|GSMUA_Achr2G06220_001|GSMUA_Achr8G28000_001|GSMUA_AchrUn_randomG26070_001|GSMUA_Achr8G22970_001|GSMUA_AchrUn_randomG26330_001|GSMUA_Achr7G03660_001|GSMUA_Achr7G19370_001|GSMUA_Achr8G26890_001|GSMUA_Achr8G30270_001|GSMUA_Achr6G35540_001|GSMUA_Achr5G03350_001|GSMUA_Achr10G19000_001|GSMUA_Achr1G13190_001
Glyoxylate and dicarboxylate metabolisr	KEGG PATHWAY	mus00630	3	77	0.133341	0.655595	GSMUA_AchrUn_randomG11210_001|GSMUA_Achr5G02000_001|GSMUA_Achr3G23560_001
Fatty acid degradation	KEGG PATHWAY	mus00071	2	45	0.170299	0.703603	GSMUA_Achr1G13190_001|GSMUA_Achr8G30270_001
Nitrogen metabolism	KEGG PATHWAY	mus00910	2	47	0.181644	0.703603	GSMUA_Achr8G28000_001|GSMUA_Achr5G05830_001
Porphyrin and chlorophyll metabolism	KEGG PATHWAY	mus00860	2	49	0.193098	0.703603	GSMUA_Achr8G17110_001|GSMUA_AchrUn_randomG16250_001
Pyrimidine metabolism	KEGG PATHWAY	mus00240	4	142	0.200943	0.703603	GSMUA_Achr3G25870_001|GSMUA_Achr8G03300_001|GSMUA_Achr2G06220_001|GSMUA_Achr8G22970_001
Fatty acid metabolism	KEGG PATHWAY	mus01212	3	95	0.202733	0.703603	GSMUA_Achr10G19000_001|GSMUA_Achr1G13190_001|GSMUA_Achr8G30270_001
Carbon fixation in photosynthetic organi	KEGG PATHWAY	mus00710	3	102	0.231679	0.712615	GSMUA_AchrUn_randomG11210_001|GSMUA_Achr5G02000_001|GSMUA_Achr11G09200_001
Alanine, aspartate and glutamate metabo	KEGG PATHWAY	mus00250	2	60	0.257282	0.712615	GSMUA_Achr8G28000_001|GSMUA_Achr11G09200_001
Phenylalanine, tyrosine and tryptophan b	KEGG PATHWAY	mus00400	2	65	0.286687	0.712615	GSMUA_Achr7G03660_001|GSMUA_Achr7G03670_001
Protein export	KEGG PATHWAY	mus03060	2	67	0.298424	0.712615	GSMUA_Achr4G11940_001|GSMUA_Achr11G21230_001
Citrate cycle (TCA cycle)	KEGG PATHWAY	mus00020	2	68	0.304282	0.712615	GSMUA_AchrUn_randomG11210_001|GSMUA_Achr5G02000_001
mRNA surveillance pathway	KEGG PATHWAY	mus03015	4	173	0.307725	0.712615	GSMUA_AchrUn_randomG02030_001|GSMUA_Achr9G28750_001|GSMUA_Achr3G26010_001|GSMUA_Achr7G09580_001
Inositol phosphate metabolism	KEGG PATHWAY	mus00562	2	70	0.31597	0.712615	GSMUA_Achr10G23010_001|GSMUA_Achr1G22400_001
Glycosaminoglycan degradation	KEGG PATHWAY	mus00531	1	23	0.318352	0.712615	GSMUA_Achr4G32620_001
Cysteine and methionine metabolism	KEGG PATHWAY	mus00270	3	128	0.343429	0.712615	GSMUA_AchrUn_randomG11210_001|GSMUA_Achr5G02000_001|GSMUA_Achr7G00440_001
Oxidative phosphorylation	KEGG PATHWAY	mus00190	4	183	0.3436	0.712615	GSMUA_Achr5G08780_001|GSMUA_AchrUn_randomG15230_001|GSMUA_Achr2G04890_001|GSMUA_Achr10G27090_001
Cutin, suberine and wax biosynthesis	KEGG PATHWAY	mus00073	1	26	0.350268	0.712615	GSMUA_Achr9G08080_001
Pantothenate and CoA biosynthesis	KEGG PATHWAY	mus00770	1	26	0.350268	0.712615	GSMUA_Achr8G22970_001
Galactose metabolism	KEGG PATHWAY	mus00052	2	81	0.379242	0.745842	GSMUA_Achr11G14030_001|GSMUA_Achr3G23340_001
Phosphatidylinositol signaling system	KEGG PATHWAY	mus04070	2	84	0.396096	0.749252	GSMUA_Achr10G23010_001|GSMUA_Achr1G22400_001
Lysine degradation	KEGG PATHWAY	mus00310	1	33	0.419073	0.749252	GSMUA_Achr4G09570_001
Propanoate metabolism	KEGG PATHWAY	mus00640	1	33	0.419073	0.749252	GSMUA_Achr10G19000_001
Glycine, serine and threonine metabolisn	KEGG PATHWAY	mus00260	2	92	0.439964	0.75306	GSMUA_Achr4G09570_001|GSMUA_Achr3G23560_001
Endocytosis	KEGG PATHWAY	mus04144	4	214	0.454209	0.75306	GSMUA_Achr5G15250_001|GSMUA_AchrUn_randomG01480_001|GSMUA_Achr9G12760_001|GSMUA_Achr7G20210_001
Starch and sucrose metabolism	KEGG PATHWAY	mus00500	5	276	0.459494	0.75306	GSMUA_Achr3G04930_001|GSMUA_Achr3G24160_001|GSMUA_Achr3G13980_001|GSMUA_Achr3G23340_001|GSMUA_Achr6G35540_001
Glutathione metabolism	KEGG PATHWAY	mus00480	2	102	0.492257	0.775208	GSMUA_Achr7G19370_001|GSMUA_Achr8G24810_001
RNA polymerase	KEGG PATHWAY	mus03020	1	43	0.504972	0.775208	GSMUA_Achr2G06220_001
Phenylpropanoid biosynthesis	KEGG PATHWAY	mus00940	4	231	0.512425	0.775208	GSMUA_AchrUn_randomG26070_001|GSMUA_Achr9G11910_001|GSMUA_Achr9G11940_001|GSMUA_AchrUn_randomG26330_001
N-Glycan biosynthesis	KEGG PATHWAY	mus00510	1	51	0.564489	0.818881	GSMUA_Achr5G07520_001
beta-Alanine metabolism	KEGG PATHWAY	mus00410	1	52	0.571409	0.818881	GSMUA_Achr8G22970_001
Biosynthesis of amino acids	KEGG PATHWAY	mus01230	5	326	0.604626	0.818881	GSMUA_AchrUn_randomG26160_001|GSMUA_Achr8G28000_001|GSMUA_Achr11G09200_001|GSMUA_Achr7G03670_001|GSMUA_Achr7G03660_001
Homologous recombination	KEGG PATHWAY	mus03440	1	58	0.610691	0.818881	GSMUA_Achr2G13090_001
2-Oxocarboxylic acid metabolism	KEGG PATHWAY	mus01210	1	58	0.610691	0.818881	GSMUA_Achr11G09200_001
Terpenoid backbone biosynthesis	KEGG PATHWAY	mus00900	1	63	0.640673	0.839993	GSMUA_Achr5G03350_001
RNA degradation	KEGG PATHWAY	mus03018	2	145	0.679314	0.871086	GSMUA_Achr5G04250_001|GSMUA_Achr10G08440_001
Photosynthesis	KEGG PATHWAY	mus00195	1	73	0.693916	0.871086	GSMUA_Achr2G17330_001
Phenylalanine metabolism	KEGG PATHWAY	mus00360	2	159	0.726786	0.893341	GSMUA_Achr9G11910_001|GSMUA_Achr9G11940_001
Carbon metabolism	KEGG PATHWAY	mus01200	4	321	0.760352	0.905085	GSMUA_AchrUn_randomG26160_001|GSMUA_Achr5G02000_001|GSMUA_Achr11G09200_001|GSMUA_AchrUn_randomG11210_001
Fructose and mannose metabolism	KEGG PATHWAY	mus00051	1	90	0.767021	0.905085	GSMUA_Achr7G20550_001
Pentose and glucuronate interconversion	KEGG PATHWAY	mus00040	1	105	0.816933	0.945079	GSMUA_Achr6G35540_001
Biosynthesis of secondary metabolites	KEGG PATHWAY	mus01110	16	1235	0.847628	0.948161	GSMUA_AchrUn_randomG26160_001|GSMUA_AchrUn_randomG11210_001|GSMUA_Achr7G03670_001|GSMUA_Achr8G17110_001|GSMUA_Achr8G28000_001|GSMUA_AchrUn_randomG26070_001|GSMUA_Achr3G23560_001|GSMUA_AchrUn_randomG26330_001|GSMUA_Achr7G03660_001|GSMUA_Achr3G25870_001|GSMUA_Achr9G11910_001|GSMUA_Achr8G03300_001|GSMUA_Achr5G02000_001|GSMUA_Achr5G03350_001|GSMUA_Achr9G11940_001|GSMUA_AchrUn_randomG16250_001
Protein processing in endoplasmic reticu	KEGG PATHWAY	mus04141	3	302	0.865149	0.948161	GSMUA_Achr10G18020_001|GSMUA_Achr7G04540_001|GSMUA_Achr4G11940_001
Plant-pathogen interaction	KEGG PATHWAY	mus04626	2	227	0.880882	0.948161	GSMUA_Achr2G20880_001|GSMUA_Achr4G18830_001
Spliceosome	KEGG PATHWAY	mus03040	2	229	0.883879	0.948161	GSMUA_Achr9G12650_001|GSMUA_Achr3G26010_001
Glycolysis / Gluconeogenesis	KEGG PATHWAY	mus00010	1	164	0.92927	0.967192	GSMUA_AchrUn_randomG26160_001
Plant hormone signal transduction	KEGG PATHWAY	mus04075	4	453	0.934406	0.967192	GSMUA_AchrUn_randomG12610_001|GSMUA_Achr3G23850_001|GSMUA_Achr10G26310_001|GSMUA_Achr5G26930_001
Amino sugar and nucleotide sugar metab	KEGG PATHWAY	mus00520	1	188	0.952019	0.968433	GSMUA_Achr3G24160_001
Ribosome	KEGG PATHWAY	mus03010	1	453	0.99937	0.99937	GSMUA_Achr8G02340_001

Term	Database	ID	Input number	Background number	P-Value	Corrected P-Value	Gene IDs
Purine metabolism	KEGG PATHWAY	mus00230	8	193	0.022182	0.5083894	GSMUA_Achr2G06220_001|GSMUA_Achr8G18490_001|GSMUA_Achr3G00590_001|GSMUA_Achr3G19800_001|GSMUA_Achr3G25870_001|GSMUA_Achr8G20120_001|GSMUA_Achr8G03300_001|GSMUA_Achr9G01450_001
Pyrimidine metabolism	KEGG PATHWAY	mus00240	6	142	0.041477	0.5083894	GSMUA_Achr2G06220_001|GSMUA_Achr8G22970_001|GSMUA_Achr3G25870_001|GSMUA_Achr3G00590_001|GSMUA_Achr8G03300_001|GSMUA_Achr9G01450_001
Isoquinoline alkaloid biosynthesis	KEGG PATHWAY	mus00950	2	22	0.062653	0.5083894	GSMUA_Achr9G11130_001|GSMUA_Achr8G15520_001
Nicotinate and nicotinamide metab	KEGG PATHWAY	mus00760	2	23	0.067356	0.5083894	GSMUA_Achr3G25870_001|GSMUA_Achr8G03300_001
beta-Alanine metabolism	KEGG PATHWAY	mus00410	3	52	0.067494	0.5083894	GSMUA_Achr9G11130_001|GSMUA_Achr8G22970_001|GSMUA_Achr8G15520_001
Fatty acid biosynthesis	KEGG PATHWAY	mus00061	3	54	0.07347	0.5083894	GSMUA_Achr10G19000_001|GSMUA_Achr1G13190_001|GSMUA_Achr8G30270_001
Cysteine and methionine metabolis	KEGG PATHWAY	mus00270	5	128	0.077708	0.5083894	GSMUA_Achr3G16330_001|GSMUA_Achr9G18460_001|GSMUA_Achr9G01630_001|GSMUA_AchrUn_randomG11210_001|GSMUA_Achr7G00440_001
RNA transport	KEGG PATHWAY	mus03013	7	214	0.084976	0.5083894	GSMUA_Achr3G26010_001|GSMUA_Achr4G20270_001|GSMUA_Achr9G13950_001|GSMUA_Achr5G07670_001|GSMUA_Achr7G05430_001|GSMUA_Achr5G26490_001|GSMUA_Achr10G00250_001
mRNA surveillance pathway	KEGG PATHWAY	mus03015	6	173	0.086599	0.5083894	GSMUA_Achr3G26010_001|GSMUA_Achr4G20270_001|GSMUA_Achr8G06590_001|GSMUA_AchrUn_randomG02030_001|GSMUA_Achr6G00420_001|GSMUA_Achr7G05430_001
Tropane, piperidine and pyridine al	KEGG PATHWAY	mus00960	2	28	0.092465	0.5083894	GSMUA_Achr9G11130_001|GSMUA_Achr8G15520_001
Peroxisome	KEGG PATHWAY	mus04146	4	97	0.094484	0.5083894	GSMUA_Achr1G13190_001|GSMUA_Achr11G18410_001|GSMUA_Achr8G30270_001|GSMUA_Achr4G09570_001
Oxidative phosphorylation	KEGG PATHWAY	mus00190	6	183	0.105302	0.5083894	GSMUA_Achr5G08780_001|GSMUA_Achr9G21710_001|GSMUA_Achr3G13280_001|GSMUA_AchrUn_randomG15230_001|GSMUA_Achr2G04890_001|GSMUA_Achr1G26020_001
Protein export	KEGG PATHWAY	mus03060	3	67	0.117516	0.5083894	GSMUA_Achr5G20650_001|GSMUA_Achr4G11940_001|GSMUA_Achr11G21230_001
Metabolic pathways	KEGG PATHWAY	mus01100	48	2344	0.124336	0.5083894	GSMUA_Achr5G08780_001|GSMUA_Achr3G04930_001|GSMUA_Achr7G03670_001|GSMUA_Achr8G24810_001|GSMUA_Achr2G17330_001|GSMUA_Achr4G09570_001|GSMUA_Achr4G20510_001|GSMUA_Achr3G19800_001|GSMUA_Achr8G20120_001|GSMUA_Achr1G22900_001|GSMUA_AchrUn_randomG16250_001|GSMUA_Achr9G11130_001|GSMUA_Achr9G21710_001|GSMUA_Achr8G17110_001|GSMUA_Achr7G05110_001|GSMUA_Achr3G25870_001|GSMUA_AchrUn_randomG15230_001|GSMUA_Achr6G16490_001|GSMUA_Achr10G08050_001|GSMUA_Achr9G18460_001|GSMUA_Achr8G08790_001|GSMUA_Achr6G08010_001|GSMUA_Achr11G08860_001|GSMUA_Achr8G18490_001|GSMUA_Achr7G00440_001|GSMUA_Achr9G01630_001|GSMUA_Achr5G07520_001|GSMUA_Achr11G09200_001|GSMUA_Achr9G11910_001|GSMUA_Achr8G03300_001|GSMUA_Achr9G11940_001|GSMUA_AchrUn_randomG11210_001|GSMUA_Achr3G16330_001|GSMUA_Achr2G06220_001|GSMUA_Achr8G28000_001|GSMUA_AchrUn_randomG26070_001|GSMUA_Achr4G10640_001|GSMUA_Achr8G22970_001|GSMUA_AchrUn_randomG26330_001|GSMUA_Achr7G03660_001|GSMUA_Achr7G19370_001|GSMUA_Achr3G00590_001|GSMUA_Achr8G30270_001|GSMUA_Achr8G15520_001|GSMUA_Achr6G35540_001|GSMUA_Achr10G19000_001|GSMUA_Achr1G13190_001|GSMUA_Achr9G01450_001
Pyruvate metabolism	KEGG PATHWAY	mus00620	4	108	0.125014	0.5083894	GSMUA_AchrUn_randomG11210_001|GSMUA_Achr10G19000_001|GSMUA_Achr10G08050_001|GSMUA_AchrUn_randomG21290_001
Non-homologous end-joining	KEGG PATHWAY	mus03450	1	9	0.158264	0.550613662	GSMUA_Achr8G01100_001
RNA polymerase	KEGG PATHWAY	mus03020	2	43	0.179021	0.550613662	GSMUA_Achr3G00590_001|GSMUA_Achr2G06220_001
Tyrosine metabolism	KEGG PATHWAY	mus00350	2	43	0.179021	0.550613662	GSMUA_Achr9G11130_001|GSMUA_Achr8G15520_001
Sulfur metabolism	KEGG PATHWAY	mus00920	2	44	0.185177	0.550613662	GSMUA_Achr3G16330_001|GSMUA_Achr1G22900_001
Fatty acid degradation	KEGG PATHWAY	mus00071	2	45	0.191363	0.550613662	GSMUA_Achr1G13190_001|GSMUA_Achr8G30270_001
Nitrogen metabolism	KEGG PATHWAY	mus00910	2	47	0.203813	0.550613662	GSMUA_Achr8G28000_001|GSMUA_Achr5G05830_001
Fructose and mannose metabolism	KEGG PATHWAY	mus00051	3	90	0.212237	0.550613662	GSMUA_Achr7G05110_001|GSMUA_Achr11G08860_001|GSMUA_Achr4G20510_001
Porphyrin and chlorophyll metaboli	KEGG PATHWAY	mus00860	2	49	0.216351	0.550613662	GSMUA_Achr8G17110_001|GSMUA_AchrUn_randomG16250_001
Glycine, serine and threonine metal	KEGG PATHWAY	mus00260	3	92	0.221162	0.550613662	GSMUA_Achr9G11130_001|GSMUA_Achr4G09570_001|GSMUA_Achr8G15520_001
Amino sugar and nucleotide sugar r	KEGG PATHWAY	mus00520	5	188	0.234526	0.550613662	GSMUA_Achr8G32960_001|GSMUA_Achr7G05110_001|GSMUA_Achr8G08790_001|GSMUA_Achr11G08860_001|GSMUA_Achr4G20510_001
Fatty acid metabolism	KEGG PATHWAY	mus01212	3	95	0.234688	0.550613662	GSMUA_Achr10G19000_001|GSMUA_Achr1G13190_001|GSMUA_Achr8G30270_001
RNA degradation	KEGG PATHWAY	mus03018	4	145	0.250491	0.553994449	GSMUA_Achr6G31430_001|GSMUA_Achr5G04250_001|GSMUA_Achr7G05430_001|GSMUA_Achr4G20270_001
Sphingolipid metabolism	KEGG PATHWAY	mus00600	2	55	0.254293	0.553994449	GSMUA_Achr6G16490_001|GSMUA_Achr6G08010_001
Glutathione metabolism	KEGG PATHWAY	mus00480	3	102	0.266761	0.555791045	GSMUA_Achr7G19370_001|GSMUA_Achr8G24810_001|GSMUA_Achr9G01450_001
Homologous recombination	KEGG PATHWAY	mus03440	2	58	0.27334	0.555791045	GSMUA_Achr8G01100_001|GSMUA_Achr2G13090_001
Alanine, aspartate and glutamate m	KEGG PATHWAY	mus00250	2	60	0.28603	0.562833995	GSMUA_Achr8G28000_001|GSMUA_Achr11G09200_001
Phenylalanine metabolism	KEGG PATHWAY	mus00360	4	159	0.303606	0.578748453	GSMUA_Achr9G11130_001|GSMUA_Achr9G11910_001|GSMUA_Achr9G11940_001|GSMUA_Achr8G15520_001
Phenylalanine, tyrosine and tryptop	KEGG PATHWAY	mus00400	2	65	0.31763	0.587134254	GSMUA_Achr7G03660_001|GSMUA_Achr7G03670_001
Citrate cycle (TCA cycle)	KEGG PATHWAY	mus00020	2	68	0.336442	0.603616313	GSMUA_AchrUn_randomG11210_001|GSMUA_Achr10G08050_001
Pantothenate and CoA biosynthesis	KEGG PATHWAY	mus00770	1	26	0.372164	0.648628762	GSMUA_Achr8G22970_001
Lysine degradation	KEGG PATHWAY	mus00310	1	33	0.443623	0.73137926	GSMUA_Achr4G09570_001
Propanoate metabolism	KEGG PATHWAY	mus00640	1	33	0.443623	0.73137926	GSMUA_Achr10G19000_001
Carbon fixation in photosynthetic o	KEGG PATHWAY	mus00710	2	102	0.532847	0.855359603	GSMUA_AchrUn_randomG11210_001|GSMUA_Achr11G09200_001
Phenylpropanoid biosynthesis	KEGG PATHWAY	mus00940	4	231	0.572447	0.891681186	GSMUA_AchrUn_randomG26070_001|GSMUA_Achr9G11910_001|GSMUA_Achr9G11940_001|GSMUA_AchrUn_randomG26330_001
N-Glycan biosynthesis	KEGG PATHWAY	mus00510	1	51	0.592339	0.891681186	GSMUA_Achr5G07520_001
SNARE interactions in vesicular tra	KEGG PATHWAY	mus04130	1	52	0.599327	0.891681186	GSMUA_Achr10G28310_001
Flavonoid biosynthesis	KEGG PATHWAY	mus00941	1	55	0.619584	0.899871293	GSMUA_Achr4G10640_001
2-Oxocarboxylic acid metabolism	KEGG PATHWAY	mus01210	1	58	0.63882	0.906233664	GSMUA_Achr11G09200_001
Biosynthesis of amino acids	KEGG PATHWAY	mus01230	5	326	0.670869	0.914567081	GSMUA_Achr3G16330_001|GSMUA_Achr8G28000_001|GSMUA_Achr11G09200_001|GSMUA_Achr7G03670_001|GSMUA_Achr7G03660_001
Plant hormone signal transduction	KEGG PATHWAY	mus04075	7	453	0.674681	0.914567081	GSMUA_Achr2G06770_001|GSMUA_Achr10G10440_001|GSMUA_Achr5G26930_001|GSMUA_Achr8G02300_001|GSMUA_AchrUn_randomG12610_001|GSMUA_Achr3G23850_001|GSMUA_Achr10G26310_001
Starch and sucrose metabolism	KEGG PATHWAY	mus00500	4	276	0.708104	0.916791823	GSMUA_Achr3G04930_001|GSMUA_Achr8G08790_001|GSMUA_Achr11G08860_001|GSMUA_Achr6G35540_001
Photosynthesis	KEGG PATHWAY	mus00195	1	73	0.72141	0.916791823	GSMUA_Achr2G17330_001
Proteasome	KEGG PATHWAY	mus03050	1	73	0.72141	0.916791823	GSMUA_Achr4G00300_001
Glyoxylate and dicarboxylate metal	KEGG PATHWAY	mus00630	1	77	0.740059	0.917680324	GSMUA_AchrUn_randomG11210_001
Protein processing in endoplasmic r	KEGG PATHWAY	mus04141	4	302	0.77088	0.917680324	GSMUA_Achr10G18020_001|GSMUA_Achr7G04540_001|GSMUA_Achr4G11940_001|GSMUA_Achr2G13250_001
Circadian rhythm - plant	KEGG PATHWAY	mus04712	1	86	0.777598	0.917680324	GSMUA_AchrUn_randomG01410_001
Arginine and proline metabolism	KEGG PATHWAY	mus00330	1	94	0.806407	0.917680324	GSMUA_Achr9G18460_001
Carbon metabolism	KEGG PATHWAY	mus01200	4	321	0.80972	0.917680324	GSMUA_Achr3G16330_001|GSMUA_AchrUn_randomG11210_001|GSMUA_Achr11G09200_001|GSMUA_Achr10G08050_001
Biosynthesis of secondary metaboli	KEGG PATHWAY	mus01110	18	1235	0.812373	0.917680324	GSMUA_AchrUn_randomG11210_001|GSMUA_Achr7G03660_001|GSMUA_Achr7G03670_001|GSMUA_Achr8G20120_001|GSMUA_Achr9G11130_001|GSMUA_Achr8G28000_001|GSMUA_AchrUn_randomG26070_001|GSMUA_Achr4G10640_001|GSMUA_Achr10G08050_001|GSMUA_Achr8G32960_001|GSMUA_Achr3G25870_001|GSMUA_Achr9G11910_001|GSMUA_Achr8G03300_001|GSMUA_AchrUn_randomG26330_001|GSMUA_Achr8G15520_001|GSMUA_Achr8G17110_001|GSMUA_Achr9G11940_001|GSMUA_AchrUn_randomG16250_001
Pentose and glucuronate interconve	KEGG PATHWAY	mus00040	1	105	0.840049	0.931690292	GSMUA_Achr6G35540_001
Endocytosis	KEGG PATHWAY	mus04144	2	214	0.886837	0.96601836	GSMUA_AchrUn_randomG01480_001|GSMUA_Achr9G12760_001
Spliceosome	KEGG PATHWAY	mus03040	2	229	0.908112	0.971839048	GSMUA_Achr3G06150_001|GSMUA_Achr3G26010_001
Glycolysis / Gluconeogenesis	KEGG PATHWAY	mus00010	1	164	0.942701	0.980951246	GSMUA_Achr10G08050_001
Ribosome	KEGG PATHWAY	mus03010	4	453	0.955563	0.980951246	GSMUA_Achr10G02910_001|GSMUA_Achr4G09610_001|GSMUA_Achr10G14200_001|GSMUA_Achr4G17350_001
Ubiquitin mediated proteolysis	KEGG PATHWAY	mus04120	1	193	0.965464	0.980951246	GSMUA_AchrUn_randomG01410_001
Plant-pathogen interaction	KEGG PATHWAY	mus04626	1	227	0.980951	0.980951246	GSMUA_Achr4G18830_001

Supplemental data S8. The significantly enriched GO terms identified in/between different comparisons. Comparison 1: R5H VS RCK, Comparison 2: R10 vs RCK, and Comparison 3: R25 vs RCK.

Comparison 1 specific	Comparison 2 specific	Comparison 3 specific	Common in Comparison 1 and 2	Common in Comparison 1 and 3	Common in Comparison 2 and 3	Common in Comparison 1, 2 and 3
Fructose and mannose metabolism	Alanine, aspartate and glutamate metabolism	beta-Alanine metabolism	Fatty acid metabolism	Cysteine and methionine metabolism	Purine metabolism	Fatty acid biosynthesis
Glycine, serine and threonine metabolism	Carbon fixation in photosynthetic organisms	Isoquinoline alkaloid biosynthesis	Phenylalanine, tyrosine and tryptophan biosynthesis	mRNA surveillance pathway	RNA transport	Fatty acid degradation
Lysine biosynthesis	Circadian rhythm - plant	Non-homologous end-joining		Protein export		Metabolic pathways
Propanoate metabolism	Glyoxylate and dicarboxylate metabolism	Oxidative phosphorylation				Nicotinate and nicotinamide metabolism
SNARE interactions in vesicular transport	Nitrogen metabolism	RNA polymerase				Peroxisome
Synthesis and degradation of ketone bodies	Porphyrin and chlorophyll metabolism	Tropane, piperidine and pyridine alkaloid biosynthesis				Pyrimidine metabolism
Terpenoid backbone biosynthesis	Ribosome biogenesis in eukaryotes	Tyrosine metabolism				Pyruvate metabolism
	Selenocompound metabolism					Sulfur metabolism

Supplemental data S9. Some enriched pathways of the predicted target genes of DE miRNAs. 1: R5H vs RCK, 2: R10 vs RCK, 3: R25 vs RCK.

Target Gene IDs	Target gene annotation	Up-regulated miRNA	Down-regulated miRNA
**Peroxisome**			
GSMUA_Achr4G07060_001	protein SYM1-like isoform X1	mac-miR398[1,2]，mac-miR398a-3p.1[1,2]	
GSMUA_Achr4G09570_001	probable sarcosine oxidase	mac-miR160d-3p[1,2,3]	
GSMUA_Achr11G18410_001	peroxisome biogenesis protein 1 isoform X1	mac-miR156a.1[1,2,3]	
GSMUA_Achr2G02380_001	superoxide dismutase	mac-miR398a-3p.1[1,2]，mac-miR398[1,2]	mac-miR398b-3p[1]
GSMUA_Achr8G30270_001	long chain acyl-CoA synthetase 9	mac-miR397.1[1,2,3]	
GSMUA_Achr1G13190_001	long chain acyl-CoA synthetase 9	mac-miR397.1[1,2,3]	
**Fatty acid biosynthesis**			
GSMUA_Achr8G24640_001	biotin carboxylase 2	mac-miR444a-3p.2[1]	
GSMUA_Achr10G19000_001	acetyl-CoA carboxylase 1	mac-miR396[3]	mac-miR396[2], mac-miR845[1,2,3]
GSMUA_Achr1G13190_001	long chain acyl-CoA synthetase 9	mac-miR397.1[1,2,3]	
GSMUA_Achr8G30270_001	long chain acyl-CoA synthetase 9	mac-miR397.1[1,2,3]	
**Fatty acid degradation**			
GSMUA_Achr1G13190_001	long chain acyl-CoA synthetase 9	mac-miR397.1[1,2,3]	
GSMUA_Achr8G30270_001	long chain acyl-CoA synthetase 9	mac-miR397.1[1,2,3]	
**Sulfur metabolism**			
GSMUA_Achr3G16330_001	probable serine acetyltransferase 1	mac-miR171a.2[3], mac-miR171c.1[3], mac-miR171b.2[3]	mac-miR171c.1[1]
GSMUA_Achr1G22900_001	sulfite reductase [ferredoxin]	mac-miR172c.2[2,3], mac-miR172e-3p.1[1,3], mac-miR172b[1,3], mac-miR172e-3p.2[1,3]	mac-miR172c.1[1], mac-miR172c-3p[2], mac-miR172a.1[2]
GSMUA_Achr1G14080_001	ATP sulfurylase 1	mac-miR395t[2]	
GSMUA_Achr8G26890_001	ATP sulfurylase 1	mac-miR395t[2]	
GSMUA_Achr1G22900_001	sulfite reductase [ferredoxin]	mac-miR172c.2[2,3], mac-miR172e-3p.1[1,3], mac-miR172b[1,3], mac-miR172e-3p.2[1,3]	mac-miR172c.1[1], mac-miR172c-3p[2], mac-miR172a.1[2]
GSMUA_Achr3G13570_00	ATP sulfurylase 1	mac-miR395t[2]	
**Nicotinate and nicotinamide metabolism**			
GSMUA_Achr3G25870_001	5-nucleotidase surE	mac-miR397a.2[1,2,3]	
GSMUA_Achr8G03300_001	E3 ubiquitin-protein ligase	mac-miR397a.1[1,2,3]	
**Pyrimidine metabolism**			
GSMUA_Achr3G25870_001	5-nucleotidase surE	mac-miR397a.2[1,2,3]	
GSMUA_Achr9G23310_001	DNA-directed RNA polymerases II, IV and V subunit 8B-like	mac-miR5234[1]	
GSMUA_Achr8G03300_001	uncharacterized protein	mac-miR397a.1[1,2,3]	
GSMUA_Achr9G01450_001	ribonucleoside-diphosphate reductase small chain-like	mac-miR408-5p[1,3]	
GSMUA_Achr2G06220_001	DNA-directed RNA polymerase III subunit rpc6-like	mac-miR396[3]	mac-miR396c-3p[2], mac-miR396[2]
GSMUA_Achr8G22970_001	dihydropyrimidine dehydrogenase [NADP(+)]	mac-miR529-5p[2,3]	
GSMUA_Achr3G00590_001	DNA-directed RNA polymerase II subunit 1-like		mac-miR172f[3]
**Pyruvate metabolism**			
GSMUA_AchrUn_randomG11210_001	malate dehydrogenase, mitochondrial	mac-miR156h[2], mac-miR156a.1[1,2,3]	
GSMUA_Achr8G24640_001	biotin carboxylase 2, chloroplastic-like isoform X1	mac-miR444a-3p.2[1]	
GSMUA_Achr10G19000_001	acetyl-CoA carboxylase 1	mac-miR396[3]	mac-miR845[1,2,3], mac-miR396[2]
GSMUA_AchrUn_randomG26160_001	pyruvate kinase isozyme G, chloroplastic-like, partial		mac-miR530-5p[2]
GSMUA_Achr5G02000_001	malate dehydrogenase, glyoxysomal	mac-miR156h[2]	
GSMUA_Achr6G19750_001	lactoylglutathione lyase-like		mac-miR5368.1[2]
GSMUA_Achr10G08050_001	dihydrolipoyllysine-residue acetyltransferase component 3 of pyruvate dehydrogenase complex	mac-miR172j[3]	
GSMUA_AchrUn_randomG21290_001	hydroxyacylglutathione hydrolase 2	mac-miR172j[3]	
**Metabolic pathways**			
GSMUA_AchrUn_randomG11210_001	malate dehydrogenase	mac-miR156h[2], mac-miR156a.1[1,2,3]	
GSMUA_Achr3G04930_001	probable alpha,alpha-trehalose-phosphate synthase	mac-miR172b[1,3]	mac-miR172a.1[2], mac-miR172c-3p[2], mac-miR172c.1[1], mac-miR172f[3]
GSMUA_Achr7G03670_001	bifunctional 3-dehydroquinate dehydratase/shikimate dehydrogenase, chloroplastic-like isoform X2	mac-miR397.4[1,2,3], mac-miR397.1[1,2,3]	
GSMUA_Achr8G26330_001	cytochrome P450 90B1-like		mac-miR529a[1]
GSMUA_Achr10G27090_001	putative cytochrome c oxidase subunit 5b-like		mac-miR398a-3p.2[1,2]
GSMUA_Achr1G22400_001	phosphatidylinositol 4-kinase alpha 1	mac-miR3711[2]	mac-miR164[1,2]
GSMUA_Achr2G17330_001	oxygen-evolving enhancer protein 2	mac-miR160d-3p[1,2,3], mac-miR160b.2[1,2], mac-miR160h[1,3], mac-miR160a-3p[1,2,3]	
GSMUA_Achr4G09570_001	probable sarcosine oxidase	mac-miR160d-3p[1,2,3]	
GSMUA_Achr4G20510_001	probable GDP-L-fucose synthase 1	mac-miR408-5p[1,3]	
GSMUA_Achr1G22900_001	sulfite reductase [ferredoxin]	mac-miR172c.2[2,3], mac-miR172e-3p.1[1,3], mac-miR172b[1,3], mac-miR172e-3p.2[1,3]	mac-miR172c.1[1], mac-miR172c-3p[2], mac-miR172a.1[2]
GSMUA_Achr8G24640_001	biotin carboxylase 2, chloroplastic-like isoform X1	mac-miR444a-3p.2[1]	
GSMUA_Achr7G03660_001	bifunctional 3-dehydroquinate dehydratase/shikimate dehydrogenase	mac-miR397.2[1,2,3], mac-miR397.4[1,2,3], mac-miR397.1[1,2,3]	
GSMUA_Achr7G05110_001	probable GDP-L-fucose synthase 1	mac-miR408-5p[1,3]	
GSMUA_Achr3G25870_001	uncharacterized protein	mac-miR397a.2[1,2,3]	
GSMUA_Achr9G00950_001	hydroxymethylglutaryl-CoA synthase-like	mac-miR5234[1]	
GSMUA_Achr4G16070_001	sucrose-phosphate synthase	mac-miR5658[1]	
GSMUA_Achr3G23340_001	probable alpha-glucosidase		mac-miR162.2[1,2]
GSMUA_Achr9G18460_001	S-adenosylmethionine decarboxylase proenzyme	mac-miR408-3p.3[1,3], mac-miR408d[1,3], mac-miR408b[1,3]	
GSMUA_Achr10G23010_001	phosphoinositide phospholipase C 2-like		mac-miR156a.2[1,2], mac-miR156b.2[1,2]
GSMUA_Achr5G07520_001	probable dolichyl pyrophosphate Man9GlcNAc2 alpha-1,3-glucosyltransferase	mac-miR156a.1[1,2,3]	
GSMUA_Achr8G29090_001	glutathione S-transferase zeta class-like	mac-miR5658[1]	
GSMUA_Achr4G28160_001	aspartokinase 3		mac-miR4995[1]
GSMUA_Achr8G03300_001	uncharacterized protein	mac-miR397a.1[1,2,3]	
GSMUA_Achr4G32620_001	heparanase-like protein 1		mac-miR156b.2[1,2], mac-miR156g.2[1,2]
GSMUA_Achr11G18240_001	fructose-1,6-bisphosphatase	mac-miR5658[1]	
GSMUA_Achr3G16330_001	probable serine acetyltransferase 1	mac-miR171a.2[3], mac-miR171c.1[3], mac-miR171b.2[3]	mac-miR171c.1[1]
GSMUA_Achr8G08790_001	UDP-glucuronate 4-epimerase 6-like	mac-miR408-5p[1,3]	
GSMUA_AchrUn_randomG26070_001	tricetin 3',4',5'-O-trimethyltransferase-like	mac-miR156c-3p.2[1,3], mac-miR172e-3p.1[1,3], mac-miR156b-3p.1[1,3]	mac-miR172a.1[2], mac-miR172c-3p[2], mac-miR172c.1[1]
GSMUA_Achr5G20960_001	glycerol-3-phosphate acyltransferase 3-like	mac-miR5658[1]	
GSMUA_AchrUn_randomG26330_001	flavone O-methyltransferase 1-like isoform X1	mac-miR156c-3p.2[1,3], mac-miR172e-3p.1[1,3], mac-miR156b-3p.1[1,3]	mac-miR172a.1[2], mac-miR172c-3p[2], mac-miR172c.1[1]
GSMUA_Achr2G05090_001	hypothetical protein	mac-miR5658[1]	
GSMUA_Achr2G04860_001	tricetin 3',4',5'-O-trimethyltransferase-like		mac-miR529a[1]
GSMUA_Achr9G23310_001	DNA-directed RNA polymerases II, IV and V subunit 8B-like	mac-miR5234[1]	
GSMUA_Achr8G30270_001	long chain acyl-CoA synthetase 9	mac-miR397.1[1,2,3]	
GSMUA_Achr6G35540_001	pectinesterase-like	mac-miR397.2[1,2,3], mac-miR397.4[1,2,3], mac-miR397.1[1,2,3], mac-miR397b.1[1,2,3], mac-miR397b.2[1,2,3], mac-miR397a.3[1,2,3], mac-miR397a.1[1,2,3], mac-miR397.3[1]	
GSMUA_Achr5G03350_001	2-C-methyl-D-erythritol 2,4-cyclodiphosphate synthase		mac-miR156b.2[1,2]
GSMUA_Achr10G19000_001	acetyl-CoA carboxylase 1	mac-miR396[3]	mac-miR845[1,2,3],mac-miR396[2]
GSMUA_Achr1G13190_001	long chain acyl-CoA synthetase 9	mac-miR397.1[1,2,3]	
GSMUA_Achr9G01450_001	ribonucleoside-diphosphate reductase small chain-like	mac-miR408-5p[1,3]	
GSMUA_Achr5G08780_001	cytochrome c oxidase subunit 6a	mac-miR396[3]	mac-miR396[2]
GSMUA_Achr8G24810_001	leucine aminopeptidase 1-like	mac-miR169c-3p[3], mac-miR169a-3p[2,3], mac-miR169h-3p[3]	
GSMUA_Achr3G19800_001	adenine phosphoribosyltransferase 4-like	mac-miR396[3]	mac-miR396[2]
GSMUA_Achr11G20390_001	DNA polymerase zeta catalytic subunit	mac-miR156h[2]	
GSMUA_AchrUn_randomG16250_001	glutamyl-tRNA reductase 2-like	mac-miR396[3]	mac-miR396[2]
GSMUA_Achr8G17110_001	magnesium-chelatase subunit ChlD	mac-miR169h-3p[3], mac-miR169a-3p[2,3]	
GSMUA_Achr3G13980_001	sucrose synthase 7-like		mac-miR5368.1[2]
GSMUA_Achr1G14080_001	ATP sulfurylase 1	mac-miR395t[2]	
GSMUA_AchrUn_randomG15230_001	ATP synthase subunit alpha		mac-miR5141[2,3]
GSMUA_Achr3G13570_001	ATP sulfurylase 1	mac-miR395t[2]	
GSMUA_Achr3G24160_001	cellulose synthase-like protein D5		mac-miR164c-5p[2]
GSMUA_Achr8G18490_001	adenine phosphoribosyltransferase 4-like	mac-miR396[3]	mac-miR396[2]
GSMUA_Achr7G00440_001	DNA (cytosine-5)-methyltransferase 1B-like	mac-miR396[3]	mac-miR396[2]
GSMUA_Achr11G09200_001	alanine aminotransferase 2	mac-miR393a.1[2,3]	
GSMUA_Achr9G11910_001	peroxidase 1-like	mac-miR529-5p[2,3]	
GSMUA_Achr5G02000_001	malate dehydrogenase, glyoxysomal	mac-miR156h[2]	
GSMUA_Achr9G11940_001	peroxidase 1	mac-miR529-5p[2,3]	
GSMUA_AchrUn_randomG26160_001	pyruvate kinase isozyme G		mac-miR530-5p[2]
GSMUA_Achr2G06220_001	DNA-directed RNA polymerase III subunit rpc6-like	mac-miR396[3]	mac-miR396[2],mac-miR396c-3p[2]
GSMUA_Achr8G28000_001	glutamate synthase 1 [NADH]	mac-miR169v[2], mac-miR169d-5p[3]	
GSMUA_Achr8G22970_001	dihydropyrimidine dehydrogenase [NADP(+)]	mac-miR529-5p[2,3]	
GSMUA_Achr7G19370_001	leucine aminopeptidase 2	mac-miR169c-3p[3], mac-miR169a-3p[2,3], mac-miR169h-3p[3]	
GSMUA_Achr8G26890_001	ATP sulfurylase 1	mac-miR395t[2]	
GSMUA_Achr8G20120_001	phosphoribosylaminoimidazole carboxylase	mac-miR172j[3]	
GSMUA_Achr9G11130_001	primary amine oxidase-like	mac-miR169c-3p[3]	
GSMUA_Achr9G21710_001	ATP synthase subunit beta	mac-miR169h-3p[3]	
GSMUA_Achr6G16490_001	long chain base biosynthesis protein 2a-like	mac-miR528-5p[3]	
GSMUA_Achr10G08050_001	dihydrolipoyllysine-residue acetyltransferase component 3 of pyruvate dehydrogenase complex, mitochondrial-like	mac-miR172j[3]	
GSMUA_Achr6G08010_001	long chain base biosynthesis protein 2a	mac-miR528-5p[3]	
GSMUA_Achr11G08860_001	fructokinase-1-like	mac-miR169c-3p[3]	
GSMUA_Achr9G01630_001	adenosylhomocysteinase	mac-miR172a-5p[3]	
GSMUA_Achr4G10640_001	dihydroflavonol-4-reductase-like	mac-miR5538.2[3]	
GSMUA_Achr3G00590_001	DNA-directed RNA polymerase II subunit 1-like		mac-miR172f[3]
GSMUA_Achr8G15520_001	primary amine oxidase-like	mac-miR169c-3p[3]	
**Plant hormone signal transduction**			
GSMUA_Achr2G06770_001	transcription factor TGA2-like isoform X1	mac-miR408-3p.2[1,3], mac-miR408d[1,3], mac-miR408-3p.3[1,3]	
GSMUA_Achr7G09190_001	regulatory protein NPR1-like	mac-miR5658[1]	
GSMUA_Achr10G10440_001	ABSCISIC ACID-INSENSITIVE 5-like protein 4	mac-miR408-3p.3[1,3]	
GSMUA_Achr5G26930_001	transport inhibitor response 1-like protein	mac-miR393a.1[2,3], mac-miR393a.2[2,3], mac-miR393a-5p[2,3]	mac-miR393-5p[1,2]
GSMUA_Achr3G23850_001	transport inhibitor response 1-like protein	mac-miR393a.1[2,3], mac-miR393a.2[2,3], mac-miR393a-5p[2,3]	mac-miR393-5p[1,2]
GSMUA_Achr10G26310_001	auxin-responsive protein IAA17-like	mac-miR156a.[1,2,3], mac-miR156h[2]	
GSMUA_AchrUn_randomG12610_001	two-component response regulator ARR17-like	mac-miR396[3]	mac-miR396[2]
GSMUA_Achr8G02300_001	auxin transporter-like protein 4	mac-miR169h-3p[3]	
**Plant-pathogen interaction**			
GSMUA_Achr2G20880_001	PTI1-like tyrosine-protein kinase 1	mac-miR398a-3p.1[1,2], mac-miR398[1,2]	mac-miR398b-3p[1]
GSMUA_Achr4G18830_001	putative calcium-binding protein CML19	mac-miR397.1[1,2,3]	
